# Invasive validation of novel 1D models for computation of coronary fractional flow reserve

**DOI:** 10.1093/cvr/cvaf168

**Published:** 2025-09-23

**Authors:** Daniel J Taylor, Harry Saxton, Xu Xu, Eron Yones, Louise Aubiniere-Robb, Thilanka Adikari, Tom Newman, Marcel van ‘t Veer, Daniëlle C J Keulards, Pim Tonino, Rebecca Gosling, Krzysztof Czechowicz, Andrew Narracott, David R Hose, Julian P Gunn, Ian Halliday, Paul D Morris

**Affiliations:** Division of Clinical Medicine, School of Medicine and Population Health, University of Sheffield, Beech Hill Road, Sheffield S10 2RX, UK; Insigneo Institute for in Silico Medicine, University of Sheffield, Sheffield, UK; NIHR Sheffield Biomedical Research Centre, Sheffield Teaching Hospitals NHS Foundation Trust, Sheffield, UK; Insigneo Institute for in Silico Medicine, University of Sheffield, Sheffield, UK; School of Computer Science, University of Sheffield, Sheffield, UK; Insigneo Institute for in Silico Medicine, University of Sheffield, Sheffield, UK; School of Computer Science, University of Sheffield, Sheffield, UK; Division of Clinical Medicine, School of Medicine and Population Health, University of Sheffield, Beech Hill Road, Sheffield S10 2RX, UK; Department of Cardiology and Cardiothoracic Surgery, Sheffield Teaching Hospitals NHS Trust, Sheffield, UK; Division of Clinical Medicine, School of Medicine and Population Health, University of Sheffield, Beech Hill Road, Sheffield S10 2RX, UK; Kingston and Richmond NHS Foundation Trust, London, UK; Division of Clinical Medicine, School of Medicine and Population Health, University of Sheffield, Beech Hill Road, Sheffield S10 2RX, UK; Insigneo Institute for in Silico Medicine, University of Sheffield, Sheffield, UK; NIHR Sheffield Biomedical Research Centre, Sheffield Teaching Hospitals NHS Foundation Trust, Sheffield, UK; Department of Cardiology and Cardiothoracic Surgery, Sheffield Teaching Hospitals NHS Trust, Sheffield, UK; Department of Cardiology, Catharina Hospital, Eindhoven, Netherlands; Department of Biomechanical Engineering, Eindhoven University of Technology, Eindhoven, Netherlands; Department of Cardiology, Catharina Hospital, Eindhoven, Netherlands; Department of Cardiology, Catharina Hospital, Eindhoven, Netherlands; Department of Biomechanical Engineering, Eindhoven University of Technology, Eindhoven, Netherlands; Division of Clinical Medicine, School of Medicine and Population Health, University of Sheffield, Beech Hill Road, Sheffield S10 2RX, UK; Insigneo Institute for in Silico Medicine, University of Sheffield, Sheffield, UK; NIHR Sheffield Biomedical Research Centre, Sheffield Teaching Hospitals NHS Foundation Trust, Sheffield, UK; Department of Cardiology and Cardiothoracic Surgery, Sheffield Teaching Hospitals NHS Trust, Sheffield, UK; Division of Clinical Medicine, School of Medicine and Population Health, University of Sheffield, Beech Hill Road, Sheffield S10 2RX, UK; Insigneo Institute for in Silico Medicine, University of Sheffield, Sheffield, UK; Division of Clinical Medicine, School of Medicine and Population Health, University of Sheffield, Beech Hill Road, Sheffield S10 2RX, UK; Insigneo Institute for in Silico Medicine, University of Sheffield, Sheffield, UK; Division of Clinical Medicine, School of Medicine and Population Health, University of Sheffield, Beech Hill Road, Sheffield S10 2RX, UK; Insigneo Institute for in Silico Medicine, University of Sheffield, Sheffield, UK; Division of Clinical Medicine, School of Medicine and Population Health, University of Sheffield, Beech Hill Road, Sheffield S10 2RX, UK; Insigneo Institute for in Silico Medicine, University of Sheffield, Sheffield, UK; NIHR Sheffield Biomedical Research Centre, Sheffield Teaching Hospitals NHS Foundation Trust, Sheffield, UK; Department of Cardiology and Cardiothoracic Surgery, Sheffield Teaching Hospitals NHS Trust, Sheffield, UK; Division of Clinical Medicine, School of Medicine and Population Health, University of Sheffield, Beech Hill Road, Sheffield S10 2RX, UK; Insigneo Institute for in Silico Medicine, University of Sheffield, Sheffield, UK; Division of Clinical Medicine, School of Medicine and Population Health, University of Sheffield, Beech Hill Road, Sheffield S10 2RX, UK; Insigneo Institute for in Silico Medicine, University of Sheffield, Sheffield, UK; NIHR Sheffield Biomedical Research Centre, Sheffield Teaching Hospitals NHS Foundation Trust, Sheffield, UK; Department of Cardiology and Cardiothoracic Surgery, Sheffield Teaching Hospitals NHS Trust, Sheffield, UK

**Keywords:** Computational fluid dynamics, Virtual fractional flow reserve, 1D modelling, Coronary artery disease, Percutaneous coronary intervention

## Abstract

**Aims:**

Computed virtual fractional flow reserve (vFFR), derived from invasive angiography, non-invasively quantifies coronary epicardial lesion physiology. Developments of 1-dimensional (1D) vFFR models have introduced methods of side-branch flow representation and reduced simulation time by several orders of magnitude vs. 3-dimensional (3D) alternatives. This study aimed to quantify agreement and diagnostic accuracy of 1D and 3D vFFR models, in a matched cohort, and compare results with established FFR alternatives.

**Methods and results:**

We used five 1D models, which differed in their side-branch flow representation, to compute vFFR in 104 arteries. The simplest model ignored side-branch flow, the second and third models used vessel anatomy to homogenously distribute side-branch flow and regionalize this to bifurcations, respectively. The final two 1D models additionally used simulated pressure in the main vessel to modulate side-branch flow magnitude. To aid interpretability, diagnostic accuracy was also reported for 3D vFFR, visual assessment and resting invasive pressure assessment (Pd/Pa). Median FFR was 0.81 [0.73–0.88] and 46 (44%) lesions were haemodynamically significant. Optimal FFR agreement was achieved with the 1D model that regionalized side-branch flow to bifurcations (mean bias at diagnostic threshold −0.03, 95% agreement limits −0.23 to 0.20). Diagnostic accuracy did not differ significantly between the five 1D models, with area under the curve (AUC) values ranging 0.68 to 0.74. Diagnostic accuracy for 1D vFFR was superior to visual assessment, comparable to 3D vFFR and poorer than invasive resting pressure assessment.

**Conclusion:**

1D models of vFFR facilitate rapid *in-silico* assessment of epicardial lesion severity. Inclusion of anatomical side branch flow mildly improved agreement, but the additional inclusion of simulated pressure was not beneficial. Agreement of 1D models was comparable to 3D simulations. However, current 1D models are not sufficiently accurate to suggest they may entirely replace wire-based assessment.

## Graphical Abstract

**Graphical Abstract:**
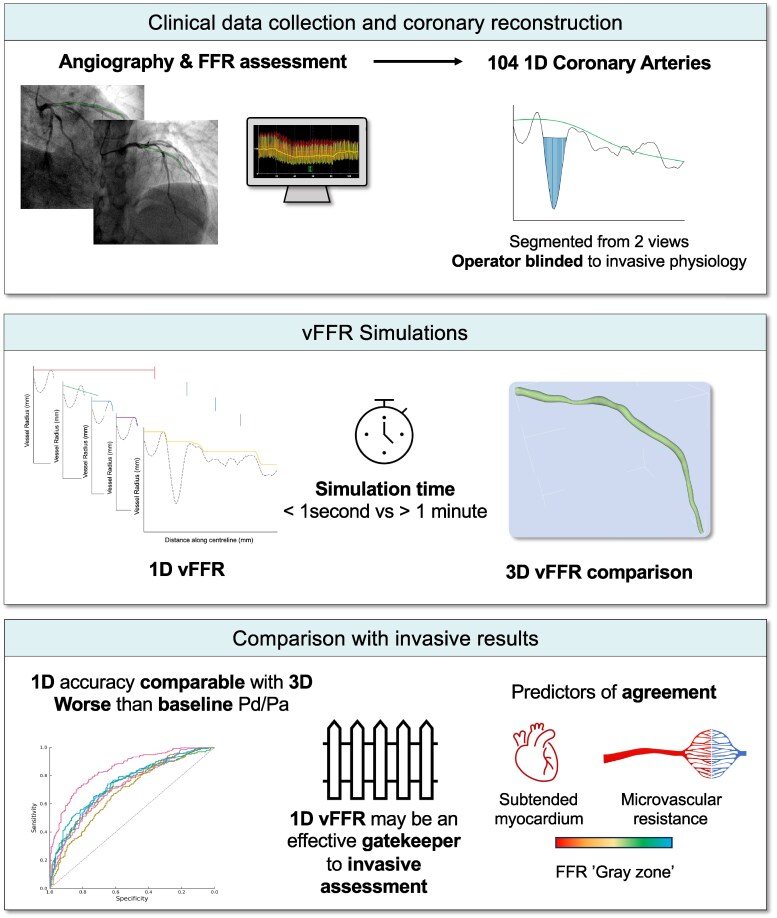

**Time of primary review: 78 days**

**See the editorial comment for this article ‘One-dimensional virtual fractional flow reserve: promise, pragmatism, and next steps’, by R.A. Sykes**  ***et al*****., https://doi.org/10.1093/cvr/cvaf193.**

## 1. Introduction

Functional assessment of coronary artery disease (CAD) with invasive fractional flow reserve (FFR) is the current gold standard, recommended by both European^1^ and American^2^ guidelines (class 1A). FFR-guided revascularization with percutaneous coronary intervention (PCI) improves patient outcomes vs. angiographically guided PCI.^3^ However, widespread adoption of FFR has been held back by increased procedural cost, time and side effects of hyperaemia induction.

Physics-based computational models of virtual FFR (vFFR) offer a non-invasive alternative, removing the need for pressure wires or pharmacologically induced hyperaemia. The first vFFR technique was described over ten years ago^4^ and several models are now commercially available (QFR [Medis Medical Imaging Systems], CAAS vFFR [Pie Medical Imaging], caFFR [Rainmed Ltd], Murray law–based quantitative flow ratio µQFR, [Pulse Medical Imaging Technology], and FFRangio [CathWorks Ltd]). Whilst subtle differences may exist, vFFR workflows designed for use in the cardiac catheterization laboratory typically require reconstruction of an *in-silico* coronary artery from planar angiography. A mathematical formulation of the governing fluid dynamics within the geometry is solved to compute an expected pressure loss under hyperaemic conditions. Simulation times for these online calculations are an important consideration. However, the current role of vFFR technologies for guiding clinical decisions is equivocal. Whilst the latest European guidelines have endorsed their use (class 1B recommendation),^1^ emerging clinical trial data indicate vFFR-guided treatment does not achieve non-inferiority for hard end-points (death, myocardial infarction, and unplanned revascularization) vs. FFR.^5^ Agreement, or the lack thereof, between vFFR with invasive FFR underpins these differences. Recent data suggest vendor-reported accuracy of commercially available models may be an overestimate in some patient groups.^6^ Consequently, challenges persist in optimizing vFFR workflows, achieving consistent FFR agreement across diverse patient cohorts, and defining optimal scope within clinical practice.

Several developments of vFFR have aimed to improve accuracy and translation into real-time use within the catheterization laboratory.^7^ By reducing the coronary geometries and Navier–Stokes equations to a 1-dimensional (1D) representation,^8,9^ simulation times are shortened by orders of magnitude. Additionally, vessel taper, resulting from bifurcations, has been used to derive side branch flow, which is included in simulations by modelling the vessel wall as a boundary through which virtual fluid can ‘leak’.^10^ Models utilizing leakage combine taper of the reconstructed vessel and vascular morphometric scaling laws^11,12^ to determine the magnitude of side branch flow.^13^ One model of vFFR, which incorporates morphometric scaling law-derived leakage, is commercially available (µQFR, [Pulse Medical Imaging Technology]).^14^ Several explicit leakage functions have been described, which may distribute side branch flow homogeneously across the entire vessel^15^ or localize leakage to bifurcations.^16^ Recently, local pressure gradients have also been incorporated into side branch flow computation^17^ and the 1D description of flow has been updated to better account for taper and leakage.^18^ However, a direct comparison of these updated models in a matched cohort has not been published.

The primary aim of this work was to perform the first invasive validation of several novel 1D models of vFFR which incorporate side branch flow in a matched cohort of patients with intermediate CAD. To aid clinical interpretation, we also compared 1D vFFR with 3-dimensional (3D) simulations, visual assessment and invasive pressure readings taken under resting conditions. Secondary aims included evaluation of predictors of vFFR diagnostic accuracy and quantification of agreement with invasive FFR in angiographically healthy arteries.

## 2. Methods

### 2.1 Patient recruitment and clinical data collection

Data for this retrospective cohort study were sourced from the University of Sheffield coronary physiology repository. This included data from adult patients undergoing cardiac catheterization and invasive FFR assessment of intermediate diameter coronary stenoses, for evaluation of suspected chronic coronary syndromes, which have been used for previous computational modelling studies.^19^ Exclusion criteria included ostial CAD, ST-segment elevation myocardial infarction within 60 days, contraindication to adenosine or contrast media, previous coronary artery bypass surgery, chronic total occlusion, severe valvular disease or inability to consent. Coronary angiograms were acquired following standard clinical protocols, with operators encouraged to optimize image acquisition for computational reconstruction. Disease pattern (focal vs. diffuse) and diameter stenosis (considered significant if >50%) were both visually assessed against the moving angiogram by a clinician blinded to invasive physiology. Invasive pressure measurements were taken using a PressureWire X (Abbott Laboratories) in arteries of interest, under both baseline and hyperaemic conditions.^20^ Under baseline conditions, the resting full cycle ratio (Pd/Pa) across the entire cardiac cycle was recorded and considered significant if ≤0.90. Hyperaemia was induced with an intravenous infusion of adenosine (140 µg/kg/min). A second cohort of angiographically healthy vessels (<20% visually assessed diameter stenosis), taken from patients undergoing continuous infusion thermodilution (Rayflow) assessment for evaluation of suspected microvascular dysfunction at the Catharina Hospital, Eindhoven NL, was also included.^21^ For all cases, data collection for research purposes was approved by the relevant Regional Ethics Committees (16/NW/0897, 08/H1308/193, MEC-U), compliant with the Declaration of Helsinki and all patients gave written informed consent prior to inclusion. Pseudonymized angiography (DICOM) and physiological (pressure) data were exported to the University of Sheffield for offline computational processing and statistical analysis.

### 2.2 Coronary reconstruction

A full description of the coronary artery reconstruction protocol has previously been published.^4^ Two angiographic projections, acquired ≥ 30° apart, clearly displaying the vessel and lesion of interest in end-diastole with minimal foreshortening or vessel overlap were selected. Table movement artefact was corrected. The arterial reconstruction inlet and outlet were manually selected to correspond to the locations of aortic pressure (Pa) and distal coronary pressure (Pd) respectively, which were both recorded during invasive FFR assessment. The vessel centreline and borders were traced semi-automatically, with manual correction if required. Finally, a rigid, locally axisymmetric 3D reconstruction was generated using an epipolar line method. Reconstructions were performed blinded to invasive physiology and every reconstruction was checked for anatomical accuracy by a panel of three cardiologists, blinded to the invasive FFR, against the original angiogram. To create 1D geometries for validation simulations, radii were sampled at 200 points along the arterial reconstruction centreline.

### 2.3 vFFR simulations

Computation of 1D vFFR was performed by sequentially calculating pressure loss at each of the 200 discretised radii for every coronary geometry. In healthy sections of vessel, one of five different 1D models of steady flow, derived from the Navier–Stokes equations was used. The simplest model did not allow for side branch leakage^22^ whilst the remaining four included distinct side branch flow models as follows:

1. Homogenous leakage, determined from total vessel taper, with side branch flow distributed equally across the entire reconstruction.^15^
2. Localized leakage, determined from local vessel taper. The magnitude of side branch flow was proportional to taper, which aims to focus side branch flow to bifurcations. Regions with downstream radius recovery did not leak.^16^
3. Conductance leakage, determined from local vessel taper and pressure. This model lowered the magnitude of leakage for equivalent regions of taper distal to stenosis-induced pressure loss. The vessel pressure field was initialized from a localized leakage simulation.^17^
4. Porosity leakage, a novel leakage function, sensitive to local taper and pressure, which used the Darcy–Forchheimer equation to assign vessel porosity. This eliminates the need for assumptions of localized anatomical leakage (see the appendix and supplementary material).

**Figure 1:**
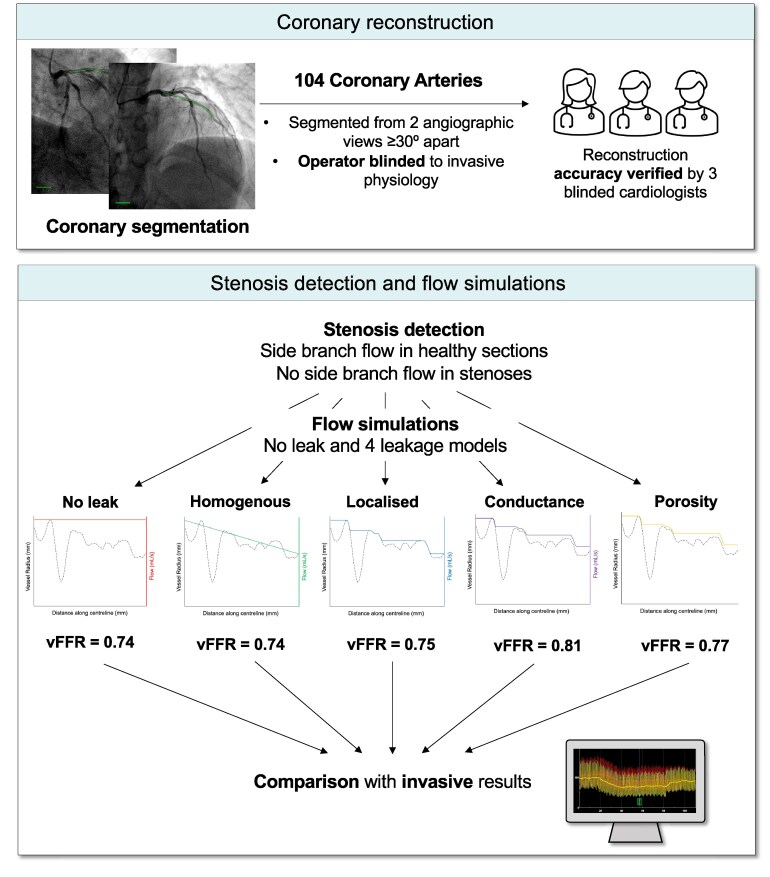
Schematic workflow for arterial reconstruction and flow simulations in the current study. Scale bar in the angiogram lower left-hand corner denotes 10 mm length.

Taper of the reconstructed vessel was used to estimate the size of unresolved side branches using the Huo-Kassab law of vascular scaling,^12^ which is supported by observational data from coronary arteries^23^:


$$\mathrm{Inlet}\;\mathrm{flow}=\mathrm{Outlet}\;\mathrm{flow}\;{\left(\frac{R_{\mathrm{in}}}{R_{\mathrm{out}}}\right)}^{\frac{7}{3}}$$

where *R*_in_ and *R*_out_ denote vessel inlet and outlet radius respectively. In sections of stenosis, which invoke significant radial flow in the *in-vivo* artery, assumptions of the healthy 1D models are violated. To identify stenosed regions, a Fourier filtration method^17^ was applied to generate healthy vessel representations, which were compared with reconstruction area (see supplementary  *S2* for vessel filtration code in MATLAB). Stenosed regions were defined as points where reconstructed vessel area decreased below 80% of estimated healthy area.^22,24^ In these circumstances, an empirically derived, lumped sub-model relating the dimensions of the stenosis to the pressure drop was utilized.^25,26^ No leak occurred in stenosed sections of vessels. Therefore, for each coronary artery, five total 1D vFFR results were obtained. In healthy (unstenosed) sections of vessel, the model of flow varied between no leak, or one of the four leakage models enumerated above, and in stenosed sections the lumped sub-model was used for all simulations (see *Figure 1* for summary of flow models).

Boundary conditions for flow simulations included patient-specific Pa, measured during coronary catheterization, and inlet flow (mL/s). The latter was optimized against microvascular resistance (MVR) within the range of 50–450 mL/min.^21^ MVR was predicted by a machine learning non-linear autoregressive moving average with exogenous inputs (NARMAX) model, which has been previously validated in coronary arteries.^27^ The NARMAX model was personalized according to vessel type, vessel dimensions, lesion-specific myocardial jeopardy score and presence of comorbid hypercholesterolaemia or chronic lung disease. This approach therefore aimed to give patient- and artery-specific boundary conditions. Simulations for the primary outcome used standard parameters for blood viscosity μ = 0.0035 Pa.s and density ρ = 1050 kg/m^3^. To evaluate the effect of patient-specific viscosity, simulations were also performed personalized to measured haematocrit^28^:


Personalisedviscosity(Pa.s)=0.0014+0.0035Haematocrit

where 0.0014 represents an assumed viscosity of plasma and the viscous contribution of erythrocytes was personalized to haematocrit.

To give insight into the effect of reducing coronary geometries to a 1D representation, vFFR was also computed with a 3D model (VIRTUheart),^4^ which resolves the Navier–Stokes and continuity equations under steady state conditions. This therefore makes fewer assumptions than 1D vFFR models. All 3D simulations did not include side branch leakage. Input parameters for 3D simulations included the coronary reconstruction, patient-specific aortic pressure and NARMAX MVR.

### 2.4 Statistical analysis

An *a priori* power analysis was conducted, using G*Power version 3.1, to determine the required sample size for a two-tailed dependent samples *t*-test. We chose to power our study based on the expected difference between localized and conductance leakage functions using pilot data comparing vFFR in matched, idealized cases.^17^ We calculated an expected effect size of 0.29 (considered small using Cohen’s criteria). With a risk of type I error of 5 and 80% power, a minimum 96 cases were required. We expected a low level of attrition from simulation failures, so aimed to include at least 100 cases in the final analysis.

Categorical variables are presented as frequency (percentage). Continuous variables are presented as mean (standard deviation) or median [inter-quartile range] for normally distributed and skewed data respectively. Normality of data distribution was assessed using the Shapiro–Wilk test. Mean values were compared using *t*-tests, the Mann–Whitney *U* test and Kruskal–Wallis test as appropriate. Categorical data were compared with the chi-square (χ²) test. Pearson’s correlation coefficient (*r*) and Spearman’s Rho were used to quantify correlation as appropriate. Agreement was assessed with Bland Altman plots and Passing and Bablok regression. Where data did not meet the parametric assumptions for the original Bland Altman method,^29^ we derived median bias and limits of agreement using quantile regression at the 50th, 2.5^th^, and 97.5th centiles.^30^ The diagnostic performance of each model of flow was also quantified with total diagnostic accuracy, sensitivity, specificity, positive predictive value (PPV), negative predictive value (NPV), with associated confidence intervals derived using the Clopper–Pearson Exact method, and receiver operator characteristic (ROC) curves with calculated area under the curve (AUC). As secondary analyses, predictors of total diagnostic performance, false positive and false negative results were assessed with a logistic regression model returning a log-odds and significance value. To evaluate the impact of Fourier vessel filtration on vFFR accuracy, a subset of cases with optimal healthy radius fitting were manually selected by a clinician (DJT) and a control systems engineer (XX), both blinded to invasive results. We also report diagnostic accuracy of 3D vFFR, baseline Pd/Pa and visual assessment to aid clinical contextualization. Analyses were performed using the Julia language with the Statistics.jl and JUMP.jl packages to perform the statistical tests and implement the logistic regression model respectively.

## 3. Results

### 3.1 Study population

One-hundred and four cases were suitable for inclusion. All were included in the analysis. The 104 included cases contained data from 85 patients, of which 65 (76%) were male and mean age was 63.6 (±9.5) years. Most lesions were in the LAD artery 60 (58%), with 22 (21%) in the RCA and 13 (13%) in the LCx artery (*Table 1*). Median visually assessed diameter stenosis was 60% [50–70%]. Mean reconstruction inlet and outlet diameters were 2.7 mm (± 0.5) and 1.9 mm (± 0.4) respectively, indicating an approximately equal distribution between outlet flow and side branch wall leakage in most cases (see supplementary  *S3* for angiogram and radius data for every case).

**Table 1:** Patient demographics and lesion characteristics of included cases used for the primary outcome

Patient Demographics (*n* = 85)	
Age, y	63.6 ± 9.5
Male	65 (76%)
White Caucasian	77 (95%)
Current or ex-smoker	54 (64%)
Haematocrit (%)	0.42 ± 0.04
Comorbidities	
Hypertension	57 (67%)
Diabetes Mellitus	20 (24%)
Hypercholesterolaemia	64 (75%)
Previous myocardial infarction	15 (18%)
Valvular heart disease	3 (4%)
Moderate-severe left ventricular systolic dysfunction	16 (21%)
Chronic lung disease	10 (21%)
Vessel characteristics (*n* = 104)	
LAD	60 (58%)
LCx	13 (13%)
RCA	22 (21%)
Dx	3 (3%)
OM	4 (3%)
LMS	5 (3%)
Visual diameter stenosis	60% [50%—70%]
Lesion-specific myocardial jeopardy	0.30 ± 0.13
FFR	0.81 [0.73–0.88]
Number of lesions with FFR ≤0.80	46
Focal disease	59 (57%)
Diffuse disease	45 (43%)

LAD, left anterior descending artery; LCx, left circumflex artery; RCA, right coronary artery; Dx, diagonal branch; OM, obtuse marginal branch; LMS, left main stem; FFR, fractional flow reserve.

### 3.2 Invasive results

Median FFR was 0.81 [0.73–0.88]. Pressure data under baseline conditions were available for 101 cases, in which median Pd/Pa was 0.93 [0.88–0.96]. Supplementary  *S4* shows the distribution of FFR and Pd/Pa readings for all included cases. Forty-six (44%) cases had FFR ≤ 0.80 and 30 (29%) cases were in the FFR ‘gray zone’ of 0.75–0.85 (see supplementary material).

### 3.3 Agreement and diagnostic accuracy of 1D vFFR

**Figure 2:**
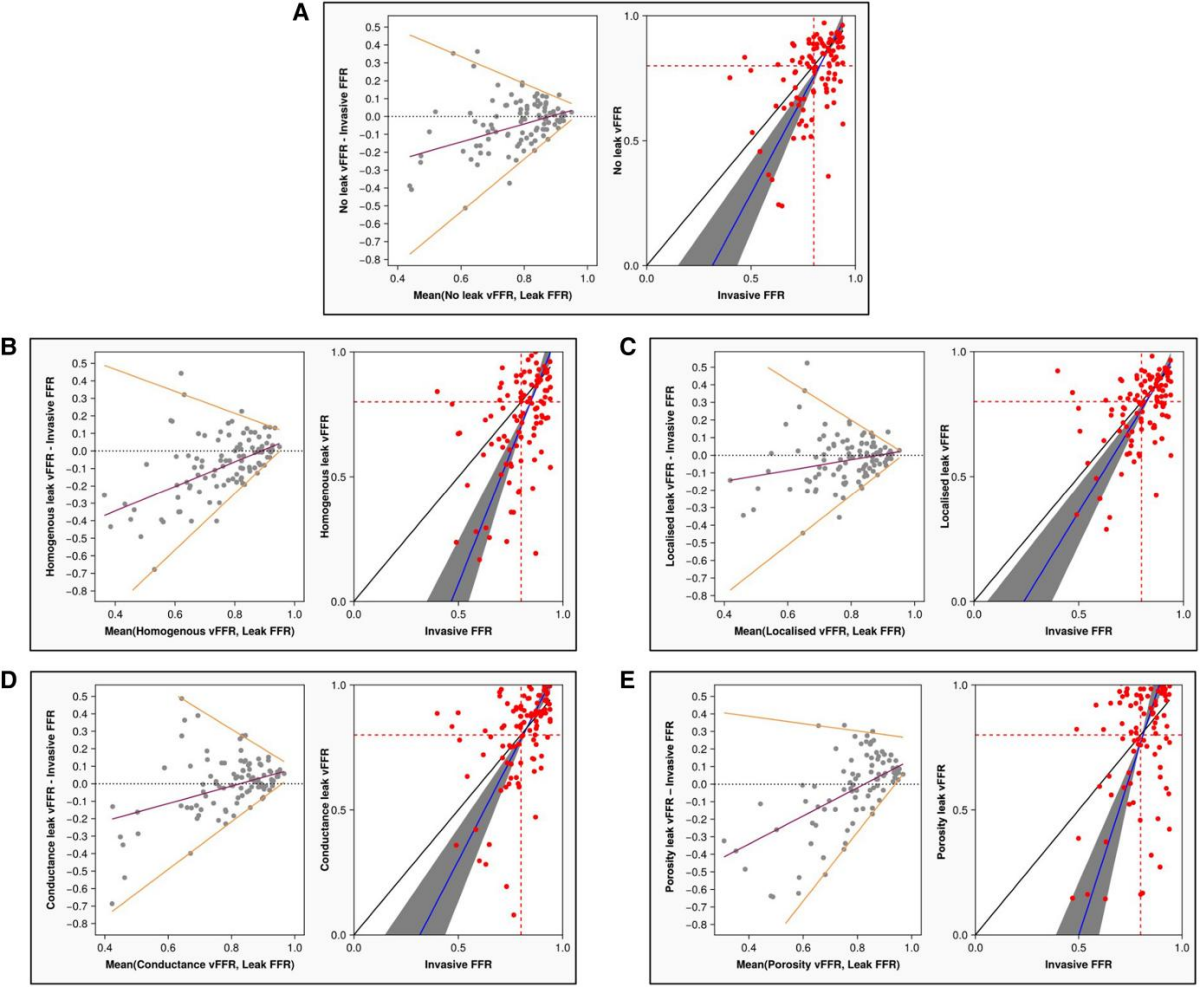
Bland Altman plot and Passing and Bablok regression for all 1D models of flow. (*A*) No leak model (101 biological observations); (*B*) homogenous leak model (102 biological observations); (*C*) localized leak model (102 biological observations); (*D*) conductance leak model (99 biological observations); (*E*) Porosity leak model (88 biological observations). All observations were sourced from the original 104 recruited cases. For Bland Altman plots, median bias, 2.5th and 97.5th limits of agreement were determined from quantile regression fitted across the entire measurement range.

**Figure 3:**
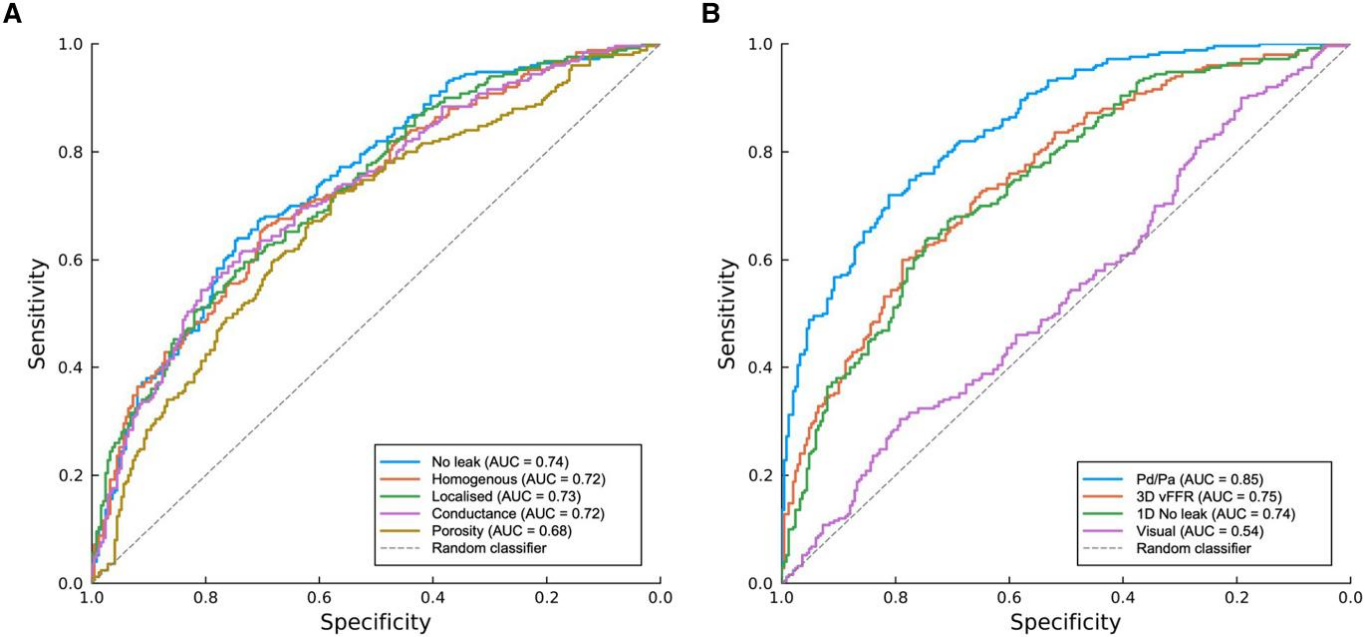
ROC curves. (*A*) Displays results for all five 1D models of flow, number of biological observations for the no leak, homogenous, anatomical, conductance and porosity models 101, 102, 102, 99, and 88, respectively. (*B*) Displays results of the 1D no leak model against 3D vFFR (101 observations), baseline Pd/Pa (103 observations) and visual assessment (104 observations) to aid comparison. All observations were sourced from the original 104 recruited cases. AUC values derived from ROC analysis.

Of the five 1D models (no leak, homogenous, localized, conductance, and porosity), vFFR was successfully computed in 101 (97%), 102 (98%), 102 (98%), 99 (95%), and 88 (85%) cases respectively. Failure rate of the porosity model was significantly higher than all other models of flow (χ^2^ = 26.6, *P* < 0.0001), which was attributed to solutions giving a negative pressure value in all excluded cases. A distribution of vFFR results for each model of flow is shown in the supplementary material. Correlation with invasive FFR was moderate for no leak (*r* = 0.48, *P* < 0.0001), homogenous leak (*r* = 0.45, *P* < 0.0001), localized leak (*r* = 0.44, *P* < 0.0001), and conductance leak (*r* = 0.42, *P* < 0.0001), whereas this was mild with porous leak (*r* = 0.33, *P* = 0.0018). For all 1D models, there was a trend of poorer agreement with FFR with progressively worse epicardial disease (i.e., lower FFR values). At the diagnostic threshold of 0.80, all 1D models underestimated invasive FFR and the closest agreement was observed with the localized leakage model (median bias −0.03, 95% LOA −0.23 to 0.20) (see *Figure 2*, *Table 2* and supplementary material). There was a significant difference in agreement with invasive FFR between the different models of flow (*H* = 13.8, *P* = 0.008), of which the effect size was small (*η*^2^ = 0.02). Using Dunns comparison method, these differences were between the homogenous and conductance models and the homogenous and porosity models. Diagnostic accuracy was highest for the conductance model (68.7% (59.0%—78.0%)) and lowest for the porosity model (60.2% (49.8%—71.0%)), but this difference did not meet statistical significance (see *Table 2* for full accuracy, sensitivity, specificity, PPV, and NPV results). ROC curves for each model of flow in predicting FFR ≤0.80 are shown in *Figure 3*, AUC values varied between 0.74–0.68 and were highest for the no leak model and lowest for the porosity model. For all 1D vFFR models, diagnostic accuracy reached a nadir at FFR values ranging between 0.75 and 0.85 (see supplementary material  *S8*).

**Table 2:** Agreement and diagnostic performance of vFFR models vs. FFR

	No leak	Homogenous	Localized	Conductance	Porosity
Successful simulations	101 (97.1%)	102 (98.1%)	102 (98.1%)	99 (95.2%)	88 (84.6%)
Median Bias at 0.80	−0.04	−0.06	−0.03	−0.01	−0.02
95% LOA at 0.80	[−0.24, 0.18]	[−0.24, 0.21]	[−0.23, 0.20]	[−0.22, 0.31]	[−0.27,0.30]
True positive	30	35	32	27	22
True negative	37	31	36	41	31
False positive	18	24	19	12	15
False negative	16	12	15	19	20
Accuracy, %	66.3 (56.3–75.5)	64.7 (54.3–73.6)	66.7 (56.6–75.7)	68.7 (59.0–78.0)	60.2 (49.8–71.0)
Sensitivity, %	65.2 (49.8–78.7)	74.5 (59.7–86.1)	68.1 (52.9–80.9)	58.7 (43.2–73.0)	52.4 (36.4–68.0)
Specificity, %	67.3 (53.3–79.3)	56.4 (42.3–69.7)	65.5 (51.4–77.8)	77.4 (63.8–87.7)	67.4 (52.0–80.5)
Positive predictive value, %	62.5 (50.6–70.9)	59.3 (48.9–65.6)	62.7 (50.8–70.2)	69.2 (54.1–78.1)	59.5 (43.3–67.8)
Negative predictive value, %	69.8 (61.2–79.1)	72.1 (61.9–82.7)	70.6 (62.1–80.4)	68.3 (61.9–77.5)	60.8 (55.1–72.2)
AUC	0.74	0.72	0.73	0.72	0.68

Successful simulations denotes the number of biological observations for each 1D model of flow (zero technical replicates for every model). Median bias, 2.5th and 97.5th limits of agreement determined from quantile regression across the entire cohort and are reported at mean FFR/vFFR of 0.80. Confidence intervals for diagnostic accuracy, sensitivity, specificity, positive predictive value, and negative predictive value derived using the Clopper–Pearson Exact method.

### 3.4 Comparison with 3D vFFR, visual assessment and Pd/Pa

3D vFFR was successfully computed in 101 cases. Correlation with invasive FFR was mild (r = 0.38, *P* < 0.0001). At the diagnostic threshold of 0.80, 3D vFFR overestimated FFR by 0.03 (95% LOA −0.17 to 0.25). On a case-by-case basis, there was a significant difference in agreement between 3D and 1D vFFR models (*H* = 34.0, *P* < 0.0001), of which the effect size was small (η2 = 0.05). However, vs. 1D models, this did not translate into a significant difference in overall agreement across the entire cohort. Using Dunns comparison method, these differences in agreement were with the no leak, homogenous and localized 1D models. Overall diagnostic accuracy of 3D vFFR was (62.0%, 95% CI 51.8% to 71.6%) and AUC was 0.75, both of which were comparable with 1D vFFR results. Mean visual diameter stenosis was 59.2% ± 15.0% and correlation between visual assessment with FFR was moderate (*r* = −0.57, *P* < 0.0001). Eighty-eight (85%) lesions were classified as visually significant, diagnostic accuracy was 53.5% (95% CI 43.4 to 63.4%) and AUC was 0.54. Median Pd/Pa was 0.93 [0.88–0.96] and correlation between the two pressure readings was strong (*r* = 0.87, *P* < 0.0001). Pd/Pa classified 35 (35%) lesions as significant, diagnostic accuracy was 82.9% (95% CI 74.1 to 89.6%) and AUC was 0.85 (*Figure 3* and supplementary material  *S13*–S15).

### 3.5 Predictors of FFR concordance

Logistic regression consistently identified higher total myocardial jeopardy as a predictor of concordance between 1D vFFR and FFR, the effect of which was most pronounced with the homogenous model of flow (log odds 3.30, 95%CI 2.00 to 4.61). Greater visually assessed lesion diameter stenosis, LAD arteries and RCAs were also positively associated with concordant classification for several 1D models. Discordant classification was most strongly associated with larger reconstruction inlet diameter followed by patients with previous MI, previous PCI and female sex (*Table 3*). Supplementary material  *S9* shows logistic regression results for predictors of false positive and false negative 1D vFFR results. Patient-specific haematocrit was available for all recruited patients. Personalization of fluid viscosity to haematocrit did not significantly improve overall agreement or diagnostic accuracy, for any 1D model of flow (see supplementary  *S10*). Similarly, overall agreement and diagnostic accuracy did not significantly different between focal and diffusely disease cases (full results supplementary  *S11*). A subgroup of 36 cases with optimal healthy vessel radius estimation from Fourier filtration was identified. In this subgroup, neither agreement with invasive FFR or diagnostic performance improved vs. the entire cohort of 104 cases (see supplementary  *S12*).

**Table 3:** Predictors of FFR vFFR concordance

	No-Leak		Homogenous		Anatomical		Conductance		Porosity	
Parameter	Log-odds (95% CI)	*P*-value	Log-odds (95% CI)	*P*-value	Log-odds (95% CI)	*P*-value	Log-odds (95% CI)	*P*-value	Log-odds (95% CI)	*P*-value
Female	−1.27 (−2.47, −0.06)	0.039	−1.39 (−2.57, −0.21)	0.210	−0.71 (−1.88, 0.47)	0.239	−1.19 (−2.43, 0.05)	0.061	−0.38 (−1.62, 0.85)	0.543
Age	−0.02 (−0.08, 0.05)	0.591	0.01 (−0.05, 0.07)	0.787	−0.01 (−0.08, 0.06)	0.762	0.03 (−0.05, 0.11)	0.429	0.05 (−0.02, 0.11)	0.179
Current smoker	0.44 (−0.70, 1.59)	0.449	0.50 (−0.63, 1.63)	0.387	0.18 (−0.97, 1.33)	0.020	0.80 (−0.38, 1.98)	0.183	0.60 (−0.60, 1.80)	0.326
Ex-smoker	−0.64 (−1.72, 0.45)	0.250	−1.46 (−2.55, −0.36)	0.900	−0.68 (−1.77, 0.41)	0.223	−1.07 (−2.22, 0.08)	0.068	0.00 (−1.12, 1.12)	1.000
Diabetic	−0.05 (−1.23, 1.12)	0.931	−0.73 (−1.91, 0.46)	0.228	−0.55 (−1.73, 0.63)	0.361	0.24 (−0.96, 1.44)	0.698	0.01 (−1.18, 1.19)	0.993
Hypertensive	−0.12 (−1.38, 1.13)	0.848	−0.83 (−2.08, 0.43)	0.197	−0.08 (−1.35, 1.18)	0.897	−0.27 (−1.55, 1.00)	0.674	−0.37 (−1.62, 0.89)	0.564
Dyslipidaemia	−0.44 (−1.69, 0.81)	0.491	−0.34 (−1.60, 0.92)	0.598	−0.41 (−1.67, 0.84)	0.519	−0.80 (−2.07, 0.46)	0.214	−0.03 (−1.28, 1.23)	0.968
Previous MI	−1.38 (−2.69, −0.06)	0.040	−1.55 (−2.79, −0.30)	0.150	−1.36 (−2.68, −0.05)	0.420	−1.26 (−2.58, 0.07)	0.630	−0.38 (−1.60, 0.84)	0.542
Mild LVSD	−1.31 (−2.53, −0.09)	0.036	−1.77 (−2.97, −0.56)	0.400	−1.30 (−2.52, −0.09)	0.350	−1.51 (−2.82, −0.20)	0.240	0.39 (−0.80, 1.59)	0.520
Moderate LVSD	0.45 (−0.79, 1.69)	0.479	1.42 (0.20, 2.64)	0.220	0.46 (−0.78, 1.70)	0.467	−0.82 (−2.16, 0.52)	0.228	0.32 (−0.96, 1.60)	0.623
Severe LVSD	−0.20 (−1.57, 1.18)	0.781	−0.54 (−1.91, 0.83)	0.438	−0.27 (−1.64, 1.10)	0.700	−0.41 (−1.77, 0.96)	0.557	0.19 (−1.11, 1.49)	0.777
Previous PCI	−0.94 (−2.20, 0.31)	0.142	−1.53 (−2.79, −0.26)	0.180	−0.79 (−2.04, 0.46)	0.214	−1.38 (−2.66, −0.09)	0.360	0.09 (−1.19, 1.37)	0.893
Artery LAD	0.65 (−0.43, 1.74)	0.240	1.18 (0.09, 2.27)	0.033	0.49 (−0.60, 1.58)	0.376	0.65 (−0.49, 1.78)	0.264	0.21 (−1.30, 0.89)	0.710
Artery LCx	0.02 (−1.19, 1.23)	0.973	−0.53 (−1.75, 0.69)	0.395	−0.23 (−1.43, 0.98)	0.713	−0.21 (−1.45, 1.03)	0.741	0.33 (−0.85, 1.52)	0.579
Artery RCA	0.96 (−0.19, 2.11)	0.010	2.27 (1.10, 3.43)	< 0.001	1.21 (0.06, 2.36)	0.040	0.25 (−0.95, 1.45)	0.001	−0.14 (−1.28, 1.00)	0.010
Vessel inlet diameter	−1.65 (−2.71, −0.58)	0.002	−1.80 (−2.86, −0.74)	0.001	−1.49 (−2.56, −0.42)	0.600	−0.83 (−1.95, 0.30)	0.151	−0.71 (−1.73, 0.30)	0.169
Vessel outlet diameter	−0.23 (−1.35, 0.88)	0.681	−0.74 (−1.85, 0.37)	0.192	−0.70 (−1.82, 0.43)	0.223	−0.80 (−1.98, 0.38)	0.185	0.82 (−0.34, 1.98)	0.168
Lesion diameter stenosis (%)	1.24 (−0.10, 2.58)	0.070	1.89 (0.55, 3.24)	0.006	1.30 (−0.04, 2.64)	0.058	1.40 (0.05, 2.75)	0.042	0.69 (−0.66, 2.04)	0.315
Total myocardial jeopardy	1.52 (0.23, 2.82)	0.020	3.30 (2.00, 4.61)	< 0.001	2.39 (1.09, 3.68)	< 0.001	1.37 (0.06, 2.68)	0.040	−0.79 (−2.10, 0.52)	0.003
Lesion specific jeopardy	−0.17 (−1.55, 1.21)	0.030	0.55 (−0.83, 1.93)	0.433	0.10 (−1.28, 1.48)	0.889	−0.09 (−1.47, 1.29)	0.898	−0.08 (−1.46, 1.31)	0.914

Results for the no leak, homogenous, anatomical, conductance, and porosity models were derived from 101, 102, 102, 99, and 88 biological observations, respectively (all sourced from the original 104 recruited cases). All *P*-values were derived from binary logistic regression fitting within the JUMP.jl package. MI, myocardial infarction; LVSD, left ventricular systolic dysfunction, LAD, left anterior descending artery; LCx, left circumflex artery; RCA, right coronary artery.

### 3.6 Agreement in minimally stenosed arteries

Agreement was assessed in 20 angiographically healthy arteries (see supplementary  *S16*). In this cohort, median visually assessed diameter stenosis was 5% [0–15%], median FFR was 0.89 [0.84–0.96] and zero vessels produced an FFR ≤ 0.80. Median invasively assessed MVR was 361 Woods Units (WU) [333–405 WU]. Compared with stenosed vessels, for all 1D models of flow, agreement with invasive FFR significantly improved and diagnostic accuracy approached 100%. However, there was little to no evidence of a relationship between invasive MVR and agreement between 1D vFFR accuracy in this cohort (see supplementary  *S17* for full demographics and results).

## 4. Discussion

In this study, we compared agreement and diagnostic accuracy of five 1D models of vFFR with invasive physiology. The main findings are:

1. Simulation of side branch flow was moderately influential on overall agreement between 1D vFFR and invasive FFR. Agreement was strongest for the model which localized side branch flow to bifurcations, but this did not translate into a significant improvement in diagnostic accuracy.
2. Diagnostic performance of several 1D vFFR models were superior to visual assessment and comparable to 3D vFFR simulations, but not invasively assessed Pd/Pa.
3. Regardless of the 1D model used, the ability of vFFR to classify FFR-positive lesions was not sufficient to suggest they may replace wire-based assessment around the ‘grey zone’.

### 4.1 Current accuracy and future development of 1D vFFR models

Computational models of vFFR, derived from angiography, were first described in 2013.^4^ Four years later, they were first licensed for clinical use (CAAS vFFR [Pie Medical Imaging]) and in recent months, they received a class 1B recommendation in European guidelines.^1^ Compared with some clinically licensed solutions, 1D vFFR significantly reduces simulation time and, in our cohort, showed comparable agreement with 3D simulations. Accuracy of these physics-based models is dependent upon their ability to capture haemodynamics of the *in-vivo* coronary artery. In healthy sections of vessel, this was represented by 1D models of steady flow, derived from the Navier–Stokes equations. Most models of healthy flow included an account of side branch flow with leakage. In stenosed regions, where significant radial flow may occur, an empirically derived lumped sub-model was used. Accurately capturing patient-specific haemodynamics with these equation systems is a complex problem; epicardial coronary arteries integrate into a complex bifurcating tree, which is in constant motion and carries pulsatile flow. Further, stenosis morphology,^31^ microvascular pathology,^21,32^ valvular pathology^33^ and cardiac rhythm^34^ are just some pathological states, which influence coronary flow. These pose significant challenges for vFFR workflows, which must balance increasing model complexity against consequent heightened input uncertainty and result sensitivity.^35^ For 1D vFFR, the effect of including side branch flow was modest and whilst a significant difference in agreement was observed between some leakage models, this did not translate to significant improvements in diagnostic accuracy. This agrees with a previous clinical validation study,^15^ but conflicts with sensitivity analyses suggesting the inclusion of side branch flow may influence vFFR results around the FFR ‘grey zone’.^36^ This suggests that although influential in some cases, representation of side branch flow is of secondary importance for overall 1D vFFR model accuracy.

The comparable agreement between 1D and 3D models underscores the importance of MVR for vFFR accuracy,^37^ suggesting methods for optimizing MVR predictions will be key for future improvements. This observation is supported by our finding of previous myocardial infarction (MI) and patient sex as strong predictors of 1D vFFR discordance with FFR; despite MVR being higher post-MI and in females,^38^ the NARMAX model is not currently parametrised by either of these variables. Total myocardial jeopardy was also strongly associated with diagnostic accuracy, this accords with other work showing subtended myocardial mass is influential for vFFR accuracy.^6^ Vessel reconstruction error is also likely to have worsened concordance between 1D vFFR and FFR and is supported by our findings of percentage stenosis and inlet vessel diameter both being strong predictors of correct lesion classification. The latter may be underpinned by the worsening discrepancy between quantitative coronary angiography and true lumen size in larger, proximal vessels.^39^ Contrastingly, personalization of fluid viscosity to patient-specific haematocrit was not influential on vFFR agreement or diagnostic accuracy. This supports previous sensitivity analyses, suggesting patient-specific tuning of fluid rheology is unlikely to confer a significant improvement.^40^

The improved agreement and diagnostic accuracy in angiographically healthy vessels were expected; in vessels with minimal disease, pressure gradients are small so, invasive and virtual FFR values tend to converge.^41^ The influence of MVR in this cohort appears to be diminished, with no significant relationship between vFFR/FFR agreement and invasive microvascular physiology. This finding suggests that whilst microvascular dysfunction is known to influence trans-lesional physiology,^42,43^ the influence for vFFR in angiographically healthy vessels is likely to be less clinically significant vs. increasingly stenosed vessels.

### 4.2 Future development of 1D vFFR models

Further work will complement ongoing clinical research. For all 1D models, lesion concordance was significantly altered in RCA vessels. Given these are typically distinguished from LAD and LCx vessels by their curvature, anatomical factors may explain this discrepancy. Specifically, significant recirculation regions may be present, which would violate assumptions of 1D flow. Pulsatile 3D CFD simulations may assess if certain cases violate these assumptions in curved coronary arteries and at what degree of curvature this becomes clinically relevant. Detection of stenosed regions by the 1D model also remains an empirical process compared to the well-established numerical methods for 3D simulations. This is dependent upon accurate healthy vessel estimation and frequently utilizes the Gaussian kernel filtering method of Shahzad *et al*.^44^ Whist this is regularly utilised for vFFR investigations,^22,24,45^ the method contains three empirical parameters referring to healthy vessel variability and kernel length which are not conserved between most studies. The impact of changing the coefficients used for these hyper-parameters is poorly understood. In this study, we utilized a method grounded in Fourier filtration^17,36^ which was dependent upon a single parameter controlling the quality of filtration. Our results suggested this method of healthy vessel filtration does not currently constitute a dominant source of error for 1D models. However, the translational effects of various stenosis detection thresholds and stenosis model hyper-parameter coefficient values remain largely unknown and may become significant with other model developments. The 1D model is dependent upon the ratio between actual and healthy vessel area when identifying areas of stenosis. In the current study, we used a ratio of 0.80 as the threshold for stenosis identification. However, significant variation is reported relating to what ratio optimally differentiates regions with significant radial flow.

### 4.3 Wider context and clinical implications

**Figure 4:**
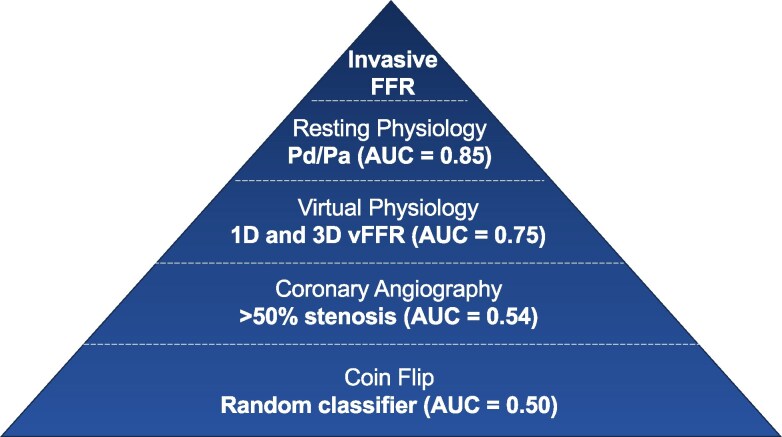
Pyramid of diagnostic accuracy from data in the current study. Angiography discriminator derived from 104 observations. Virtual physiology discriminator derived from 101 observations for both 1D and 3D models. Resting Pd/Pa discriminator derived from 103 observations. All observations were sourced from the original 104 recruited cases. AUC values derived from ROC analysis.

The optimal scope of clinical application for vFFR tools is an active area of research. The current study presents accuracy of novel 1D models, which is an important consideration when defining scope of appropriate clinical use. Rates of concordance between FFR and Pd/Pa in the current study were comparable to historical series,^46,47^ suggesting our results may be applicable to a wide range of patients undergoing vFFR assessment. Results were also comparable with a recent large validation study of commercialized models, in which vFFR was reported to have AUC values ranging from 0.73–0.75 and Bland Altman LOA exceeding −0.30 to + 0.25.^6^ This suggests the presented 1D models may provide a faster virtual assessment of CAD severity without significant loss of diagnostic accuracy. However, reported diagnostic accuracy was not sufficient to suggest any 1D model currently represents a potential replacement to invasive pressure wire assessment, highlighting a need to define the scope of use for vFFR in current clinical practice. The latest European guidelines give a class 1B recommendation for the use of QFR [Medis Medical Imaging Systems] in evaluating the functional significance of intermediate diameter epicardial stenoses.^1^ This is supported by our finding of 1D vFFR superiority over visual assessment and data from the FAVOR III China trial, which demonstrated an improvement in hard clinical outcomes vs. angiographically guided therapy.^48^ However, recent trial evidence found QFR did not reach non-inferiority margins vs. FFR for similar end-points.^5^ Consequently, whilst vFFR can augment visual analysis, it is unlikely to entirely replace wire-based technologies in the short to medium term. Rather, in catheterization laboratories with access to invasive pressure wires, it may be plausible for vFFR to act as a gatekeeper to invasive assessment (see *Figure 4*). This may aid cardiologists to quickly identify cases requiring FFR assessment, reducing procedural time, cost and radiation exposure.^5^ Taking results of the 1D ‘no leak’ model, vFFR thresholds of ≤0.64 and ≥0.89 were needed to achieve specificity and sensitivity of 85% respectively and employing these thresholds would prevent invasive wire use in 33 (32.7%) cases. With considerable improvement in vFFR accuracy, models may one day replace FFR assessment in some patient cohorts entirely. For this to happen, it is likely accuracy would need to approach that of non-hyperaemic invasive alternatives to FFR, such as Pd/Pa, which correctly identifies FFR significant disease in ∼95% of cases (AUC = 0.86).^46^ These resting indices are supported by excellent evidence suggesting their non-inferiority to FFR in lesion evaluation for similar hard outcomes.^49,50^

### 4.4 Future validation of vFFR

The weight of evidence that further clinical trials of vFFR will provide is underpinned by the absolute agreement between vFFR and measured FFR. Whilst a significant corpus of published evidence is already available,^51^ continued model evolutions and improved understanding of determinants of agreement are important. Case selection, which should be representative of the patient cohort in which knowledge of model accuracy is sought, will be key. Specifically, selection of a high proportion of cases with measured FFR values outside the ‘grey zone’ of 0.75–0.85 may produce misleading diagnostic accuracy results, which trend towards 100% further away from the diagnostic cut off value of 0.80. These concerns may affect several published studies.^52–54^ To improve transparency of our own study, we have included angiographic images used for reconstruction and centreline radius data for every included case.

Translational perspectives In tapering coronary arteries, inclusion of regionalized side branch flow in 1D vFFR computation mildly improves agreement without compromising simulation time. Agreement for ‘grey zone’ FFR lesions was comparable with 3D simulations but remained inferior to non-hyperaemic invasive assessment. Consequently, 1D vFFR may augment visual assessment, allowing rapid identification of select patients in whom haemodynamically significant disease can be ruled in or out. This may reduce invasive wire assessment or induction of hyperaemia in approximately one third of patients. However, accuracy of 1D vFFR is insufficient to suggest it may replace invasive assessment in all patient groups.

## 5. Limitations

We included only data from a single centre, which limits ability to evaluate model accuracy in a wide range of clinical contexts. However, as clinical data were sourced from ‘all comers’ undergoing clinically indicated FFR assessment, the results are likely to be generalizable and previous work has shown accuracy of the 3D workflow to be comparable across a number of sites.^7^ Most included cases were LAD arteries, which typically contain less curvature, and cases with severe ostial disease were excluded. The NARMAX model of personalized MVR may benefit from further training cases, which may consider a wider selection of demographic and comorbidity data than the initial training set. All simulations were performed offline, limiting the ability to draw inferences into how 1D vFFR may integrate into real-time assessment in the catheterization laboratory. Several cases failed to produce a 1D vFFR result with the porosity model, limiting statistical power whilst introducing potential bias. Ancillary analyses for quantifying determinants of agreement lacked statistical power and did not consider implicit model assumptions such as steady flow in rigid vessels. Invasive MVR was not available for vessels used in the primary outcome. As no pilot data existed in a matched cohort, a power calculation could not be performed for the porosity leakage function or for comparison of 1D and 3D models, these results are therefore hypothesis generating.

## 6. Conclusion

This study has reported agreement and diagnostic accuracy of novel 1D models of vFFR in a matched cohort. The addition of side branch flow derived from local anatomy conferred a modest benefit for agreement but did not significantly improve diagnostic accuracy. When used in a suitable patient cohort, 1D vFFR may act as an effective gatekeeper to invasive wire-based assessment but is unlikely to replace FFR without further development and accuracy improvement.

## Supplementary Material

cvaf168_Supplementary_Data

## Data Availability

The data underlying this article will be shared on reasonable request to the corresponding author.

## Appendix

### Models of flow

All models in this work have been developed from the seminal work of lambert^55^ examining 1D axial fluid flow models in non-rigid tubes. The 1D representation of the Navier–Stokes equations are given as:

(1)
$$\frac{\partial Q}{\partial x}+\;\frac{\partial A}{\partial t}=0$$

(2)
$$\frac{\partial Q}{\partial t}+\;\frac{\partial }{\partial x}\left(\frac{Q^{2}}{A}\right)=\;-\frac{A}{\rho }\frac{\partial P}{\partial x}-\;\frac{2(\zeta +2)\pi \mu }{\rho }\;\frac{Q}{A}$$

Where *Q*(*x*, *t*), *P*(*x*, *t*), and *A*(*x*, *t*) represent the flow, pressure and area of a vessel at position *x* and time *t*. For the models in this work we investigate the steady formulations due to their usage within the literature.^9,22^

### No leak model

The No-leak model, originally proposed by Hughes and Lubliner^56^ then extended in,^9,36^ now assumes a tapering vessel and the impact of a chosen velocity profile, with parameter ζ. The steady continuity and momentum equations can be formulated as:

(3)
$$\frac{dQ}{dx}=0$$

(4)
$$X\frac{d}{dx}\left(\frac{Q^{2}}{A}\right)+\frac{A}{\rho }\frac{dP}{dx}+\;\frac{2(\zeta +2)\pi \mu }{\rho }\;\frac{Q}{A}\;(1+R\prime^{2}{)}^{\frac{-1}{2}}=0$$

where X introduces non-linear effects from a chosen velocity profile, which is parametrized by ζ. In this work we use ζ = 4.31, which has previously shown to be optimum.^22^  *R*′ = dR/dx represents the rate at which a vessel tapers. The full numerical scheme utilized in this work can be found in.^36^

### Homogenous leak model

The inclusion of leak into the 1D models is necessitated by side branches from the coronary arteries, which can often not be resolved by imaging methods. This method assumes steady flow and uniform leak over the healthy parts of the vessel; its governing equations can be formulated as:

(5)
$$Q(x)=\;{\left(\frac{R(0)-\left(\;\frac{R(0)-R(L)}{L}\right)x}{R(0)}\right)}^{c}Q(0)$$

(6)
$$\frac{dQ}{dx}+\;\psi =0$$

(7)
$$X\frac{d}{dx}\left(\frac{Q^{2}}{A}\right)+\frac{A}{\rho }\frac{dP}{dx}+\;\frac{2(\zeta +2)\pi \mu }{\rho }\;\frac{Q}{A}\;(1+R\prime^{2}{)}^{\frac{-1}{2}}+\;\frac{Q}{A}\psi =0$$

where equation 5, represents our model of leak for the healthy vessel. The continuity equation in 6 now includes the additional source term *ψ* representing the leak from the vessel. Note this formulation is generalized from that first proposed by Gosling *et al*.^15^ in which the authors did not include vessel taper effects and took X = 1. The full numerical scheme utilized in this work can be found in.^36^

### Localized leak model

The localized anatomical approach, first proposed by Taylor *et al*.^17^ and generalized in^36^ now assumes that side branch is not uniformly distributed at different positions in the vessel; rather it varies with taper. The modified model of flow in this setting then becomes:

(8)
$$Q(x)=Q(0){\left(\frac{A(x)}{A(0)}\right)}^{\frac{c}{2}}$$

where *A*(*x*) represents the area of a vessel at curvilinear centreline position *x* and *c* is the Murray exponent, taken to *c* = 2.39.^23^ The full numerical scheme utilized in this work can be found in the work of Saxton *et al*.^36^

### Conductance leak model

In equation 6, the leak term is general to accommodate different formulations of leak. In the conductance model, derived in^17^, we assume leak takes the form:

(9)
$$\frac{dQ}{dx}=\;-K(x)P(x)$$

(10)
$$K(x)=\;\frac{c}{2}\;\frac{dlog(A)}{dx}\;\frac{Q(x)}{P(x)}$$

(11)
$$X\frac{d}{dx}\left(\frac{Q^{2}}{A}\right)+\frac{A}{\rho }\frac{dP}{dx}+\;\frac{2(\zeta +2)\pi \mu }{\rho }\;\frac{Q}{A}\;(1+R\prime^{2}{)}^{\frac{-1}{2}}=0$$

Above, instead of assuming there are distinct side branches we assume that the coronary arteries behave as porous layers. Thus, the function *K*(*x*) combines both anatomical and physiological information making it an effective hydraulic conductivity. To compute the conductivity of the vessel wall we first compute pressure and flow using the localized leak model, in a healthy representation of the vessel.

### Porosity leak model

The final model of leak investigated and novel theory investigated in this work assumes a Darcy dynamics to the wall porosity^57^:

(12)
$$\frac{dQ}{dx}=\;\frac{\mu \pi R(x)}{\alpha \beta \rho }-\;\frac{\mu \pi R(x)}{\alpha \beta \rho }{\left(1+\frac{4\beta \rho \alpha^{2}}{T\mu^{2}}P(x)\right)}^{\frac{1}{2}}$$

(13)
$$X\frac{d}{dx}\left(\frac{Q^{2}}{A}\right)+\frac{A}{\rho }\frac{dP}{dx}+\;\frac{2(\zeta +2)\pi \mu }{\rho }\;\frac{Q}{A}\;(1+R\prime^{2}{)}^{\frac{-1}{2}}=0$$

where *T*, *α*, and *β* represent the wall thickness, and the porosity and tortuosity of the material that comprises it. To obtain coefficient values, we performed a multi-stage calibration process to obtain a population-based vessel wall *α* and *β*. See the supplementary material for more information. Note that the porosity and conductance model are a set of coupled odes and thus have to be solved numerically using the Tsit5() algorithm^58^.

### Derivation

The porosity model may be represented as:

(14)
$$P(x)=\;\frac{T\mu }{\alpha }\left(\frac{\Delta Qs}{\Delta A}\right)+T\beta \rho {\left(\frac{\Delta Qs}{\Delta A}\right)}^{2}$$

Thus, we can represent the sequestered flow (Qs) as:

(15)
$$\Delta Qs=\;-\frac{dQ}{dx}dx,\;\Delta A=2\pi R(x)dx$$

(16)
$$\frac{\Delta Qs}{\Delta A}=\;-\frac{1}{2\pi R(x)}\;\frac{dQ}{dx}$$

We can then rewrite the pressure as:

(17)
$$P(x)=\;-\frac{T\mu }{\alpha 2\pi R}\;\frac{dQ}{dx}+\;\frac{T\beta \rho }{4\pi^{2}R^{2}}\;{\left(\frac{dQ}{dx}\right)}^{2}$$

This provides a quadratic equation, solving for $\frac{dQ}{dx}$:

(18)
$$\frac{dQ}{dx}=\;\frac{\mu \pi R(x)}{\alpha \beta \rho }-\;\frac{\mu \pi R(x)}{\alpha \beta \rho }{\left(1+\frac{4\beta \rho \alpha^{2}}{T\mu^{2}}P(x)\right)}^{\frac{1}{2}}$$

### Flow in stenosed vessel sections

Abrupt stenotic changes in *R*(*x*) involve significant radial flow, invalidating the 1D haemodynamics. A lumped sub-model of, essentially, a Bernoulli resistor can represent flow within the stenosis^25,59^:

(19)
$$\Delta P=AQ+BQ|Q|,\;\;\;\;\;\;A=\;\frac{K_{v}\mu }{A_{0}D_{0}},\;\;\;\;\;B=\;\frac{K_{t}\rho }{2A_{0}^{2}}\left(\frac{A_{0}}{A_{s}}-1\right)$$

Above, *A*_0_ and *A_s_* are the cross-sectional areas of the healthy and stenotic segments, respectively, *D*_0_ and *D_s_* are the healthy and stenotic vessel diameters and *K_v_* and *K_t_* are dimensionless empirical constants quantifying viscous and turbulent effects, respectively:

$$K_{v}=\;\frac{\left(K_{v1}S_{c}+\;K_{v2}D_{s}\right)}{D_{0}}{\left(\frac{A_{0}}{A_{s}}\right)}^{2},\;\;K_{v1}=26.56,\;\;\;K_{v2}=52.48,\;\;\;K_{t}=1.52\;$$

and *S_c_* is the length of the stenosis. No leak is assumed to occur in stenosed sections of vessels.
