# Supplementary material for: Invasive validation of novel 1D models for computation of coronary fractional flow reserve
Source: Cardiovasc Res. 2025 Sep 23;121(14):2233–45. doi: 10.1093/cvr/cvaf168 (PMC12638729; doi:10.1093/cvr/cvaf168)
Supplement: cvaf168_Supplementary_Data [file cvaf168_supplementary_data.pdf]

1D Validation Supplementary Material

Supplementary 1: Parameterisation of porosity flow

The below schematic demonstrates the additional parameterisation of the wall porosity (b) and tortuosity (k) required for the porosity flow model. The process begins with a nominal inlet flow (Qin) which computes vFFR and along with outlet flow, microvascular resistance (Rμ). If the computed and NARMAX-derived Rμ are discordant, the inlet flow is accordingly adjusted within the physiological bounds (50 - 450 mL/min) and the cycle is repeated until computed and NARMAX-derived Rμ results are concordant. The process is then repeated, by optimising vFFR against measured FFR. However, as the now inlet flow value is now fixed, b and k parameters are adjusted to improve vFFR and FFR concordance. This was repeated on a personalised basis for every included case and mean b and k values were then used to parameterise the porosity leakage function for invasive validation. Consequently, for invasive validation results, the only boundary condition directly acquired from invasive data was Pa.

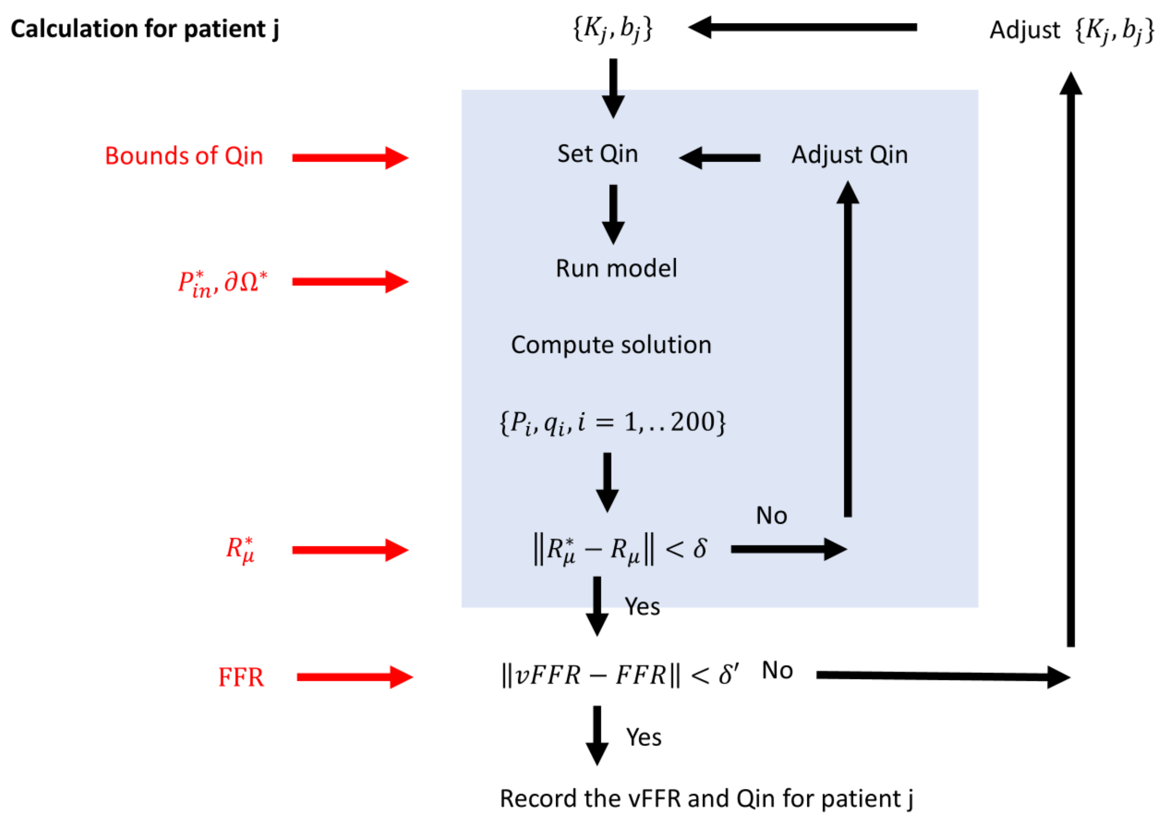

Schematic demonstrating inlet flow optimisation against computed and NARMAX-derived microvascular resistance (blue shaded box) and a second cycle of b and k parameterisation, optimised against invasive FFR for the porous leak model.

Supplementary 2 healthy vessel filtration code in MATLAB

MATLAB, Natick, Massachusetts: The MathWorks Inc.

```
% raw s i g n a l from t h e v t k f i l e from V i r t u
dx = d i f f ( book1 ( : , 1 ) ) ;
dy = d i f f ( book1 ( : , 2 ) ) ;
dz = d i f f ( book1 ( : , 3 ) ) ;
r = book1 ( : , 4 ) ;
d s = ( dx . * dx + dy . * dy + dz . * dz ) . ^ 0 . 5 ;
s = z e r o s ( 1 , l e n g t h ( r ) ) ;
f o r i = 2 : l e n g t h ( r )
s ( i ) = s ( i −1) + d s ( i − 1 ) ;
end
s 0 = max ( s ) ;
N p o i n t s = 2 0 0 ;
s _1 = [ 0 : 1 : N p o i n t s ] / N p o i n t s * s 0 ;
r _ 1 = i n t e r p 1 ( s , r , s _ 1 ) ;
r_max = r _ 1 ( 1 ) ;
r_min = r _ 1 ( end ) ;
% A t t a c h i n l e t and o u t l e t m a n i f o l d s , t o c o n f i n e Gibbs ’ phenomenon
i n _ l e n g t h = 4 0 ;
e n t r y = z e r o s ( 1 , i n _ l e n g t h ) ; %o n e s ( 1 , i n _ l e n g t h ) * r_max ;
e n t r y _ p o s = [ − i n _ l e n g t h : 1 : − 1 ] / N p o i n t s * s 0 ;
e x i t = z e r o s ( 1 , i n _ l e n g t h ) ; %o n e s ( 1 , i n _ l e n g t h ) * r_min ;
e x i t _ p o s = [ N p o i n t s + 1 : 1 : N p o i n t s + i n _ l e n g t h ] / N p o i n t s * s 0 ;
wk_pos = [ e n t r y _ p o s , s , e x i t _ p o s ] ;
% Workspace f o r e x t e n d e d d i a t a n c e v a r i a b l e
wk_rad = [ e n t r y , r_1 , e x i t ] ;
% Workspace f o r e x t e n d e d r a d i a u v a r i a b l e
% Take FFT
Fs = ( N p o i n t s +1+2* i n _ l e n g t h ) ;
T = 1 / Fs ;
% s p a t i a l s a m p l i n g " p e r i o d " m
L = N p o i n t s +1+2* i n _ l e n g t h ;
t = ( 0 : L−1)*T ;
% L e n g t h v e c t o r
s i g n a l = wk_rad ;
Y = f f t ( s i g n a l ) ;
P2 = a b s (Y/ L ) ;
% Compute t h e two − s i d e d s p e c t r u m P2 . . .
P1 = P2 ( 1 : L / 2 + 1 ) ; P1 ( 2 : end −1) = 2* P1 ( 2 : end − 1 ) ;
% . . . and t h e s i n g l e − s i d e d s p e c t r u m P1 , b a s e d on P2 and t h e even − v a l u e d s i g n a l l e n
g t h L .
% . . c h e c k by p l o t t i n g FFT and r e c o n s t r u c t i o n
f = Fs * ( 0 : ( L / 2 ) ) / L ;
% f i l t r a t i o n a p p l i e d i n F o u r i e r s p a c e
f _ c u t _ o f f = 1 3 8 ;
% two − s i d e d t o p − h a t f i l t e r w i t h a s s i g n e d c u t − o f f .
f i l t e r = ( f <= f _ c u t _ o f f ) ; wk1 = f l i p ( f i l t e r ) ; wk1 ( 1 ) = [ ] ; f i l t e r = d o u b l e ( [ wk1 , f i l t e r ] ) ;
Yp = Y . * (1 − f i l t e r ) ;
% . . . and r e c o n s t r u c t
r e c o n 1 = i f f t ( Yp ) ;
% f i l t e r e d
r e c o n 2 = i f f t (Y ) ;
% raw
% . . . s t r i p − o u t t h e m a n i f o l d s , t r u n c a t e any i m a g i n a r y f a l l − o u t , r e s t o r e
% e x p o n e n t i a l d e c a y
r e c o n 1 ( 1 : i n _ l e n g t h −1) = [ ] ;
r e c o n 1 ( N p o i n t s + 2 : end ) = [ ] ;
r e c o n 1 = r e a l ( r e c o n 1 ) ;
r e c o n _ r e a l = r e c o n 1 ;
r _ 1 = r _ 1 + r _ m o d e l ;
r e c o n _ r e a l = r e c o n _ r e a l + r _ m o d e l ;
```

Supplementary 3 Included cases

Each case contains both angiographic images used for vessel reconstruction, with the reconstruction centreline back projected as a green line. The adjacent graph shows radius as a function of centreline location in metres. The blue line represents reconstruction radius, and the green line Fourier filtered modelled healthy radius.

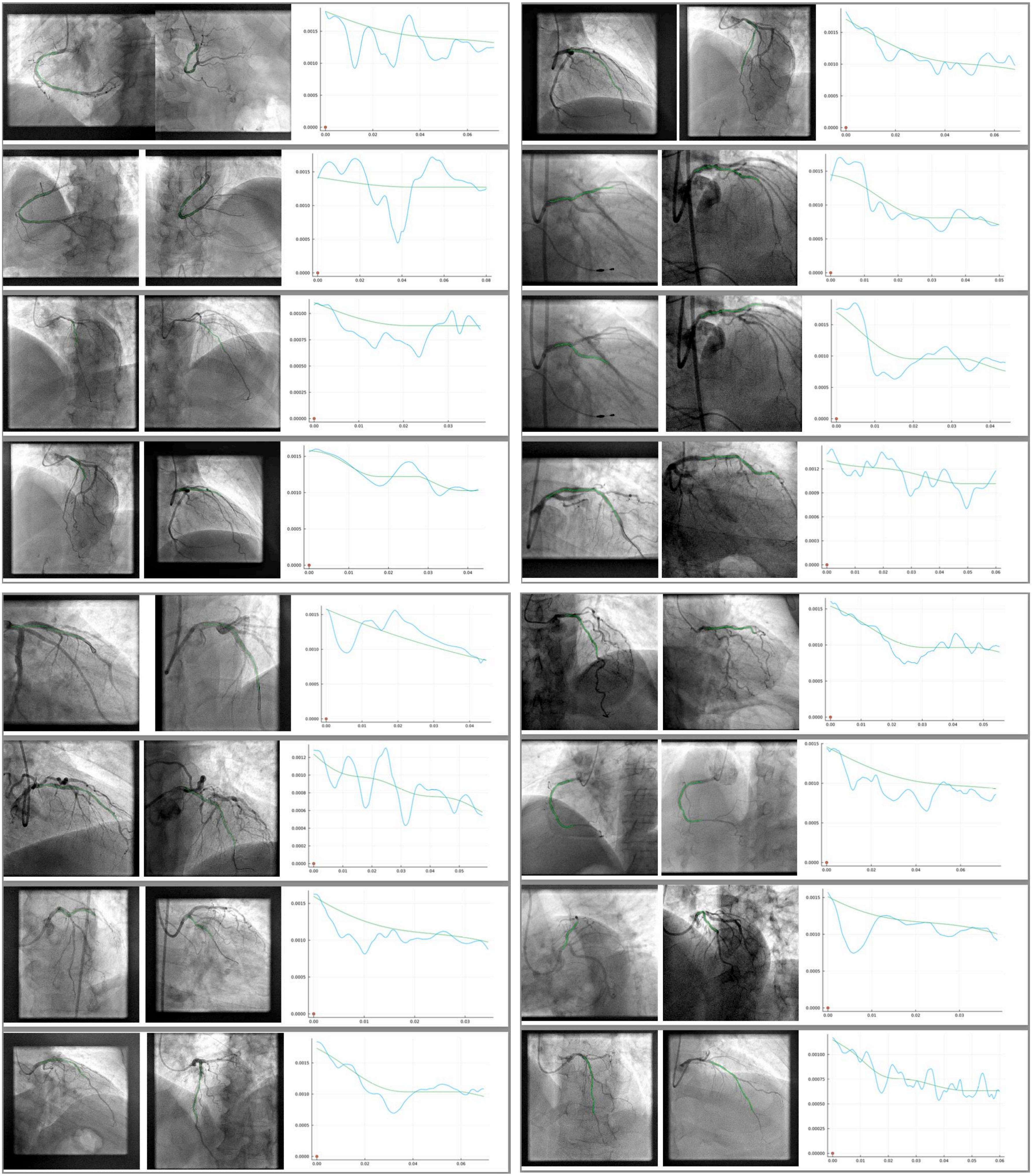

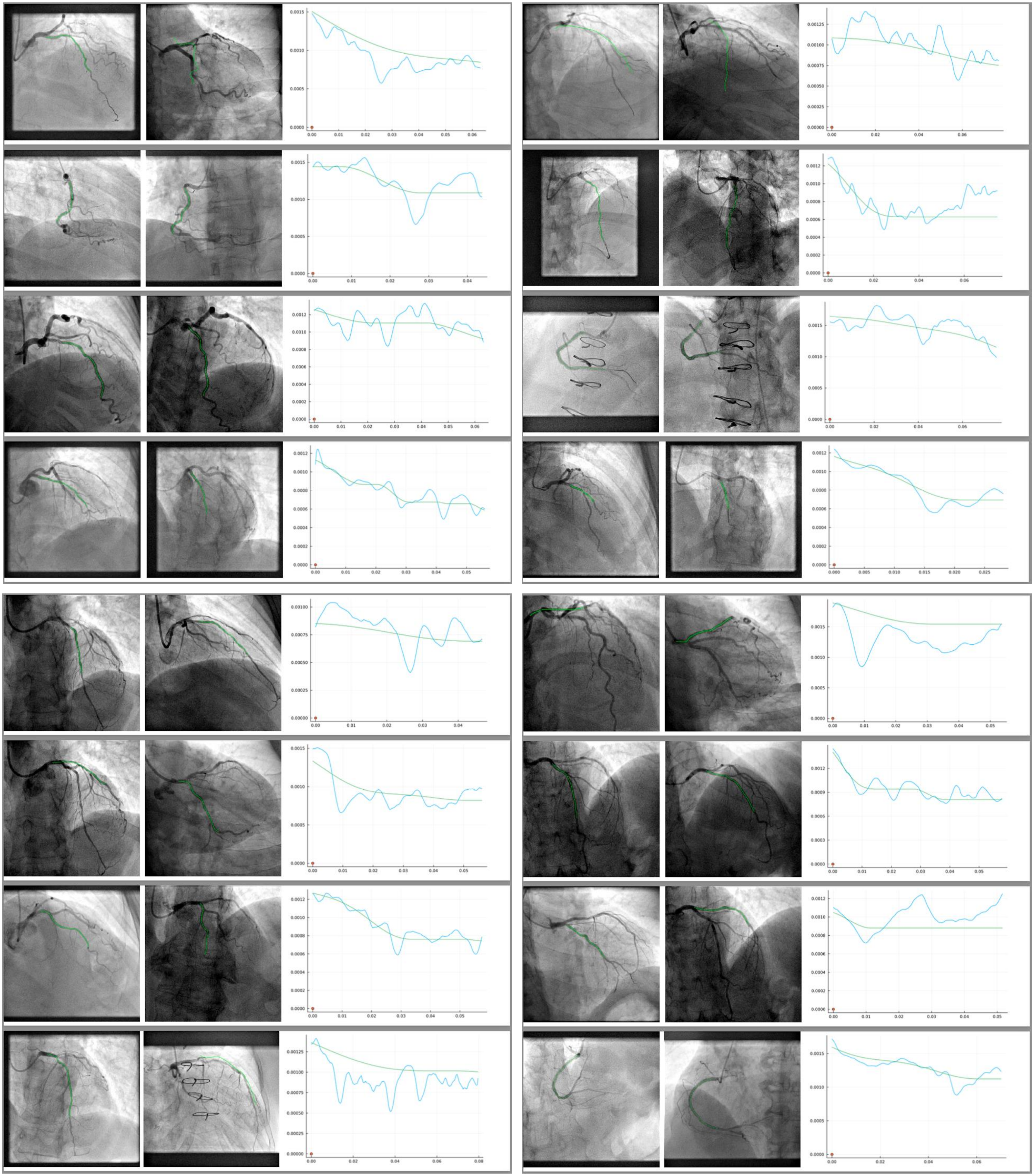

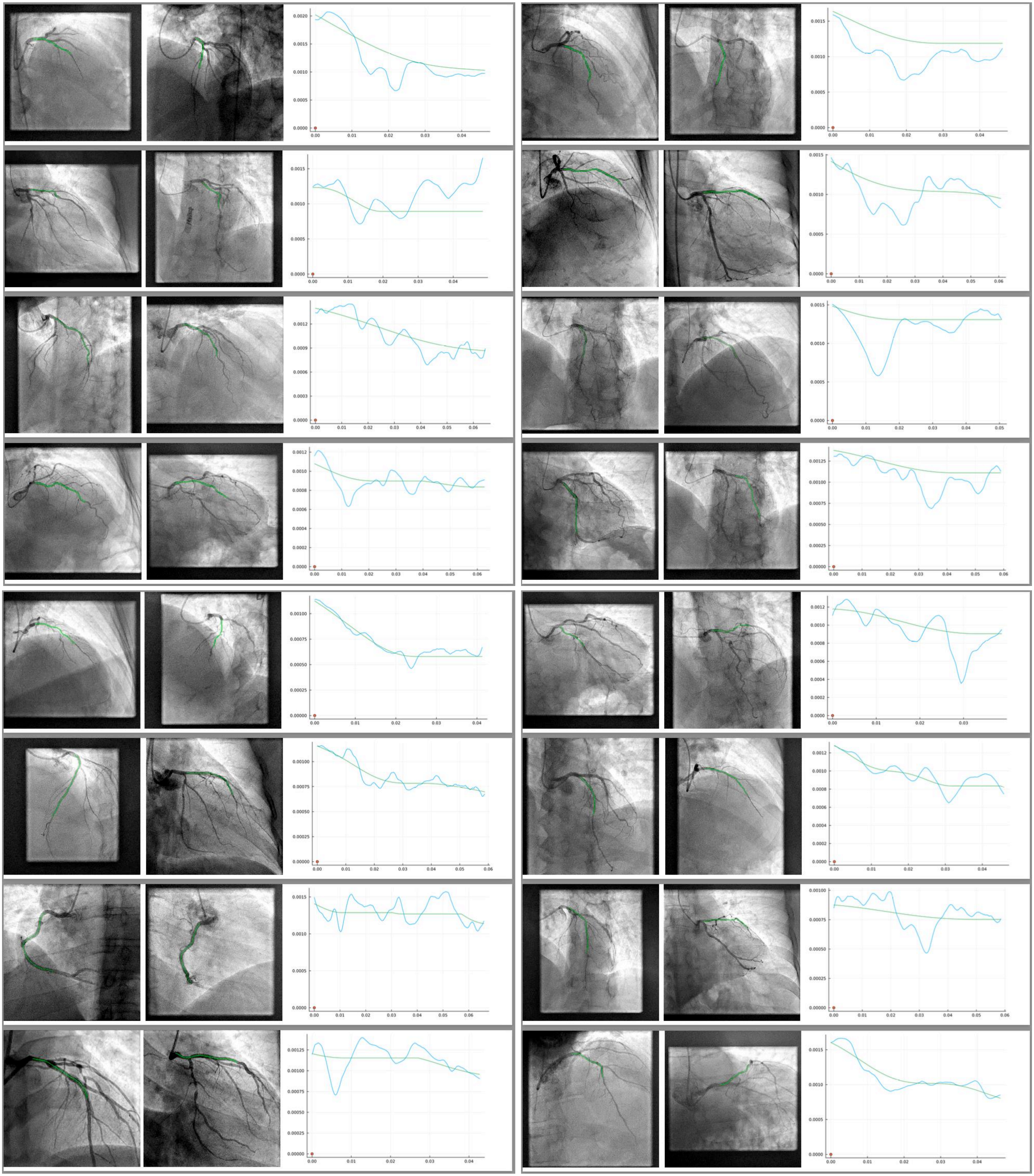

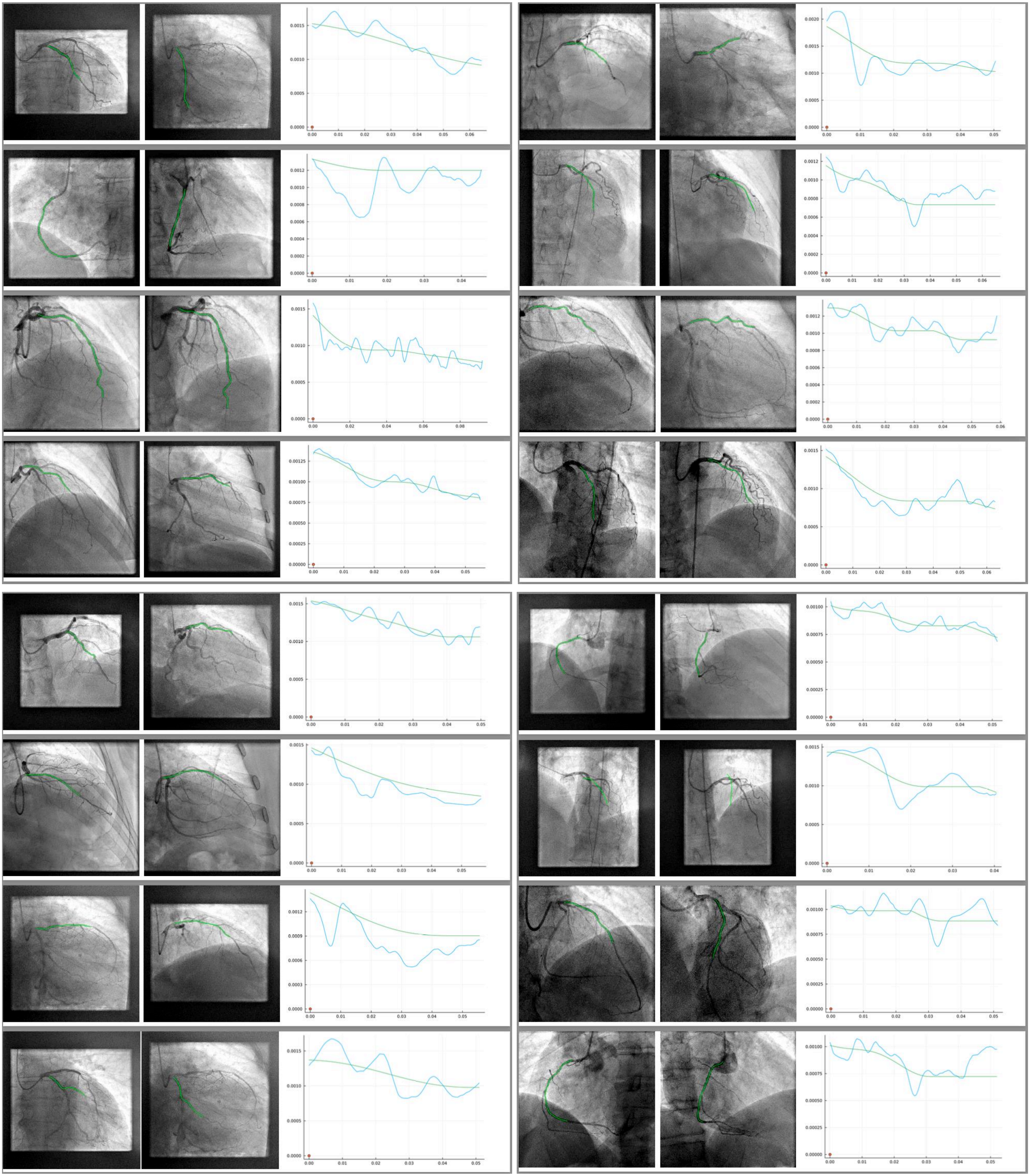

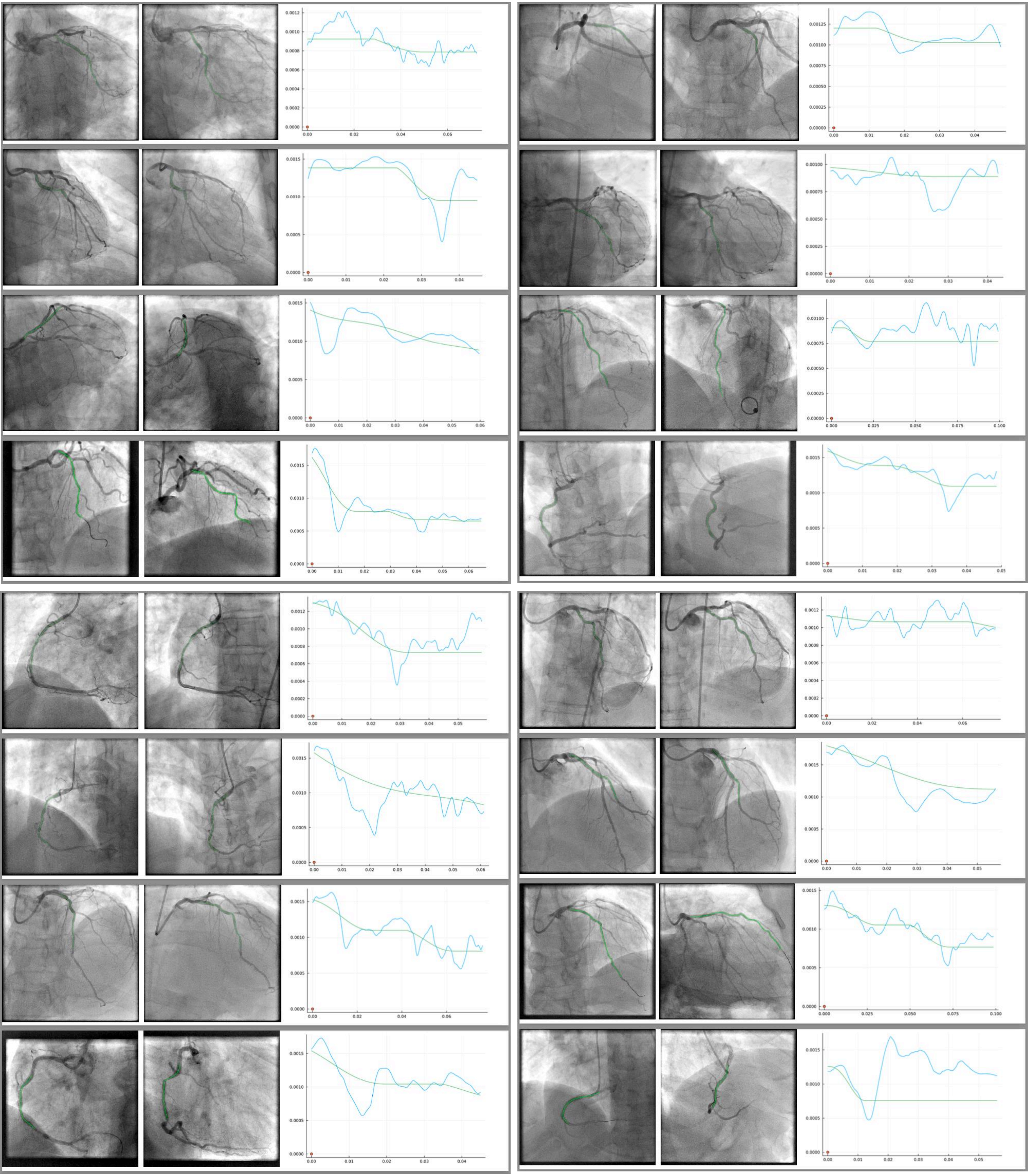

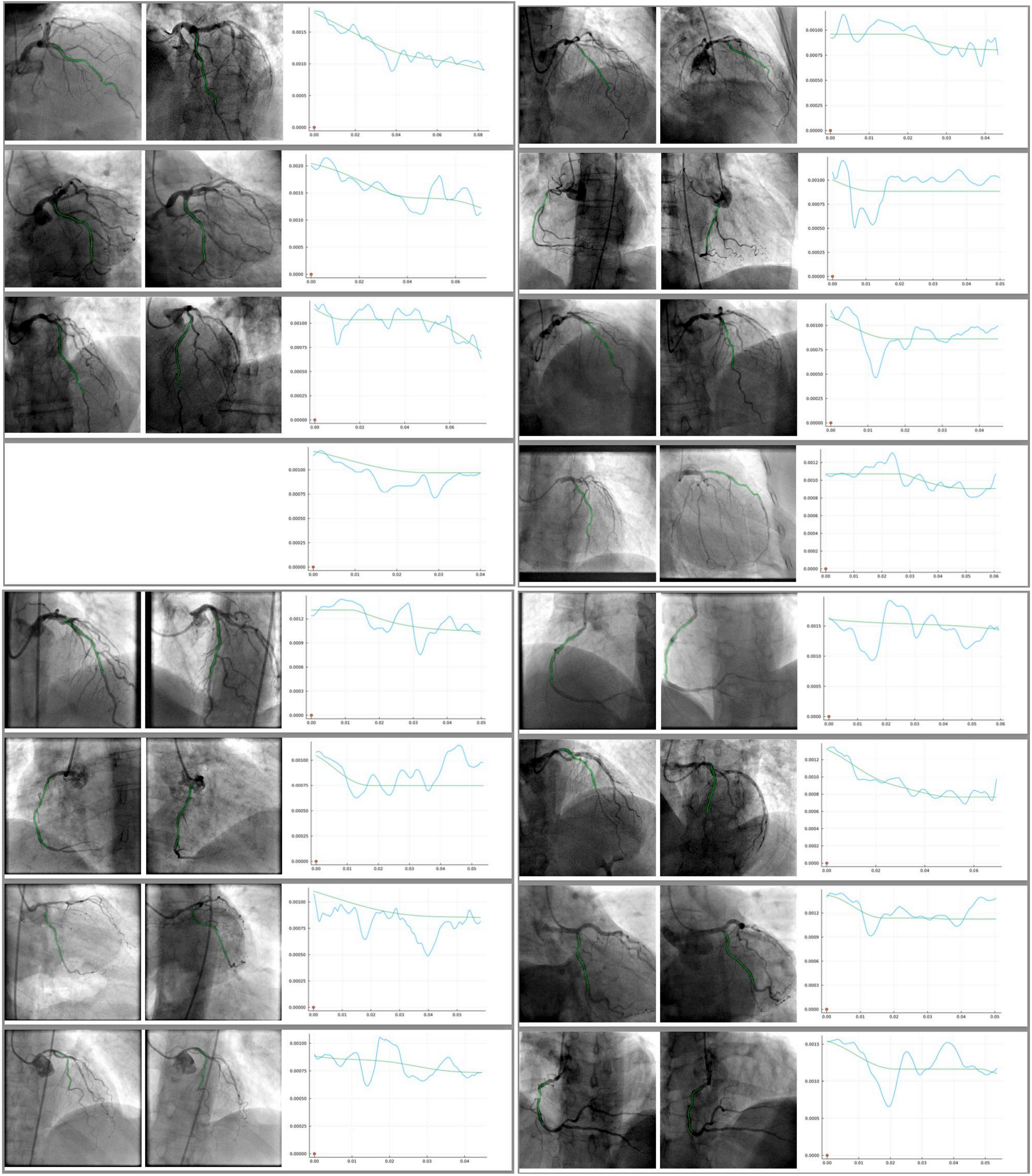

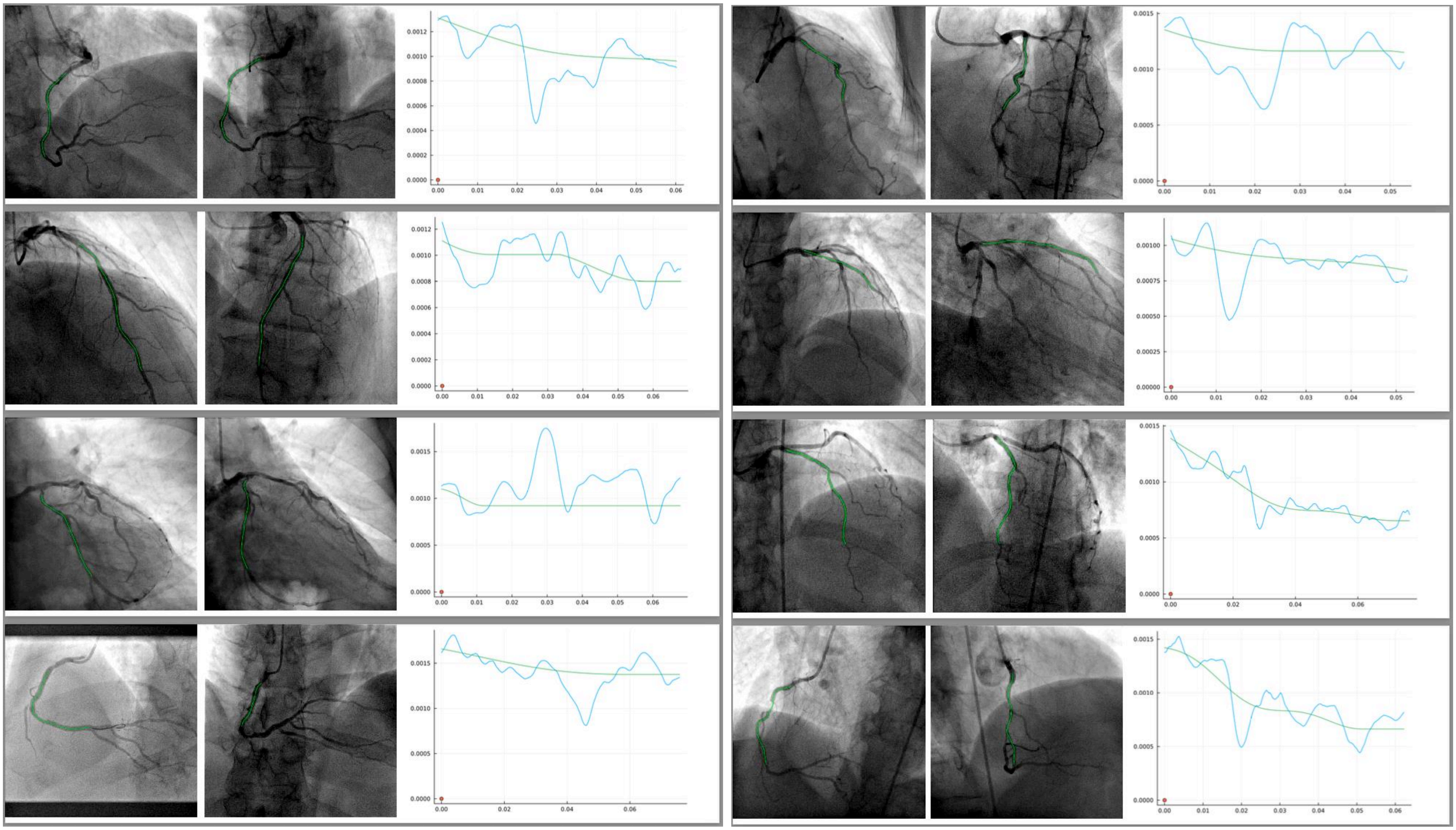

Supplementary 4 distribution of FFR and Pd/Pa values

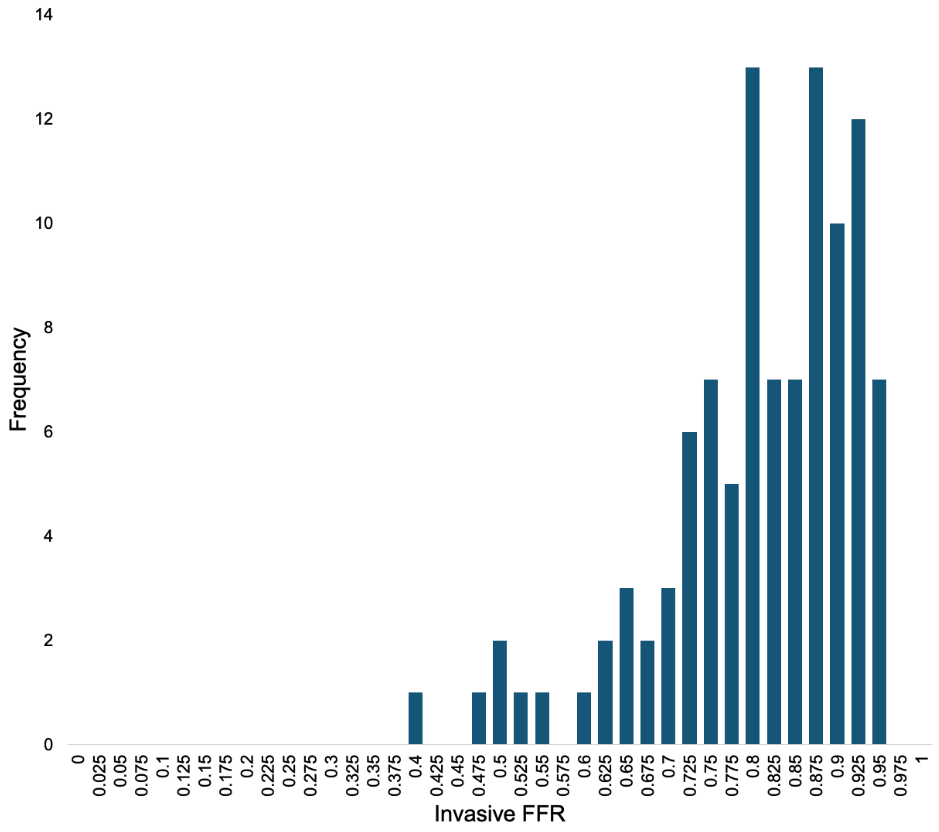

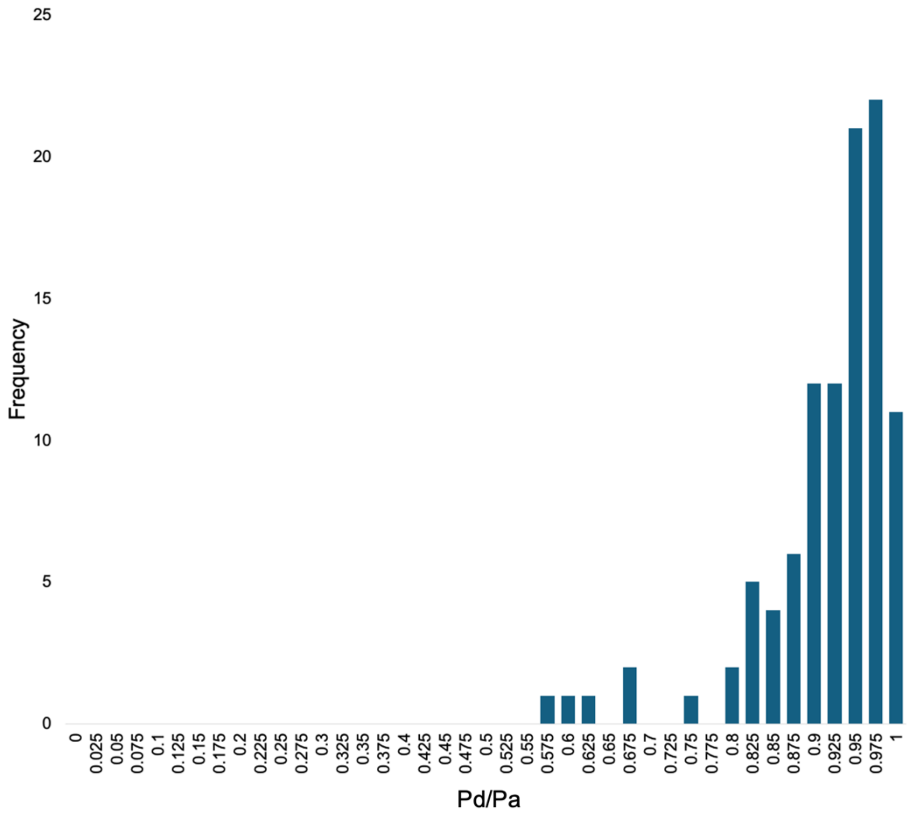

Supplementary 5 distribution of 1D vFFR values

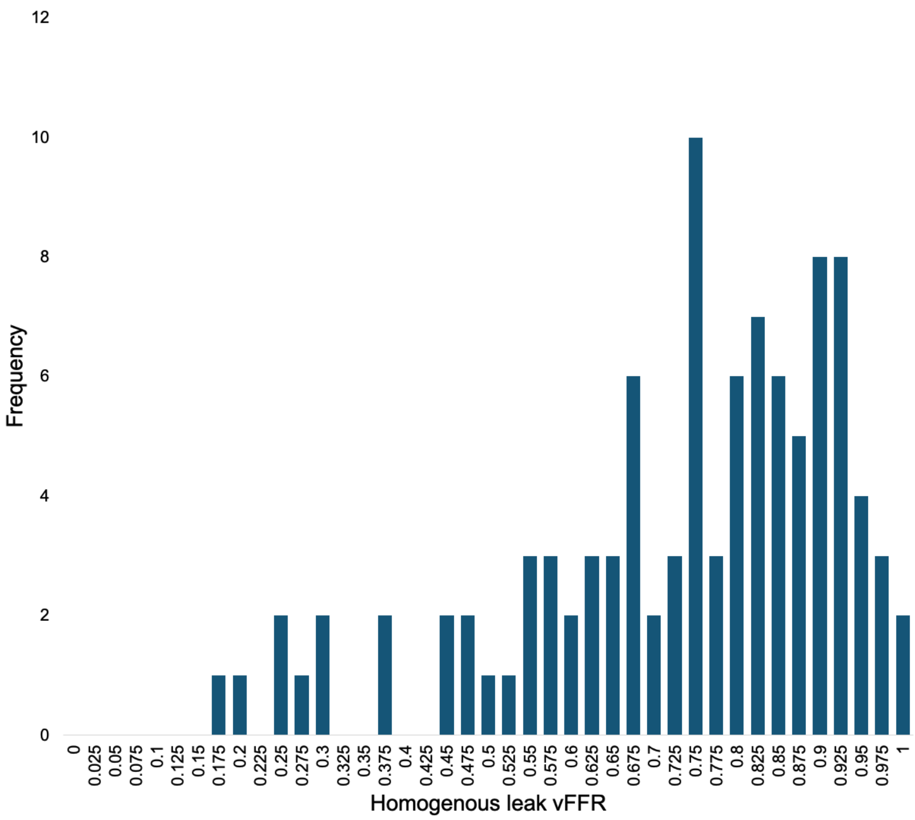

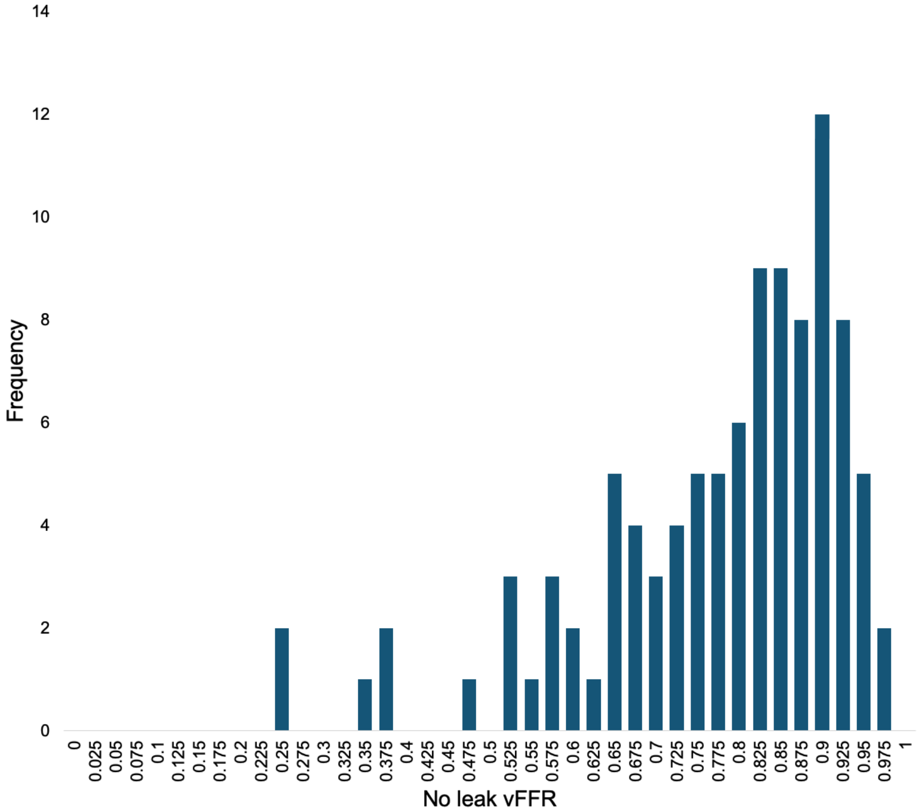

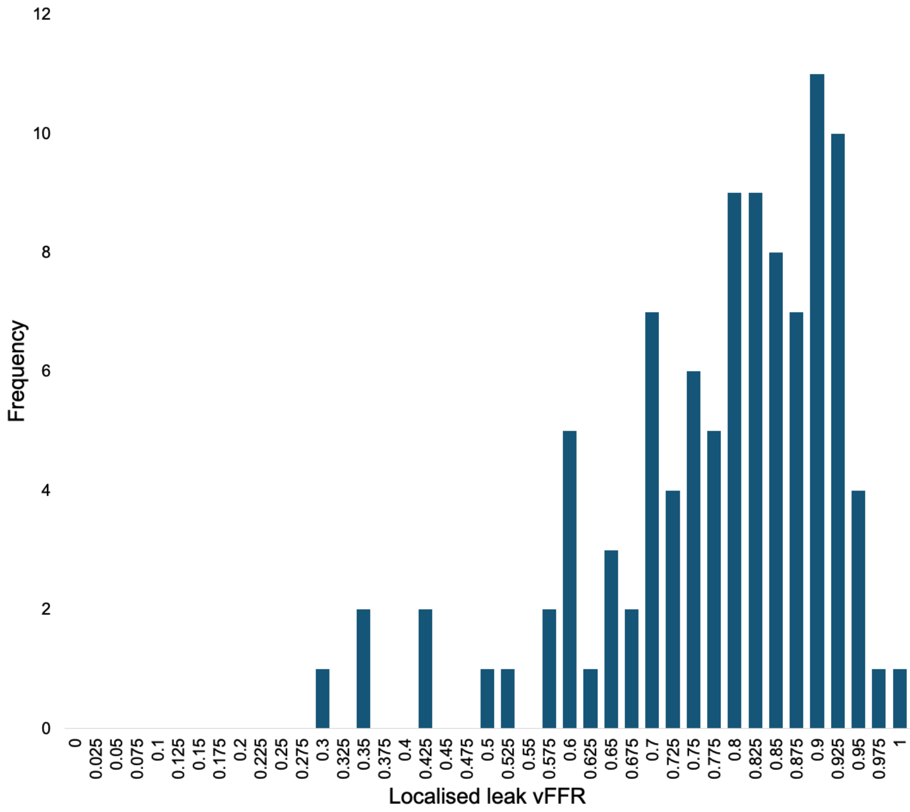
Frequency
12
10
8
6
4
2
0
0 0.025 0.05 0.075 0.1 0.125 0.15 0.175 0.2 0.225 0.25 0.275 0.3 0.325 0.35 0.375 0.4 0.425 0.45 0.475 0.5 0.525 0.55 0.575 0.6 0.625 0.65 0.675 0.7 0.725 0.75 0.775 0.8 0.825 0.85 0.875 0.9 0.925 0.95 0.975 1
Localised leak vFFR

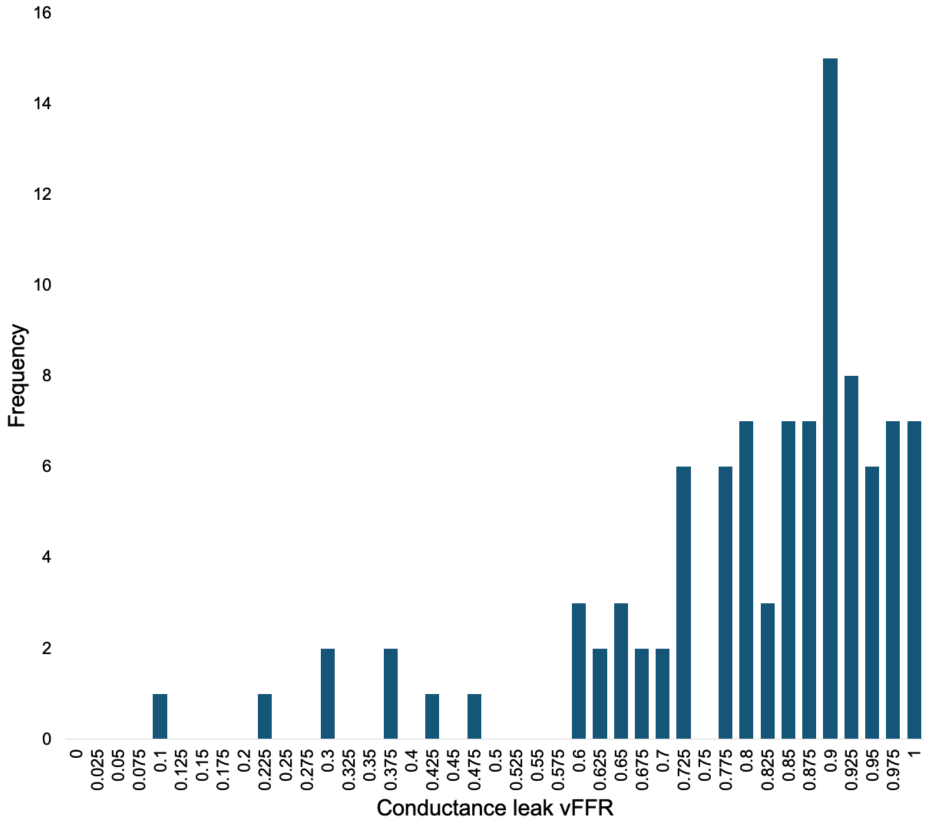
Frequency
16
14
12
10
8
6
4
2
0
0 0.025 0.05 0.075 0.1 0.125 0.15 0.175 0.2 0.225 0.25 0.275 0.3 0.325 0.35 0.375 0.4 0.425 0.45 0.475 0.5 0.525 0.55 0.575 0.6 0.625 0.65 0.675 0.7 0.725 0.75 0.775 0.8 0.825 0.85 0.875 0.9 0.925 0.95 0.975 1
Conductance leak vFFR

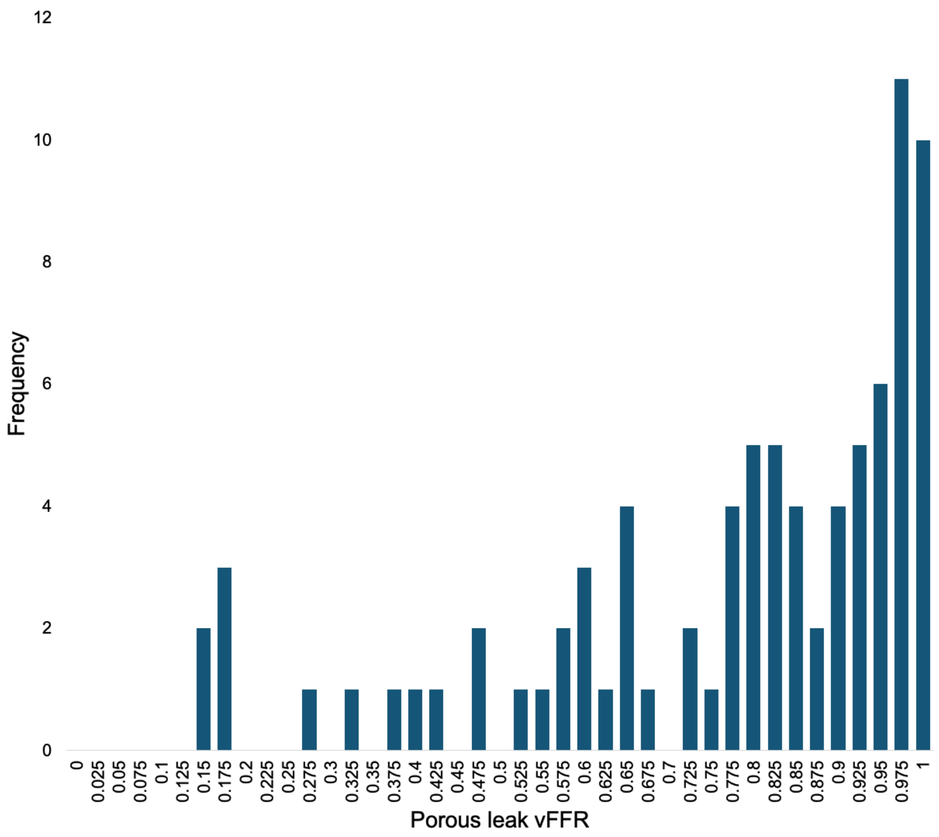

Frequency
Porous leak vFFR
0
2
4
6
8
10
12
0
0.025
0.05
0.075
0.1
0.125
0.15
0.175
0.2
0.225
0.25
0.275
0.3
0.325
0.35
0.375
0.4
0.425
0.45
0.475
0.5
0.525
0.55
0.575
0.6
0.625
0.65
0.675
0.7
0.725
0.75
0.775
0.8
0.825
0.85
0.875
0.9
0.925
0.95
0.975
1

Supplementary 6 Quantile regression Bland Altman results

	Median quantile	2.5th quantile	97.5th quantile
No leak	y = 0.50x – 0.45	y = 1.47x – 1.42	y = -0.75x + 0.79
Homogenous leak	y = 0.70x – 0.62	y = 1.62x – 1.54	y = -0.63x + 0.72
Localised leak	y = 0.31x – 0.27	y = 1.41x – 0.36	y = -1.14x + 1.11
Conductance leak	y = 0.50x – 0.41	y = 1.36x – 1.31	y = -1.12x + 1.20
Porosity leak	y = 0.80x – 0.66	y = 2.00x – 1.84	y = -0.21 + 0.47

All values represent equations of a straight line for quantile regression limits and median bias.

Supplementary 7 Bland Altman and Passing and Bablok regression results for all models of flow

Data show Bland Altman limits of agreement calculated using the original Bland-Altman technique and are presented for reference

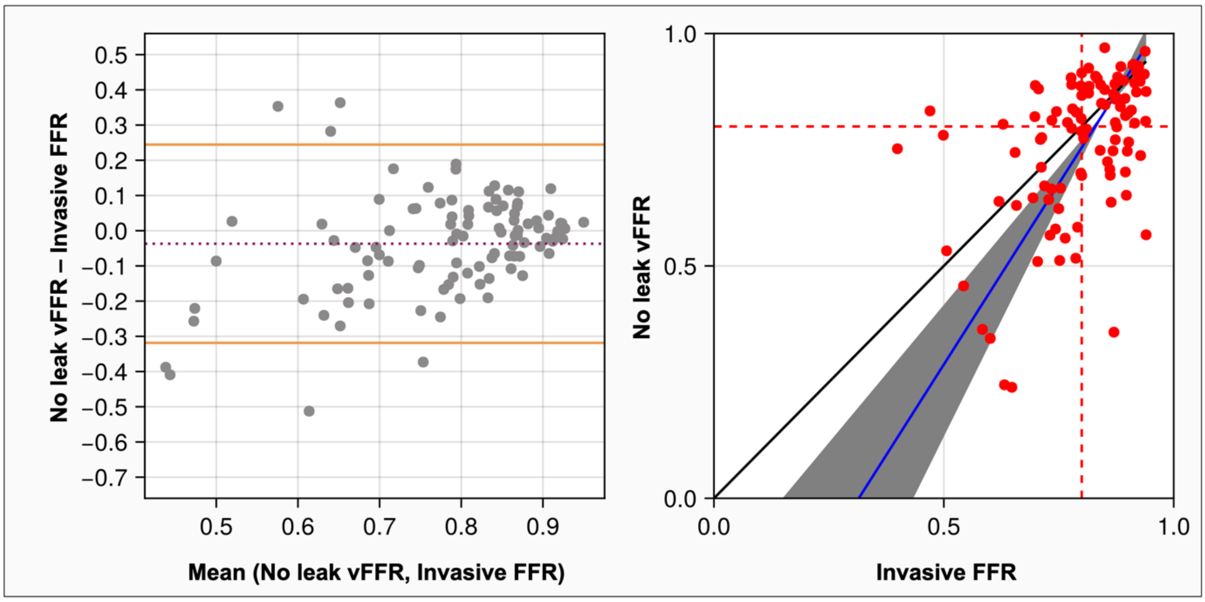

S7 figure 1. Bland Altman and Passing and Bablok regression comparing invasive FFR with no leak vFFR. No leak vFFR underestimated FFR by -0.04 (95% LOA -0.31 to 0.24). Passing and Bablok regression identified significant proportional and constant differences between techniques (m coefficient = 1.56 (95% CI 1.19 to 2.00), c coefficient = -0.49 (95% CI -0.87 to -0.18)).

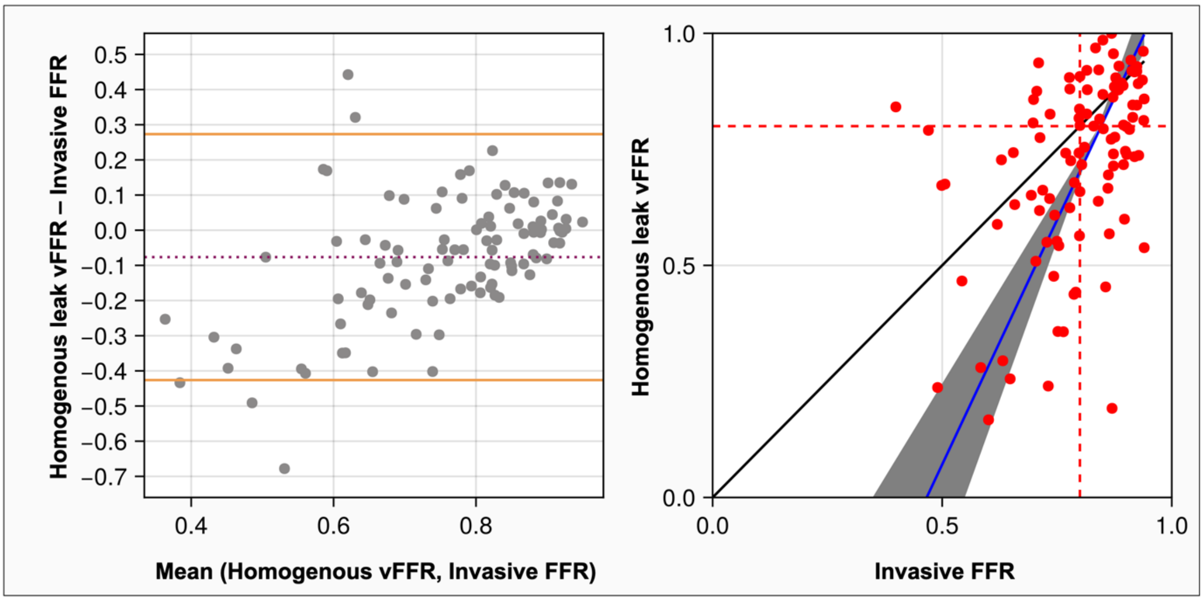

S7 figure 2. Bland Altman and Passing and Bablok regression comparing invasive FFR with homogenous leak vFFR. Homogenous leak vFFR underestimated FFR by -0.08 (95% LOA -0.43 to 0.27). Passing and Bablok regression identified significant proportional and constant differences between techniques (m coefficient = 2.11 (95% CI 1.62 to 2.75), c coefficient = -0.98 (95% CI -1.51 to -0.56)).

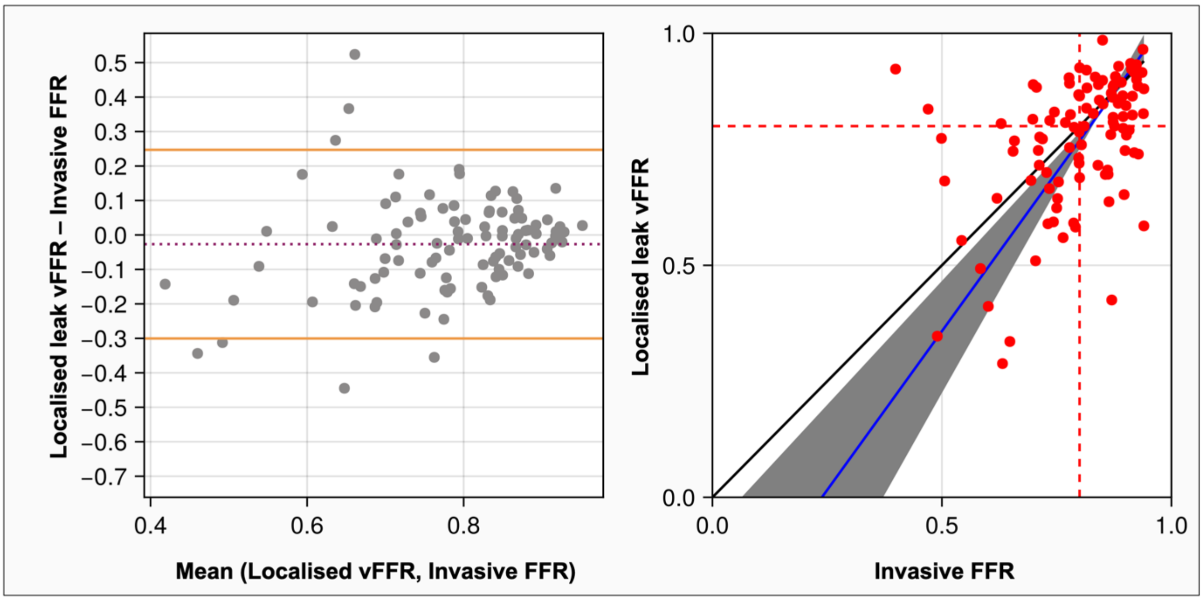

S7 figure 3. Bland Altman and Passing and Bablok regression comparing invasive FFR with localised leak vFFR. Localised leak vFFR underestimated FFR by -0.03 (95% LOA -0.30 to 0.25). Passing and Bablok regression identified significant proportional and constant differences between techniques (m coefficient = 1.37 (95% CI 1.07 to 1.75), c coefficient = -0.33 (95% CI -0.65 to -0.07)).

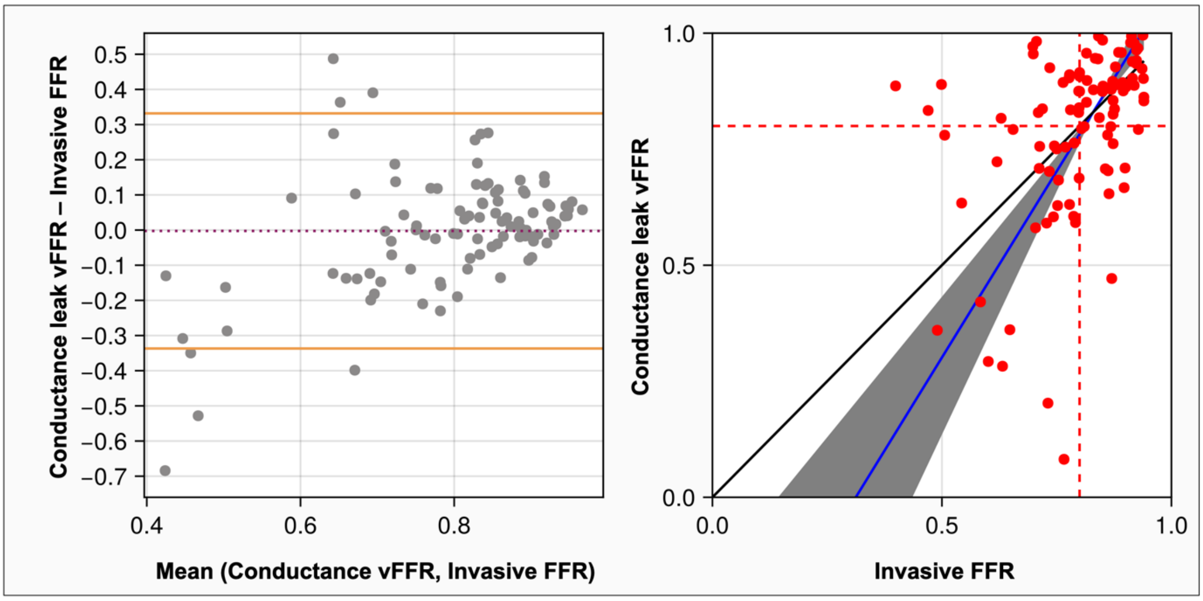

S7 figure 4. Bland Altman and Passing and Bablok regression comparing invasive FFR with conductance leak vFFR. Conductance leak vFFR showed zero overall bias versus FFR (95% LOA -0.34 to 0.33). Passing and Bablok regression identified significant proportional and constant differences between techniques (m coefficient = 1.60 (95% CI 1.21 to 2.11), c coefficient = -0.50 (95% CI -0.91 to -0.17)).

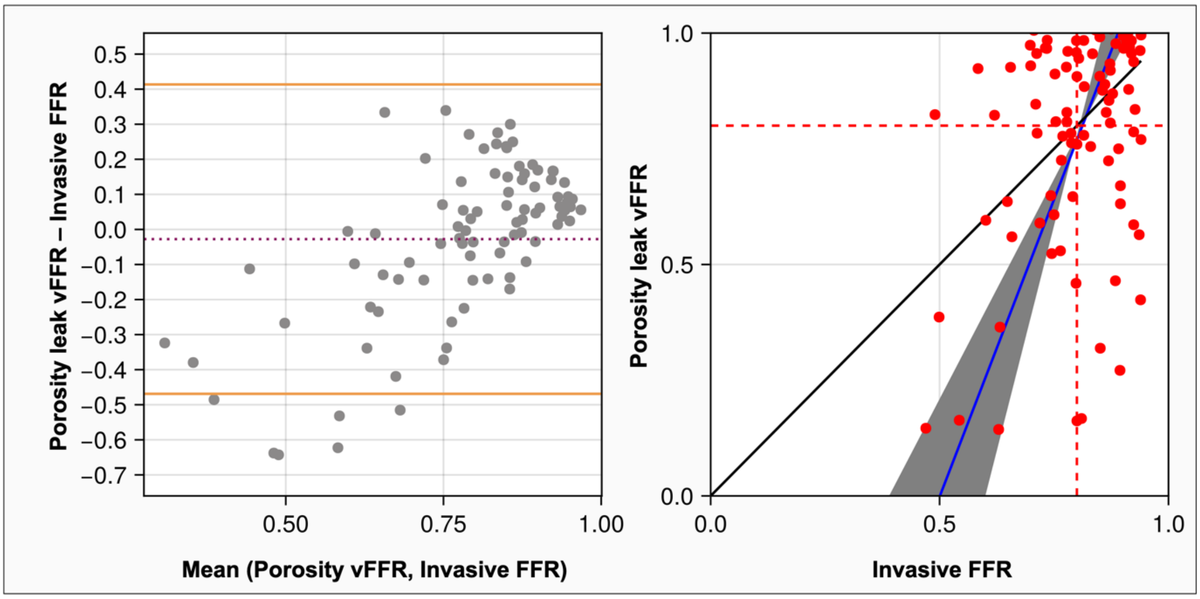

S7 figure 5. Bland Altman and Passing and Bablok regression comparing invasive FFR with porous leak vFFR. Porous leak vFFR underestimated FFR by -0.03 (95% LOA -0.47 to 0.41). Passing and Bablok regression identified significant proportional and constant differences between techniques (m coefficient = 2.57 (95% CI 1.90 to 3.89), c coefficient = -1.29 (95% CI -2.33 to -0.74)).

Supplementary 8 1D model diagnostic accuracy variation with FFR

All figures display diagnostic accuracy against FFR which is presented as a moving average of 20 cases

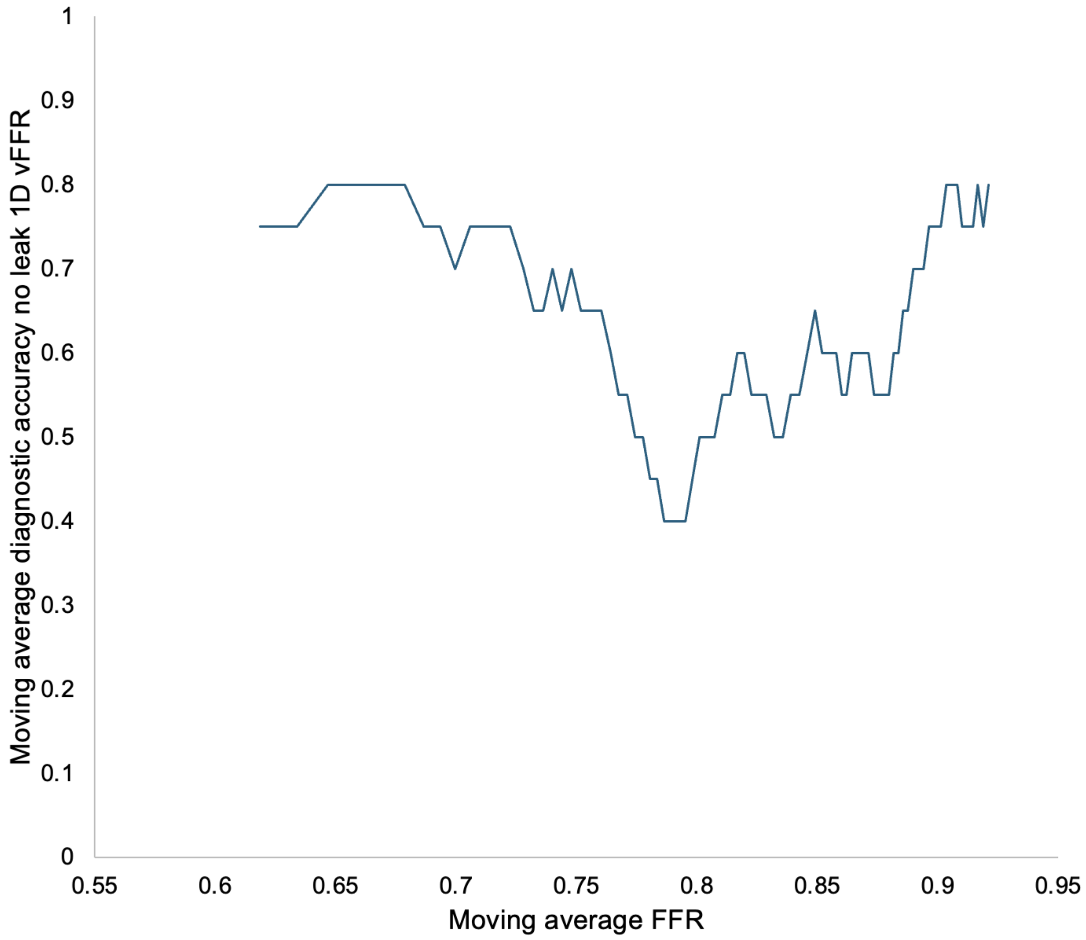

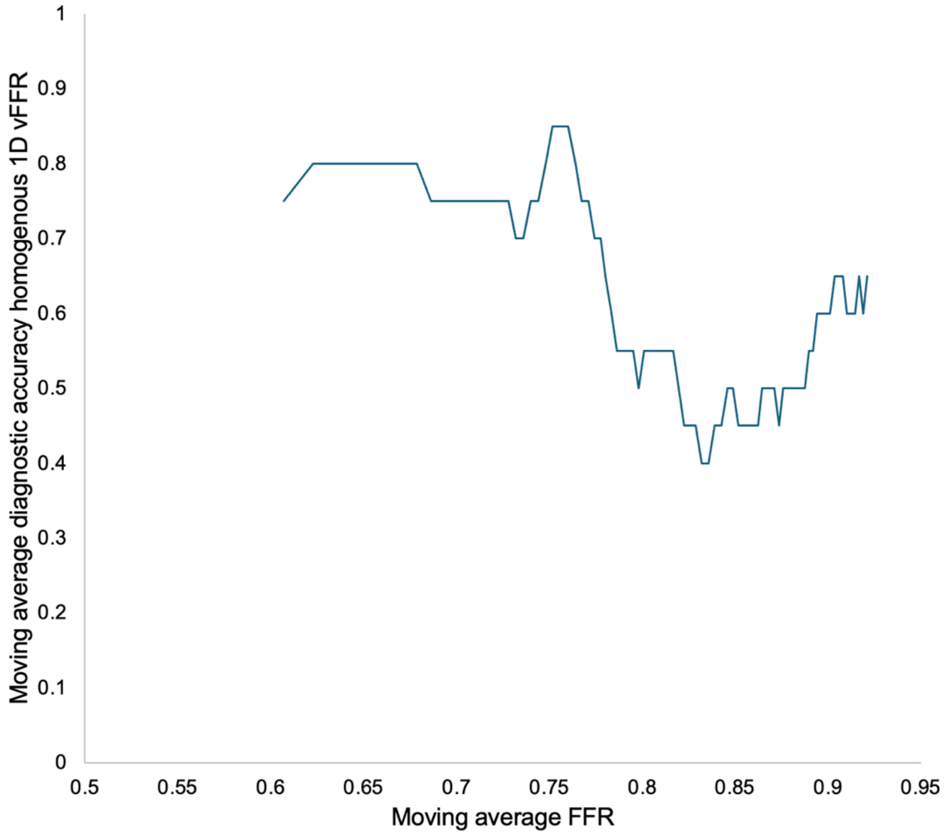
Moving average diagnostic accuracy homogenous 1D vFFR
Moving average FFR
0
0.1
0.2
0.3
0.4
0.5
0.6
0.7
0.8
0.9
1
0.5
0.55
0.6
0.65
0.7
0.75
0.8
0.85
0.9
0.95

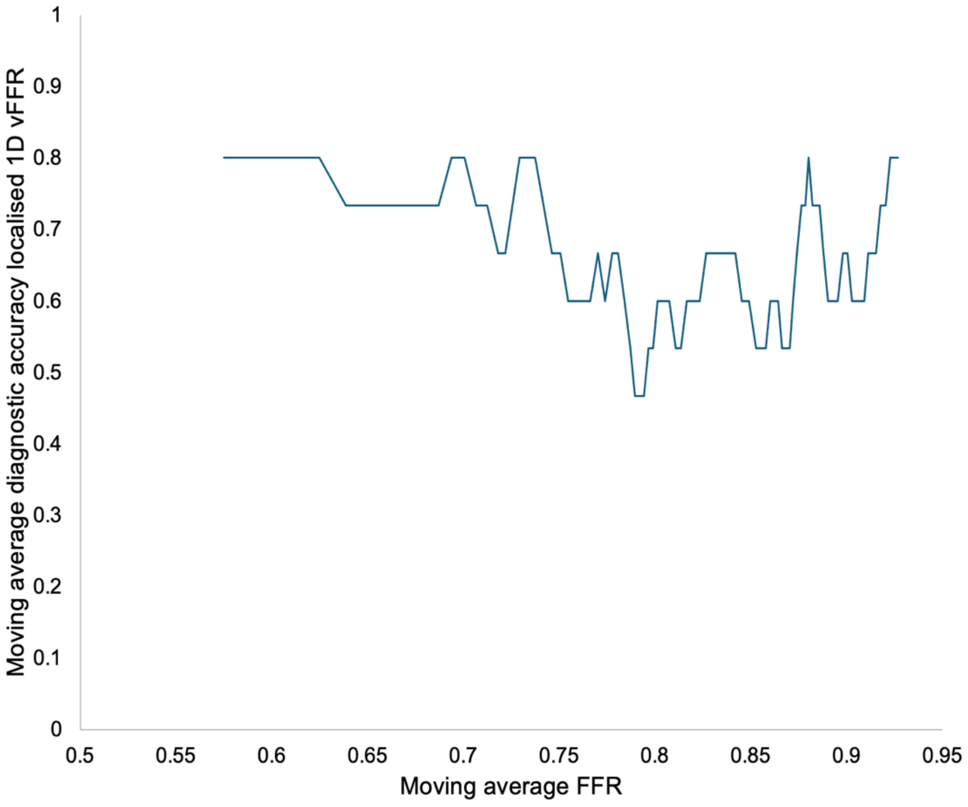
Moving average diagnostic accuracy localised 1D vFFR
Moving average FFR
0
0.1
0.2
0.3
0.4
0.5
0.6
0.7
0.8
0.9
1
0.5
0.55
0.6
0.65
0.7
0.75
0.8
0.85
0.9
0.95

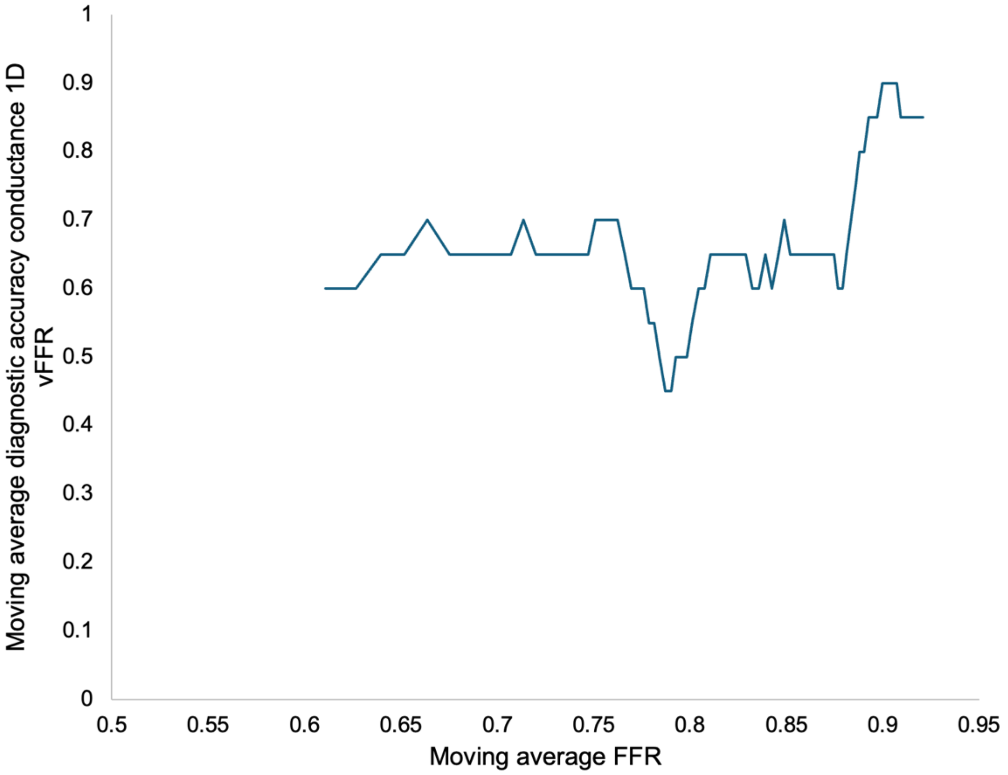

Moving average diagnostic accuracy conductance 1D vFFR
Moving average FFR
1
0.9
0.8
0.7
0.6
0.5
0.4
0.3
0.2
0.1
0
0.5
0.55
0.6
0.65
0.7
0.75
0.8
0.85
0.9
0.95

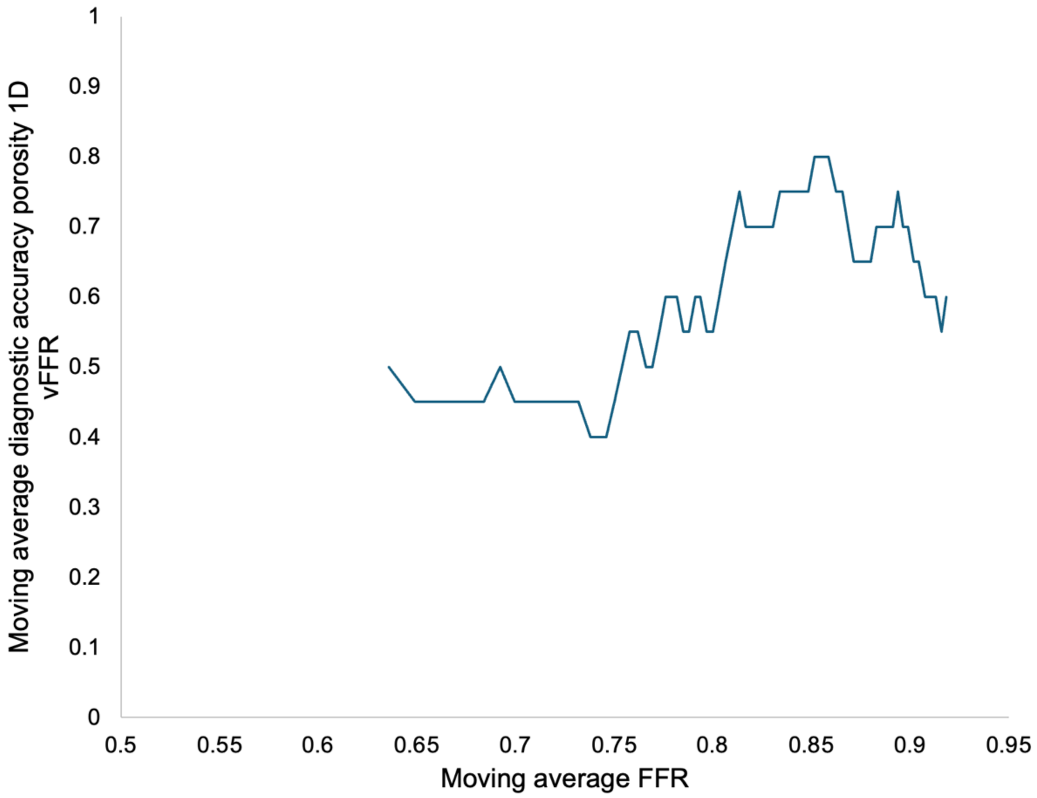

Moving average diagnostic accuracy porosity 1D vFFR
Moving average FFR
1
0.9
0.8
0.7
0.6
0.5
0.4
0.3
0.2
0.1
0
0.5
0.55
0.6
0.65
0.7
0.75
0.8
0.85
0.9
0.95

Supplementary 9 logistic regression results

Models	No-Leak		Homogenous		Anatomical		Conductance		Porosity	
Parameter	Log-odds (95% CI)	P-value	Log-odds (95% CI)	P-value	Log-odds (95% CI)	P-value	Log-odds (95% CI)	P-value	Log-odds (95% CI)	P-value
Male	0.60 (-0.61, 1.81)	0.334	-0.09 (-1.27, 1.09)	0.878	-0.03 (-1.21, 1.14)	0.957	0.24 (-1.01, 1.48)	0.709	0.03 (-1.21, 1.26)	0.968
Female	-1.27 (-2.47, -0.06)	0.039	-1.39 (-2.57, -0.21)	0.210	-0.71 (-1.88, 0.47)	0.239	-1.19 (-2.43, 0.05)	0.061	-0.38 (-1.62, 0.85)	0.543
Age	-0.02 (-0.08, 0.05)	0.591	0.01 (-0.05, 0.07)	0.787	-0.01 (-0.08, 0.06)	0.762	0.03 (-0.05, 0.11)	0.429	0.05 (-0.02, 0.11)	0.179
Current smoker	0.44 (-0.70, 1.59)	0.449	0.50 (-0.63, 1.63)	0.387	0.18 (-0.97, 1.33)	0.020	0.80 (-0.38, 1.98)	0.183	0.60 (-0.60, 1.80)	0.326
Ex-smoker	-0.64 (-1.72, 0.45)	0.250	-1.46 (-2.55, -0.36)	0.900	-0.68 (-1.77, 0.41)	0.223	-1.07 (-2.22, 0.08)	0.068	0.00 (-1.12, 1.12)	1.000
Never smoker	-0.48 (-1.56, 0.61)	0.390	-0.53 (-1.61, 0.55)	0.335	-0.24 (-1.32, 0.84)	0.661	-0.68 (-1.81, 0.45)	0.236	-0.96 (-2.11, 0.19)	0.103
Diabetes	-0.05 (-1.23, 1.12)	0.931	-0.73 (-1.91, 0.46)	0.228	-0.55 (-1.73, 0.63)	0.361	0.24 (-0.96, 1.44)	0.698	0.01 (-1.18, 1.19)	0.993
No Diabetes	-0.62 (-1.78, 0.54)	0.297	-0.76 (-1.93, 0.41)	0.204	-0.19 (-1.36, 0.99)	0.755	-1.19 (-2.38, 0.00)	0.051	-0.36 (-1.53, 0.81)	0.544
Hypertension	-0.12 (-1.38, 1.13)	0.848	-0.83 (-2.08, 0.43)	0.197	-0.08 (-1.35, 1.18)	0.897	-0.27 (-1.55, 1.00)	0.674	-0.37 (-1.62, 0.89)	0.564
No hypertension	-0.55 (-1.80, 0.71)	0.393	-0.66 (-1.91, 0.60)	0.304	-0.66 (-1.91, 0.60)	0.308	-0.68 (-1.95, 0.59)	0.295	0.01 (-1.24, 1.27)	0.985
Dyslipidaemia	-0.44 (-1.69, 0.81)	0.491	-0.34 (-1.60, 0.92)	0.598	-0.41 (-1.67, 0.84)	0.519	-0.80 (-2.07, 0.46)	0.214	-0.03 (-1.28, 1.23)	0.968
No Dyslipidaemia	-0.23 (-1.49, 1.03)	0.722	-1.15 (-2.42, 0.12)	0.077	-0.32 (-1.59, 0.94)	0.615	-0.15 (-1.42, 1.13)	0.819	-0.33 (-1.60, 0.93)	0.608
Previous MI	-1.38 (-2.69, -0.06)	0.040	-1.55 (-2.79, -0.30)	0.150	-1.36 (-2.68, -0.05)	0.420	-1.26 (-2.58, 0.07)	0.630	-0.38 (-1.60, 0.84)	0.542
No previous MI	0.71 (-0.60, 2.02)	0.290	0.06 (-1.18, 1.31)	0.924	0.63 (-0.69, 1.94)	0.350	0.30 (-1.02, 1.62)	0.652	0.02 (-1.20, 1.25)	0.970
Preserved LVF	0.25 (-0.94, 1.45)	0.679	0.42 (-0.79, 1.62)	0.499	0.37 (-0.82, 1.56)	0.541	0.68 (-0.56, 1.92)	0.285	-1.24 (-2.54, 0.06)	0.062
Mild LVSD	-1.31 (-2.53, -0.09)	0.036	-1.77 (-2.97, -0.56)	0.400	-1.30 (-2.52, -0.09)	0.350	-1.51 (-2.82, -0.20)	0.240	0.39 (-0.80, 1.59)	0.520
Moderate LVSD	0.45 (-0.79, 1.69)	0.479	1.42 (0.20, 2.64)	0.220	0.46 (-0.78, 1.70)	0.467	-0.82 (-2.16, 0.52)	0.228	0.32 (-0.96, 1.60)	0.623
Severe LVSD	-0.20 (-1.57, 1.18)	0.781	-0.54 (-1.91, 0.83)	0.438	-0.27 (-1.64, 1.10)	0.700	-0.41 (-1.77, 0.96)	0.557	0.19 (-1.11, 1.49)	0.777
No previous PCI	0.27 (-0.99, 1.54)	0.670	0.04 (-1.23, 1.31)	0.949	0.06 (-1.20, 1.31)	0.931	0.43 (-0.86, 1.71)	0.516	-0.45 (-1.71, 0.82)	0.490
Previous PCI	-0.94 (-2.20, 0.31)	0.142	-1.53 (-2.79, -0.26)	0.180	-0.79 (-2.04, 0.46)	0.214	-1.38 (-2.66, -0.09)	0.360	0.09 (-1.19, 1.37)	0.893
Artery LAD	0.65 (-0.43, 1.74)	0.240	1.18 (0.09, 2.27)	0.033	0.49 (-0.60, 1.58)	0.376	0.65 (-0.49, 1.78)	0.264	-0.21 (-1.30, 0.89)	0.710
Artery LCX	0.02 (-1.19, 1.23)	0.973	-0.53 (-1.75, 0.69)	0.395	-0.23 (-1.43, 0.98)	0.713	-0.21 (-1.45, 1.03)	0.741	0.33 (-0.85, 1.52)	0.579
Artery RCA	0.96 (-0.19, 2.11)	0.010	2.27 (1.10, 3.43)	<0.001	1.21 (0.06, 2.36)	0.040	0.25 (-0.95, 1.45)	0.001	-0.14 (-1.28, 1.00)	0.010
Artery DX	-1.18 (-2.51, 0.15)	0.082	-2.21 (-3.54, -0.88)	0.100	-0.89 (-2.23, 0.45)	0.195	-0.90 (-2.25, 0.44)	0.188	0.59 (-0.70, 1.88)	0.374
Artery LMS	-0.90 (-2.25, 0.45)	0.192	-1.62 (-2.97, -0.27)	0.190	-1.16 (-2.51, 0.19)	0.910	-0.34 (-1.71, 1.03)	0.624	-0.55 (-1.88, 0.77)	0.414
Artery OM	-0.23 (-1.60, 1.15)	0.748	-0.58 (-1.95, 0.78)	0.404	-0.17 (-1.54, 1.21)	0.813	-0.39 (-1.76, 0.97)	0.572	-0.38 (-1.69, 0.94)	0.577
Baseline pressure delta	-0.08 (-0.33, 0.16)	0.494	-0.02 (-0.26, 0.23)	0.900	-0.08 (-0.32, 0.16)	0.522	-0.12 (-0.40, 0.17)	0.418	-0.23 (-0.55, 0.08)	0.149
Hyperaemic Pa	0.00 (-0.80, 0.81)	0.992	-0.05 (-0.85, 0.76)	0.910	-0.01 (-0.82, 0.79)	0.971	0.02 (-0.78, 0.82)	0.961	-0.09 (-0.89, 0.71)	0.826
Hyperaemic Pd	0.05 (-0.76, 0.85)	0.911	0.08 (-0.72, 0.88)	0.848	0.06 (-0.75, 0.86)	0.891	0.02 (-0.79, 0.82)	0.969	0.05 (-0.75, 0.86)	0.897
Hyperaemic pressure delta	-0.04 (-0.85, 0.77)	0.919	-0.13 (-0.93, 0.68)	0.762	-0.07 (-0.88, 0.74)	0.864	0.00 (-0.80, 0.81)	0.992	-0.14 (-0.96, 0.67)	0.730
Vessel inlet diameter	-1.65 (-2.71, -0.58)	0.002	-1.80 (-2.86, -0.74)	0.001	-1.49 (-2.56, -0.42)	0.600	-0.83 (-1.95, 0.30)	0.151	-0.71 (-1.73, 0.30)	0.169
Vessel outlet diameter	-0.23 (-1.35, 0.88)	0.681	-0.74 (-1.85, 0.37)	0.192	-0.70 (-1.82, 0.43)	0.223	-0.80 (-1.98, 0.38)	0.185	0.82 (-0.34, 1.98)	0.168
Lesion diameter stenosis (%)	1.24 (-0.10, 2.58)	0.070	1.89 (0.55, 3.24)	0.006	1.30 (-0.04, 2.64)	0.058	1.40 (0.05, 2.75)	0.042	0.69 (-0.66, 2.04)	0.315
Total myocardial jeopardy	1.52 (0.23, 2.82)	0.020	3.30 (2.00, 4.61)	<0.001	2.39 (1.09, 3.68)	<0.001	1.37 (0.06, 2.68)	0.040	-0.79 (-2.10, 0.52)	0.003
Lesion specific jeopardy	-0.17 (-1.55, 1.21)	0.030	0.55 (-0.83, 1.93)	0.433	0.10 (-1.28, 1.48)	0.889	-0.09 (-1.47, 1.29)	0.898	-0.08 (-1.46, 1.31)	0.914
Haemoglobin	0.00 (-0.03, 0.04)	0.819	0.04 (0.00, 0.07)	0.028	0.01 (-0.02, 0.05)	0.531	-0.01 (-0.05, 0.02)	0.473	0.02 (-0.02, 0.07)	0.281
Haematocrit	0.16 (-1.22, 1.55)	0.817	0.44 (-0.95, 1.83)	0.533	0.24 (-1.15, 1.63)	0.734	0.26 (-1.12, 1.65)	0.710	0.05 (-1.33, 1.44)	0.939

Logistic regression predictors of 1D vFFR and FFR concordance. *MI, myocardial infarction; LVF, left ventricular function; LVSD, left ventricular systolic dysfunction; PCI, percutaneous coronary intervention; LAD, left anterior descending artery; LCX, left circumflex artery; RCA, right coronary artery; DX, diagonal branch; LMS, left main stem; OM, obtuse marginal branch; Pa, aortic pressure; Pd, distal pressure.*

Models	No-Leak		Homogenous		Anatomical		Conductance		Porosity	
Parameter	Log-odds (95% CI)	P-value	Log-odds (95% CI)	P-value	Log-odds (95% CI)	P-value	Log-odds (95% CI)	P-value	Log-odds (95% CI)	P-value
Male	0.03 (-1.21, 1.26)	0.968	0.24 (-1.01, 1.48)	0.709	-0.03 (-1.21, 1.14)	0.957	-0.09 (-1.27, 1.09)	0.878	0.60 (-0.61, 1.81)	0.334
Female	-0.38 (-1.62, 0.85)	0.543	-1.19 (-2.43, 0.05)	0.061	-0.71 (-1.88, 0.47)	0.239	-1.39 (-2.57, -0.21)	0.210	-1.27 (-2.47, -0.06)	0.039
Age	0.05 (-0.02, 0.11)	0.179	0.03 (-0.05, 0.11)	0.429	-0.01 (-0.08, 0.06)	0.762	0.01 (-0.05, 0.07)	0.787	-0.02 (-0.08, 0.05)	0.591
Current smoker	0.60 (-0.60, 1.80)	0.326	0.80 (-0.38, 1.98)	0.183	0.18 (-0.97, 1.33)	0.020	0.50 (-0.63, 1.63)	0.387	0.44 (-0.70, 1.59)	0.449
Ex-smoker	0.00 (-1.12, 1.12)	1.000	-1.07 (-2.22, 0.08)	0.068	-0.68 (-1.77, 0.41)	0.223	-1.46 (-2.55, -0.36)	0.900	-0.64 (-1.72, 0.45)	0.250
Never smoker	-0.96 (-2.11, 0.19)	0.103	-0.68 (-1.81, 0.45)	0.236	-0.24 (-1.32, 0.84)	0.661	-0.53 (-1.61, 0.55)	0.335	-0.48 (-1.56, 0.61)	0.390
Diabetes	0.01 (-1.18, 1.19)	0.993	0.24 (-0.96, 1.44)	0.698	-0.55 (-1.73, 0.63)	0.361	-0.73 (-1.91, 0.46)	0.228	-0.05 (-1.23, 1.12)	0.931
No diabetes	-0.36 (-1.53, 0.81)	0.544	-1.19 (-2.38, 0.00)	0.051	-0.19 (-1.36, 0.99)	0.755	-0.76 (-1.93, 0.41)	0.204	-0.62 (-1.78, 0.54)	0.297
Hypertension	-0.37 (-1.62, 0.89)	0.564	-0.27 (-1.55, 1.00)	0.674	-0.08 (-1.35, 1.18)	0.897	-0.83 (-2.08, 0.43)	0.197	-0.12 (-1.38, 1.13)	0.848
No hypertension	0.01 (-1.24, 1.27)	0.985	-0.68 (-1.95, 0.59)	0.295	-0.66 (-1.91, 0.60)	0.308	-0.66 (-1.91, 0.60)	0.304	-0.55 (-1.80, 0.71)	0.393
Dyslipidaemia	-0.03 (-1.28, 1.23)	0.968	-0.80 (-2.07, 0.46)	0.214	-0.41 (-1.67, 0.84)	0.519	-0.34 (-1.60, 0.92)	0.598	-0.44 (-1.69, 0.81)	0.491
No dyslipidaemia	-0.33 (-1.60, 0.93)	0.608	-0.15 (-1.42, 1.13)	0.819	-0.32 (-1.59, 0.94)	0.615	-1.15 (-2.42, 0.12)	0.077	-0.23 (-1.49, 1.03)	0.722
Previous MI	-0.38 (-1.60, 0.84)	0.542	-1.26 (-2.58, 0.07)	0.630	-1.36 (-2.68, -0.05)	0.420	-1.55 (-2.79, -0.30)	0.150	-1.38 (-2.69, -0.06)	0.040
No previous MI	0.02 (-1.20, 1.25)	0.970	0.30 (-1.02, 1.62)	0.652	0.63 (-0.69, 1.94)	0.350	0.06 (-1.18, 1.31)	0.924	0.71 (-0.60, 2.02)	0.290
Preserved LVF	-1.24 (-2.54, 0.06)	0.062	0.68 (-0.56, 1.92)	0.285	0.37 (-0.82, 1.56)	0.541	0.42 (-0.79, 1.62)	0.499	0.25 (-0.94, 1.45)	0.679
Mild LVSD	0.39 (-0.80, 1.59)	0.520	-1.51 (-2.82, -0.20)	0.240	-1.30 (-2.52, -0.09)	0.350	-1.77 (-2.97, -0.56)	0.400	-1.31 (-2.53, -0.09)	0.036
Moderate LVSD	0.32 (-0.96, 1.60)	0.623	-0.82 (-2.16, 0.52)	0.228	0.46 (-0.78, 1.70)	0.467	1.42 (0.20, 2.64)	0.220	0.45 (-0.79, 1.69)	0.479
Severe LVSD	0.19 (-1.11, 1.49)	0.777	-0.41 (-1.77, 0.96)	0.557	-0.27 (-1.64, 1.10)	0.700	-0.54 (-1.91, 0.83)	0.438	-0.20 (-1.57, 1.18)	0.781
No previous PCI	-0.45 (-1.71, 0.82)	0.490	0.43 (-0.86, 1.71)	0.516	0.06 (-1.20, 1.31)	0.931	0.04 (-1.23, 1.31)	0.949	0.27 (-0.99, 1.54)	0.670
Previous PCI	0.09 (-1.19, 1.37)	0.893	-1.38 (-2.66, -0.09)	0.360	-0.79 (-2.04, 0.46)	0.214	-1.53 (-2.79, -0.26)	0.180	-0.94 (-2.20, 0.31)	0.142
Artery LAD	-0.21 (-1.30, 0.89)	0.710	0.65 (-0.49, 1.78)	0.264	0.49 (-0.60, 1.58)	0.376	1.18 (0.09, 2.27)	0.033	0.65 (-0.43, 1.74)	0.240
Artery LCX	0.33 (-0.85, 1.52)	0.579	-0.21 (-1.45, 1.03)	0.741	-0.23 (-1.43, 0.98)	0.713	-0.53 (-1.75, 0.69)	0.395	0.02 (-1.19, 1.23)	0.973
Artery RCA	-0.14 (-1.28, 1.00)	0.010	0.25 (-0.95, 1.45)	0.001	1.21 (0.06, 2.36)	0.040	2.27 (1.10, 3.43)	<0.001	0.96 (-0.19, 2.11)	0.010
Artery DX	0.59 (-0.70, 1.88)	0.374	-0.90 (-2.25, 0.44)	0.188	-0.89 (-2.23, 0.45)	0.195	-2.21 (-3.54, -0.88)	0.100	-1.18 (-2.51, 0.15)	0.082
Artery LMS	-0.55 (-1.88, 0.77)	0.414	-0.34 (-1.71, 1.03)	0.624	-1.16 (-2.51, 0.19)	0.910	-1.62 (-2.97, -0.27)	0.190	-0.90 (-2.25, 0.45)	0.192
Artery OM	-0.38 (-1.69, 0.94)	0.577	-0.39 (-1.76, 0.97)	0.572	-0.17 (-1.54, 1.21)	0.813	-0.58 (-1.95, 0.78)	0.404	-0.23 (-1.60, 1.15)	0.748
Baseline pressure delta	-0.23 (-0.55, 0.08)	0.149	-0.12 (-0.40, 0.17)	0.418	-0.08 (-0.32, 0.16)	0.522	-0.02 (-0.26, 0.23)	0.900	-0.08 (-0.33, 0.16)	0.494
Hyperaemic Pa	-0.09 (-0.89, 0.71)	0.826	0.02 (-0.78, 0.82)	0.961	-0.01 (-0.82, 0.79)	0.971	-0.05 (-0.85, 0.76)	0.910	0.00 (-0.80, 0.81)	0.992
Hyperaemic Pd	0.05 (-0.75, 0.86)	0.897	0.02 (-0.79, 0.82)	0.969	0.06 (-0.75, 0.86)	0.891	0.08 (-0.72, 0.88)	0.848	0.05 (-0.76, 0.85)	0.911
Hyperaemic pressure delta	-0.14 (-0.96, 0.67)	0.730	0.00 (-0.80, 0.81)	0.992	-0.07 (-0.88, 0.74)	0.864	-0.13 (-0.93, 0.68)	0.762	-0.04 (-0.85, 0.77)	0.919
Vessel inlet diameter	-0.71 (-1.73, 0.30)	0.169	-0.83 (-1.95, 0.30)	0.151	-1.49 (-2.56, -0.42)	0.600	-1.80 (-2.86, -0.74)	0.001	-1.65 (-2.71, -0.58)	0.002
Vessel outlet diameter	0.82 (-0.34, 1.98)	0.168	-0.80 (-1.98, 0.38)	0.185	-0.70 (-1.82, 0.43)	0.223	-0.74 (-1.85, 0.37)	0.192	-0.23 (-1.35, 0.88)	0.681
Lesion diameter stenosis (%)	0.69 (-0.66, 2.04)	0.315	1.40 (0.05, 2.75)	0.042	1.30 (-0.04, 2.64)	0.058	1.89 (0.55, 3.24)	0.006	1.24 (-0.10, 2.58)	0.070
Total myocardial jeopardy	-0.79 (-2.10, 0.52)	0.003	1.37 (0.06, 2.68)	0.040	2.39 (1.09, 3.68)	<0.001	3.30 (2.00, 4.61)	<0.001	1.52 (0.23, 2.82)	0.020
Lesion specific jeopardy	-0.08 (-1.46, 1.31)	0.914	-0.09 (-1.47, 1.29)	0.898	0.10 (-1.28, 1.48)	0.889	0.55 (-0.83, 1.93)	0.433	-0.17 (-1.55, 1.21)	0.030
Haemoglobin	0.02 (-0.02, 0.07)	0.281	-0.01 (-0.05, 0.02)	0.473	0.01 (-0.02, 0.05)	0.531	0.04 (0.00, 0.07)	0.028	0.00 (-0.03, 0.04)	0.819
Haematocrit	0.05 (-1.33, 1.44)	0.939	0.26 (-1.12, 1.65)	0.710	0.24 (-1.15, 1.63)	0.734	0.44 (-0.95, 1.83)	0.533	0.16 (-1.22, 1.55)	0.817

Logistic regression predictors of false positive 1D vFFR result. *MI, myocardial infarction; LVF, left ventricular function; LVSD, left ventricular systolic dysfunction; PCI, percutaneous coronary intervention; LAD, left anterior descending artery; LCX, left circumflex artery; RCA, right coronary artery; DX, diagonal branch; LMS, left main stem; OM, obtuse marginal branch; Pa, aortic pressure; Pd, distal pressure.*

Models	No-Leak		Homogenous		Anatomical		Conductance		Porosity	
Parameter	Log-odds (95% CI)	P-value	Log-odds (95% CI)	P-value	Log-odds (95% CI)	P-value	Log-odds (95% CI)	P-value	Log-odds (95% CI)	P-value
Male	0.77 (-0.48, 2.03)	0.226	0.83 (-0.41, 2.07)	0.191	0.84 (-0.43, 2.11)	0.196	0.27 (-0.93, 1.47)	0.663	-0.18 (-1.39, 1.02)	0.765
Female	-1.20 (-2.44, 0.05)	0.030	-0.67 (-1.90, 0.56)	0.287	-1.19 (-2.45, 0.07)	0.065	-0.31 (-1.50, 0.89)	0.613	0.31 (-0.89, 1.51)	0.611
Age	0.01 (-0.06, 0.07)	0.872	-0.01 (-0.07, 0.06)	0.820	-0.02 (-0.09, 0.05)	0.500	-0.05 (-0.12, 0.01)	0.104	0.02 (-0.05, 0.09)	0.539
Current smoker	0.73 (-0.45, 1.92)	0.225	0.47 (-0.72, 1.65)	0.442	0.97 (-0.23, 2.17)	0.113	-0.24 (-1.42, 0.94)	0.691	0.43 (-0.76, 1.61)	0.482
Ex-smoker	-0.57 (-1.67, 0.52)	0.305	-0.12 (-1.23, 1.00)	0.837	-0.57 (-1.69, 0.56)	0.325	0.04 (-1.04, 1.11)	0.943	0.52 (-0.58, 1.63)	0.354
Never smoker	-0.58 (-1.70, 0.53)	0.303	-0.19 (-1.31, 0.92)	0.737	-0.75 (-1.89, 0.39)	0.196	0.16 (-0.92, 1.24)	0.773	-0.82 (-1.96, 0.32)	0.157
Diabetes	0.45 (-0.73, 1.63)	0.457	0.29 (-0.92, 1.50)	0.641	0.88 (-0.34, 2.10)	0.158	0.68 (-0.48, 1.85)	0.248	0.24 (-0.95, 1.43)	0.692
No diabetes	-0.67 (-1.86, 0.51)	0.265	-0.03 (-1.25, 1.18)	0.960	-1.11 (-2.33, 0.11)	0.075	-0.13 (-1.30, 1.04)	0.823	-0.25 (-1.46, 0.95)	0.681
Hypertension	-0.13 (-1.39, 1.13)	0.842	0.27 (-0.99, 1.54)	0.675	-0.01 (-1.28, 1.25)	0.982	0.12 (-1.13, 1.37)	0.851	0.44 (-0.81, 1.69)	0.492
No hypertension	-0.30 (-1.55, 0.96)	0.643	-0.11 (-1.37, 1.15)	0.861	-0.34 (-1.60, 0.93)	0.604	-0.16 (-1.40, 1.08)	0.799	-0.31 (-1.56, 0.93)	0.622
Dyslipidaemia	-0.27 (-1.52, 0.97)	0.665	0.07 (-1.18, 1.33)	0.907	-0.12 (-1.38, 1.13)	0.847	0.05 (-1.18, 1.29)	0.933	-0.22 (-1.46, 1.03)	0.733
No dyslipidaemia	-0.15 (-1.41, 1.11)	0.816	0.08 (-1.18, 1.35)	0.897	-0.23 (-1.50, 1.05)	0.727	-0.10 (-1.35, 1.16)	0.882	0.34 (-0.91, 1.60)	0.593
Previous MI	-1.03 (-2.22, 0.16)	0.010	-0.21 (-1.40, 0.97)	0.006	-0.67 (-1.87, 0.53)	0.020	-0.27 (-1.43, 0.90)	0.004	0.17 (-1.01, 1.36)	0.040
No previous MI	0.60 (-0.60, 1.81)	0.325	0.37 (-0.83, 1.57)	0.543	0.32 (-0.89, 1.53)	0.605	0.23 (-0.95, 1.40)	0.706	-0.05 (-1.25, 1.15)	0.937
Preserved LVF	0.62 (-0.54, 1.78)	0.295	0.29 (-0.90, 1.49)	0.629	1.02 (-0.19, 2.22)	0.099	-0.83 (-2.01, 0.35)	0.168	-1.43 (-2.64, -0.23)	0.019
Mild LVSD	0.14 (-1.10, 1.39)	0.821	-0.63 (-1.95, 0.69)	0.349	-0.58 (-1.93, 0.77)	0.399	-0.31 (-1.55, 0.92)	0.620	1.43 (0.18, 2.67)	0.025
Moderate LVSD	0.05 (-1.22, 1.33)	0.936	0.12 (-1.15, 1.39)	0.855	-1.10 (-2.43, 0.23)	0.104	0.56 (-0.66, 1.78)	0.369	2.13 (0.85, 3.40)	0.001
Severe LVSD	-1.02 (-2.35, 0.32)	0.134	-0.35 (-1.70, 1.01)	0.615	-0.60 (-1.96, 0.75)	0.381	-0.64 (-1.99, 0.72)	0.356	-1.25 (-2.59, 0.09)	0.067
No previous PCI	-1.05 (-2.28, 0.17)	0.092	-0.50 (-1.72, 0.72)	0.421	-0.51 (-1.75, 0.74)	0.424	-1.41 (-2.63, -0.19)	0.024	-1.44 (-2.69, -0.19)	0.024
Previous PCI	0.63 (-0.61, 1.87)	0.319	0.66 (-0.57, 1.89)	0.293	0.16 (-1.10, 1.41)	0.808	1.37 (0.15, 2.59)	0.028	1.56 (0.33, 2.80)	0.013
Artery LAD	-0.16 (-1.23, 0.90)	0.767	0.04 (-1.04, 1.13)	0.938	-0.31 (-1.43, 0.80)	0.579	0.15 (-0.91, 1.20)	0.787	0.35 (-0.75, 1.46)	0.528
Artery LCX	1.65 (0.46, 2.84)	0.006	1.04 (-0.15, 2.23)	0.003	1.89 (0.68, 3.10)	0.002	0.40 (-0.80, 1.59)	0.010	0.04 (-1.20, 1.27)	0.001
Artery RCA	-0.49 (-1.64, 0.65)	0.400	0.16 (-0.99, 1.31)	0.788	-0.63 (-1.80, 0.55)	0.295	-0.11 (-1.24, 1.01)	0.842	0.40 (-0.77, 1.57)	0.499
Artery DX	-0.31 (-1.67, 1.06)	0.658	-0.21 (-1.58, 1.15)	0.758	-0.61 (-1.96, 0.74)	0.376	-0.37 (-1.74, 0.99)	0.592	-0.05 (-1.43, 1.33)	0.943
Artery LMS	-0.22 (-1.55, 1.12)	0.749	-0.42 (-1.77, 0.94)	0.547	0.12 (-1.25, 1.48)	0.868	1.23 (-0.09, 2.55)	0.040	-0.20 (-1.57, 1.18)	0.780
Artery OM	-0.90 (-2.24, 0.44)	0.188	-0.45 (-1.79, 0.89)	0.508	-0.81 (-2.15, 0.54)	0.239	-1.32 (-2.66, 0.01)	0.052	-0.42 (-1.78, 0.94)	0.544
Baseline pressure delta	-0.10 (-0.27, 0.07)	0.247	-0.23 (-0.42, -0.05)	0.010	-0.25 (-0.44, -0.06)	0.110	-0.01 (-0.16, 0.15)	0.916	-0.28 (-0.48, -0.07)	0.007
Hyperaemic Pa	0.04 (-0.77, 0.84)	0.930	0.08 (-0.73, 0.88)	0.854	0.08 (-0.72, 0.88)	0.841	0.03 (-0.77, 0.83)	0.937	0.09 (-0.71, 0.89)	0.823
Hyperaemic Pd	-0.05 (-0.86, 0.75)	0.896	-0.07 (-0.88, 0.73)	0.860	-0.11 (-0.92, 0.69)	0.783	-0.01 (-0.81, 0.79)	0.983	-0.11 (-0.92, 0.69)	0.788
Hyperaemic pressure delta	0.09 (-0.72, 0.90)	0.828	0.15 (-0.66, 0.96)	0.720	0.20 (-0.61, 1.00)	0.636	0.04 (-0.76, 0.85)	0.920	0.20 (-0.61, 1.01)	0.625
Vessel inlet diameter	0.13 (-0.87, 1.14)	0.793	-0.25 (-1.27, 0.77)	0.630	0.20 (-0.86, 1.25)	0.712	-1.14 (-2.13, -0.16)	0.063	-1.26 (-2.29, -0.23)	0.016
Vessel outlet diameter	0.80 (-0.37, 1.96)	0.181	0.42 (-0.77, 1.61)	0.489	0.99 (-0.24, 2.22)	0.040	0.39 (-0.73, 1.50)	0.498	-0.66 (-1.87, 0.55)	0.286
Lesion diameter stenosis (%)	-0.54 (-1.89, 0.81)	0.434	0.01 (-1.34, 1.36)	0.984	-0.35 (-1.71, 1.00)	0.611	-2.76 (-4.11, -1.42)	<0.001	-1.33 (-2.69, 0.02)	0.054
Total myocardial jeopardy	-0.12 (-1.42, 1.18)	0.857	0.94 (-0.38, 2.25)	0.162	0.40 (-0.92, 1.72)	0.550	1.57 (0.28, 2.86)	0.087	-0.09 (-1.39, 1.22)	0.896
Lesion specific jeopardy	0.67 (-0.71, 2.05)	0.340	0.53 (-0.85, 1.91)	0.454	0.54 (-0.84, 1.92)	0.443	1.39 (0.01, 2.76)	0.049	0.68 (-0.70, 2.05)	0.337
Haemoglobin	-0.03 (-0.07, 0.01)	0.162	-0.05 (-0.09, 0.00)	0.042	-0.02 (-0.07, 0.02)	0.311	0.00 (-0.03, 0.04)	0.814	0.00 (-0.04, 0.04)	0.989
Haematocrit	0.06 (-1.33, 1.44)	0.935	0.09 (-1.29, 1.48)	0.894	0.12 (-1.27, 1.51)	0.866	0.33 (-1.06, 1.71)	0.642	-0.10 (-1.49, 1.28)	0.887

Logistic regression predictors of false negative 1D vFFR result. *MI, myocardial infarction; LVF, left ventricular function; LVSD, left ventricular systolic dysfunction; PCI, percutaneous coronary intervention; LAD, left anterior descending artery; LCX, left circumflex artery; RCA, right coronary artery; DX, diagonal branch; LMS, left main stem; OM, obtuse marginal branch; Pa, aortic pressure; Pd, distal pressure.*

Supplementary 10 Results for patient-specific viscosity simulations

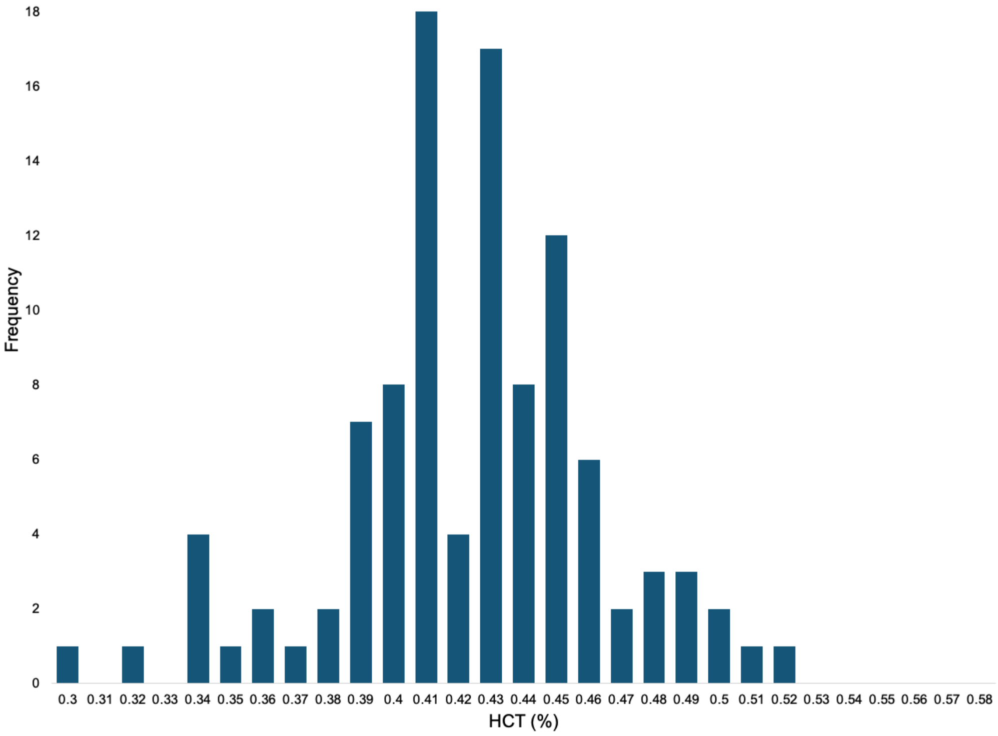

S10 figure 1. Distribution of HCT values for all included patients

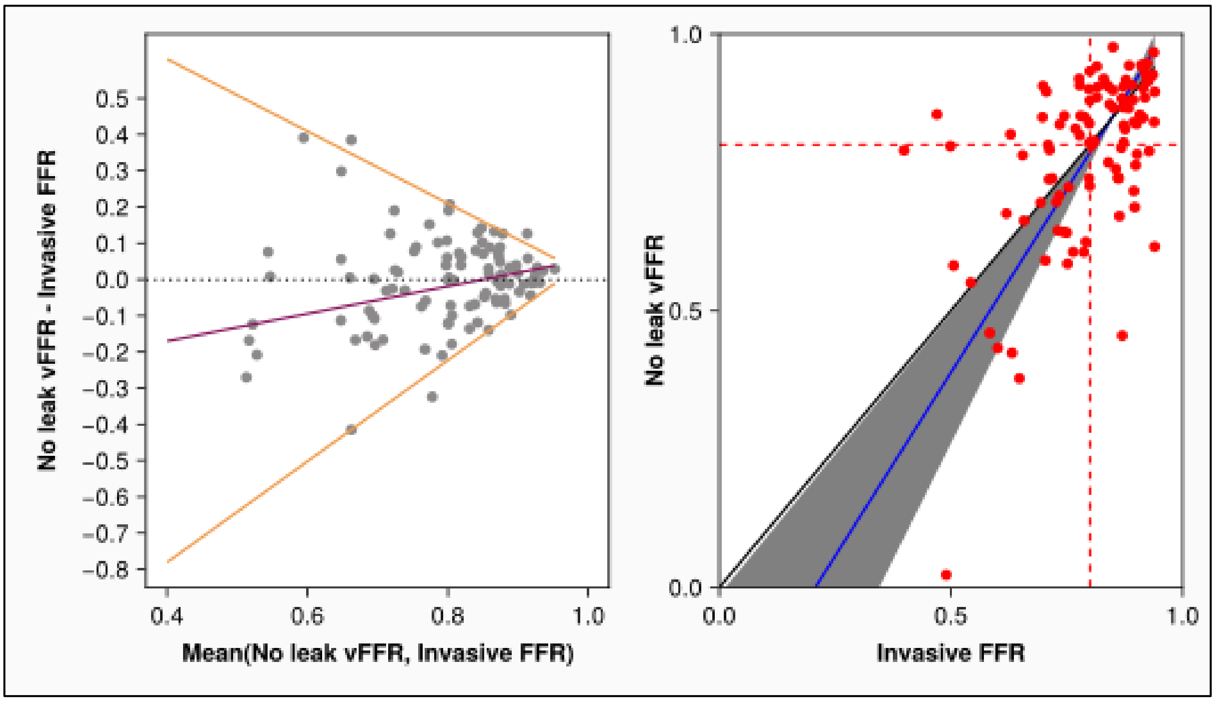

S10 figure 2. Bland Altman and Passing and Bablok regression comparing invasive FFR with no leak, personalised viscosity vFFR. At the diagnostic threshold of 0.80 no leak vFFR underestimated FFR by -0.02 (95% LOA -0.23 to 0.20).

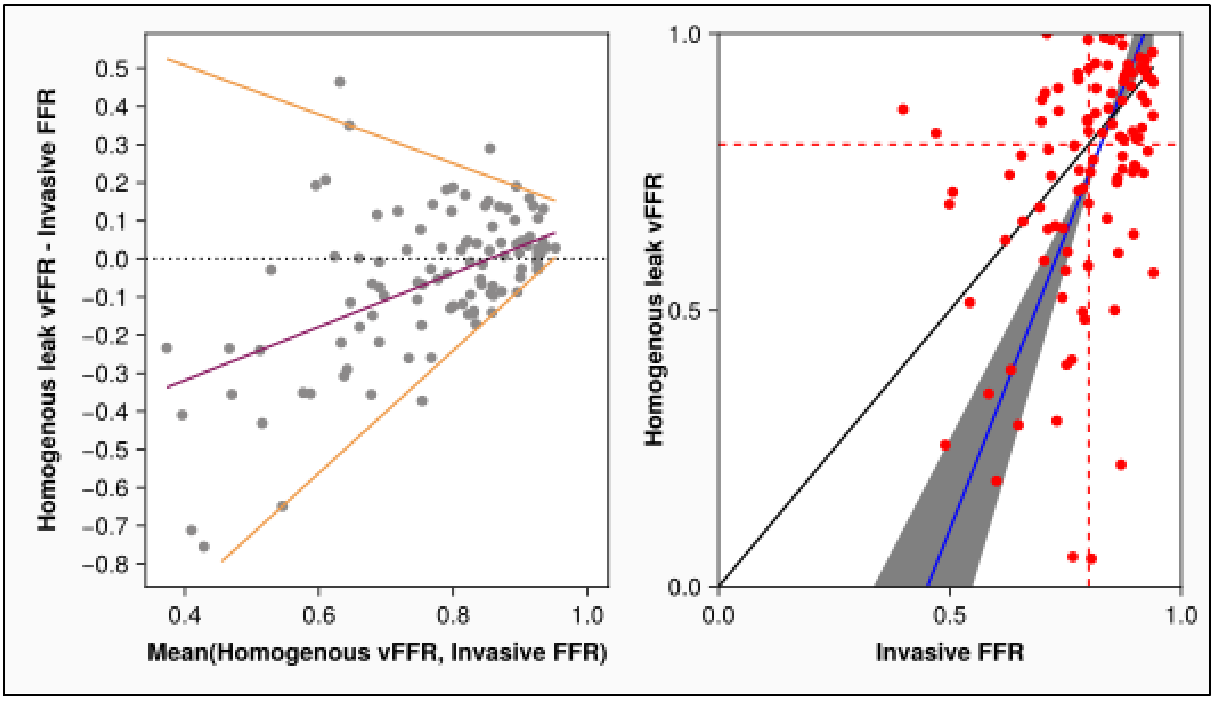

S10 figure 3. Bland Altman and Passing and Bablok regression comparing invasive FFR with homogenous leak, personalised viscosity vFFR. At the diagnostic threshold of 0.80 homogenous leak vFFR underestimated FFR by -0.04 (95% LOA -0.23 to 0.25).

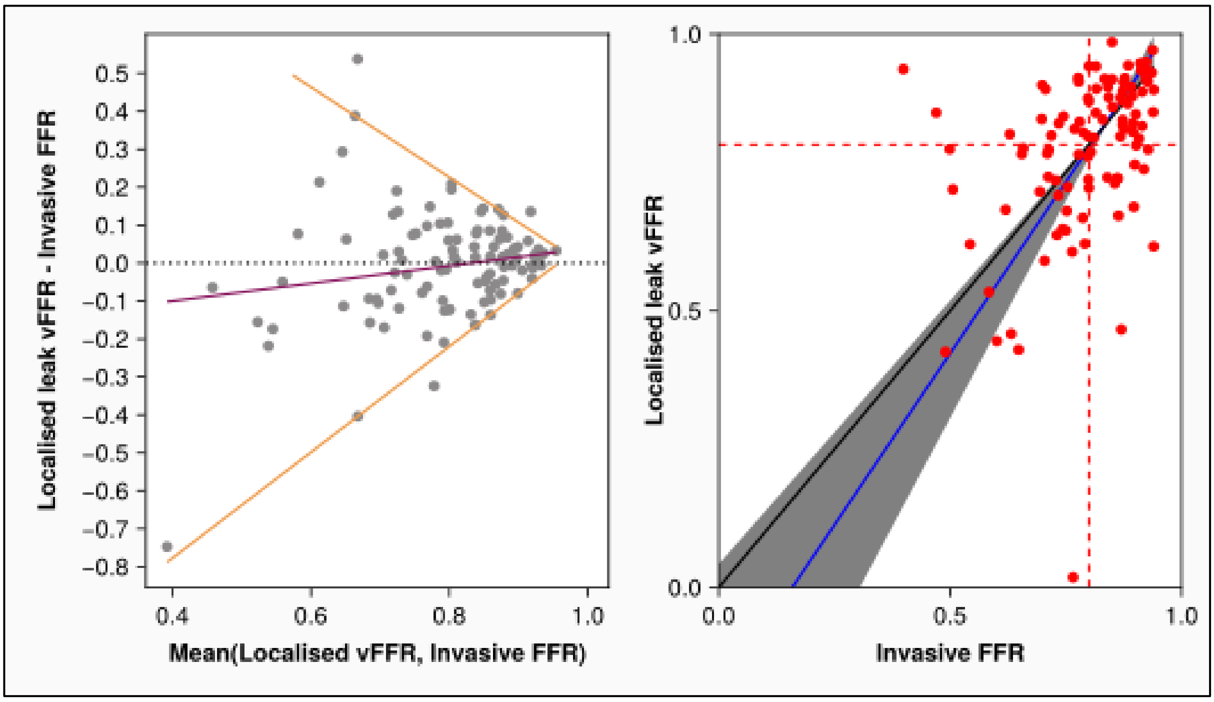

S10 figure 4. Bland Altman and Passing and Bablok regression comparing invasive FFR with localised leak, personalised viscosity vFFR. At the diagnostic threshold of 0.80 localised leak vFFR exhibited zero overall bias versus FFR (95% LOA -0.23 to 0.22).

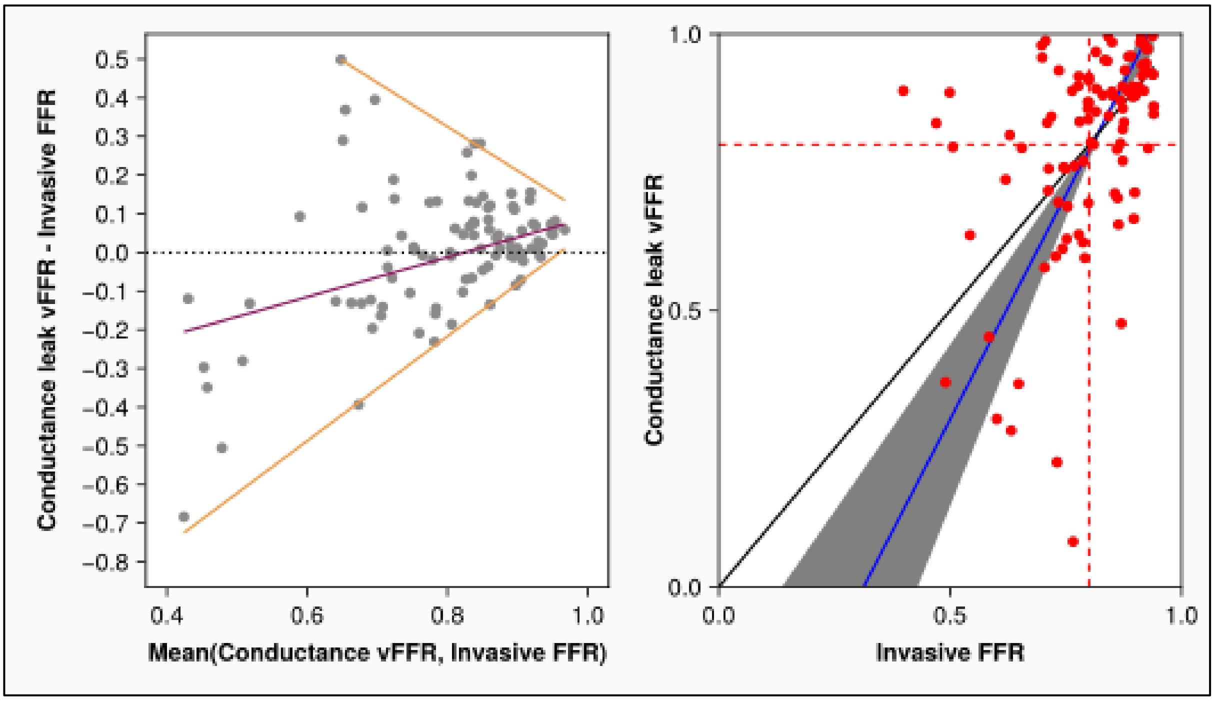

S10 figure 5. Bland Altman and Passing and Bablok regression comparing invasive FFR with conductance leak, personalised viscosity vFFR. At the diagnostic threshold of 0.80 conductance leak vFFR underestimated FFR by -0.01 (95% LOA -0.21 to 0.32).

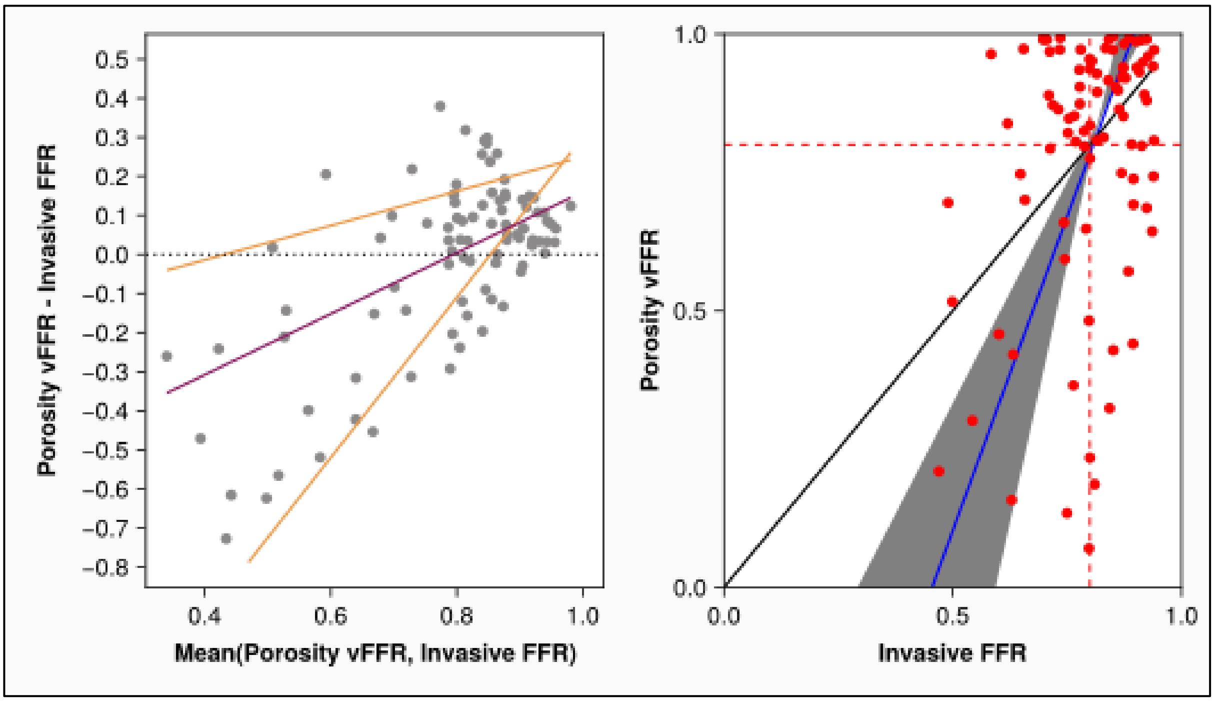

S10 figure 6. Bland Altman and Passing and Bablok regression comparing invasive FFR with porosity leak, personalised viscosity vFFR. At the diagnostic threshold of 0.80 porosity leak vFFR exhibited zero overall bias versus FFR (95% LOA -0.23 to 0.25).

Supplementary 10 Table 1: 50th, 2.5th and 97.5th quantile regression lines for Bland-Altman plots

	Median quantile	2.5th quantile	97.5th quantile
No leak	y = 0.38x – 0.32	y = 1.39x – 1.34	y = -1.00x + 1.00
Homogenous leak	y = 0.70x – 0.60	y = 1.61x – 1.52	y = -0.64x + 0.76
Localised leak	y = 0.23x – 0.19	y = 1.39x – 1.34	y = -1.19x + 1.17
Conductance leak	y = 0.51x – 0.42	y = 1.35x – 1.29	y = -1.14x + 1.23
Porosity leak	y = 0.78x – 0.62	y = 2.05x – 1.75	y = -0.19 + 0.44

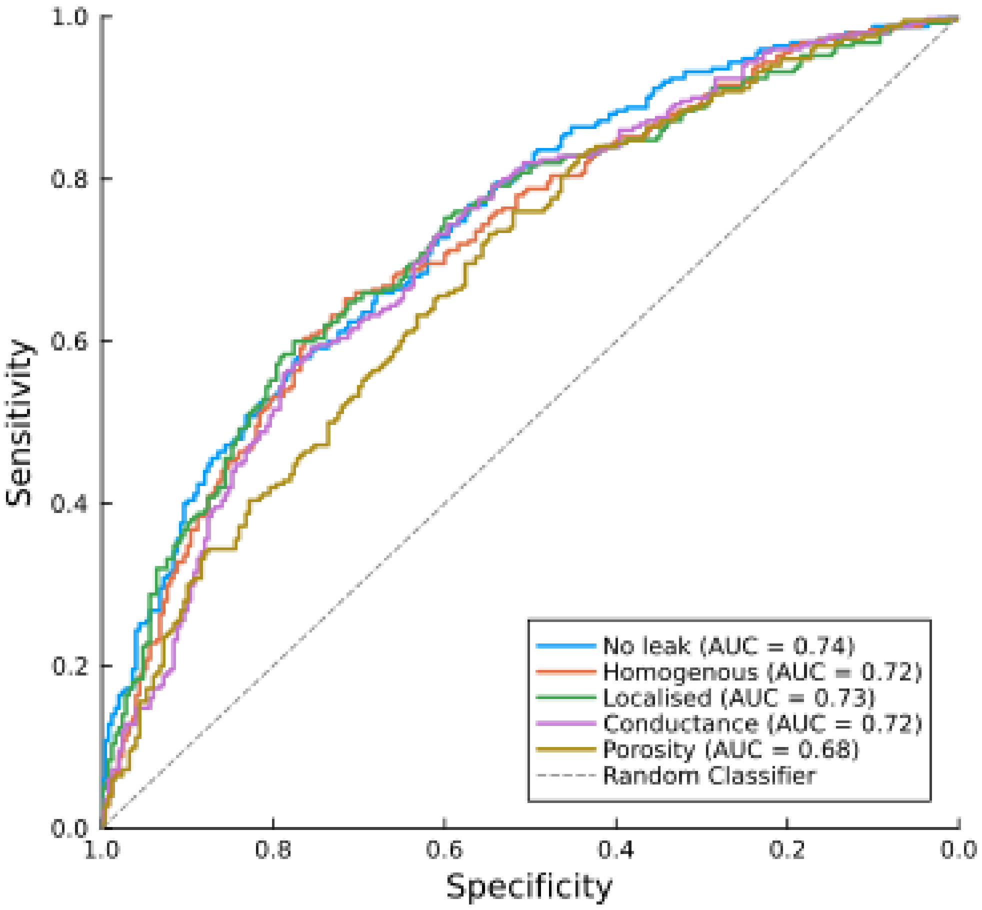

S10 figure 7: ROC curves for patient-specific viscosity simulations

Supplementary 11 agreement and diagnostic accuracy focal versus diffuse disease

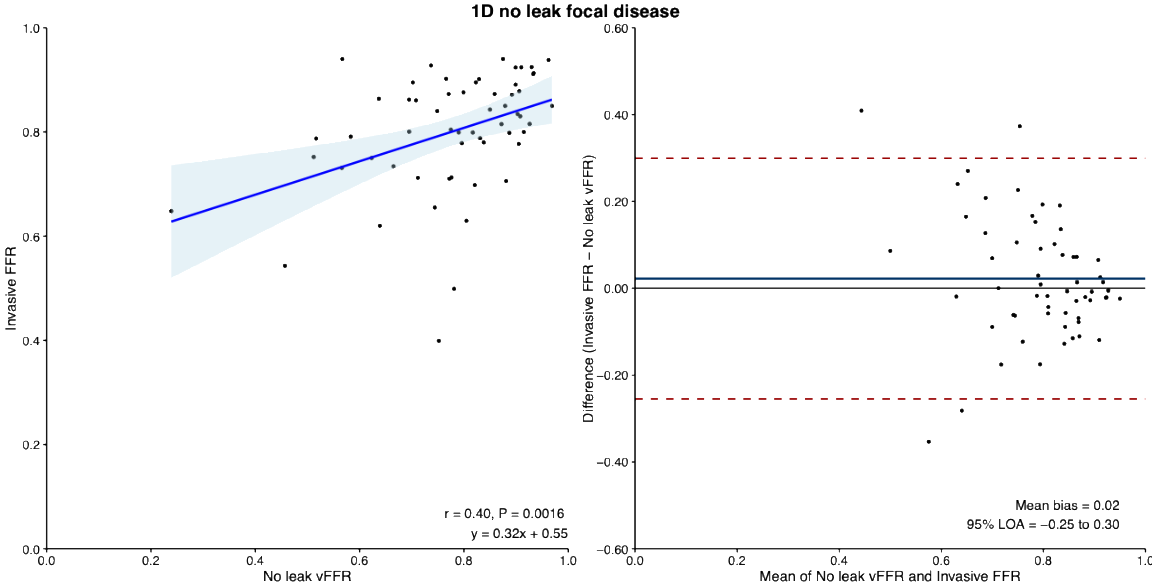

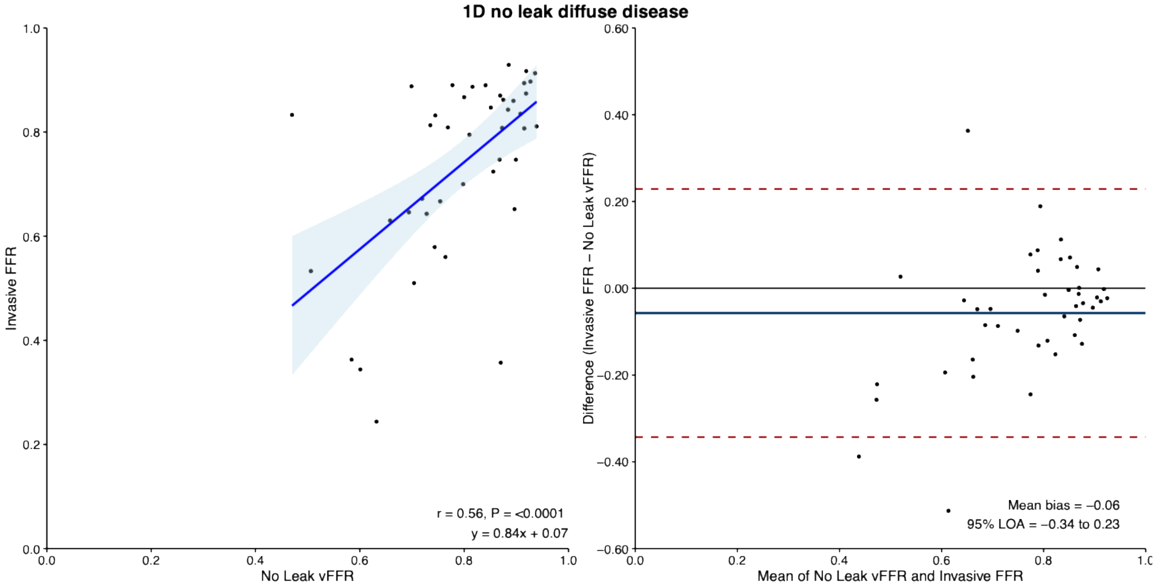

	No leak Focal Disease		No leak Diffuse Disease	
**Statistic**	**Value**	**95% CI**	**Value**	**95% CI**
Sensitivity	65.38%	44.33% to 82.79%	65.00%	40.78% to 84.61%
Specificity	62.50%	43.69% to 78.90%	73.91%	51.59% to 89.77%
Positive Likelihood Ratio	1.74	1.03 to 2.95	2.49	1.17 to 5.32
Negative Likelihood Ratio	0.55	0.31 to 1.00	0.47	0.25 to 0.90
Disease prevalence (*)	44.83%	31.74% to 58.46%	46.51%	31.18% to 62.35%
Positive Predictive Value (*)	58.62%	45.53% to 70.60%	68.42%	50.35% to 82.24%
Negative Predictive Value (*)	68.97%	55.13% to 80.08%	70.83%	56.03% to 82.23%

Accuracy (*)	63.79%	50.12% to 76.01%	69.77%	53.87% to 82.82%

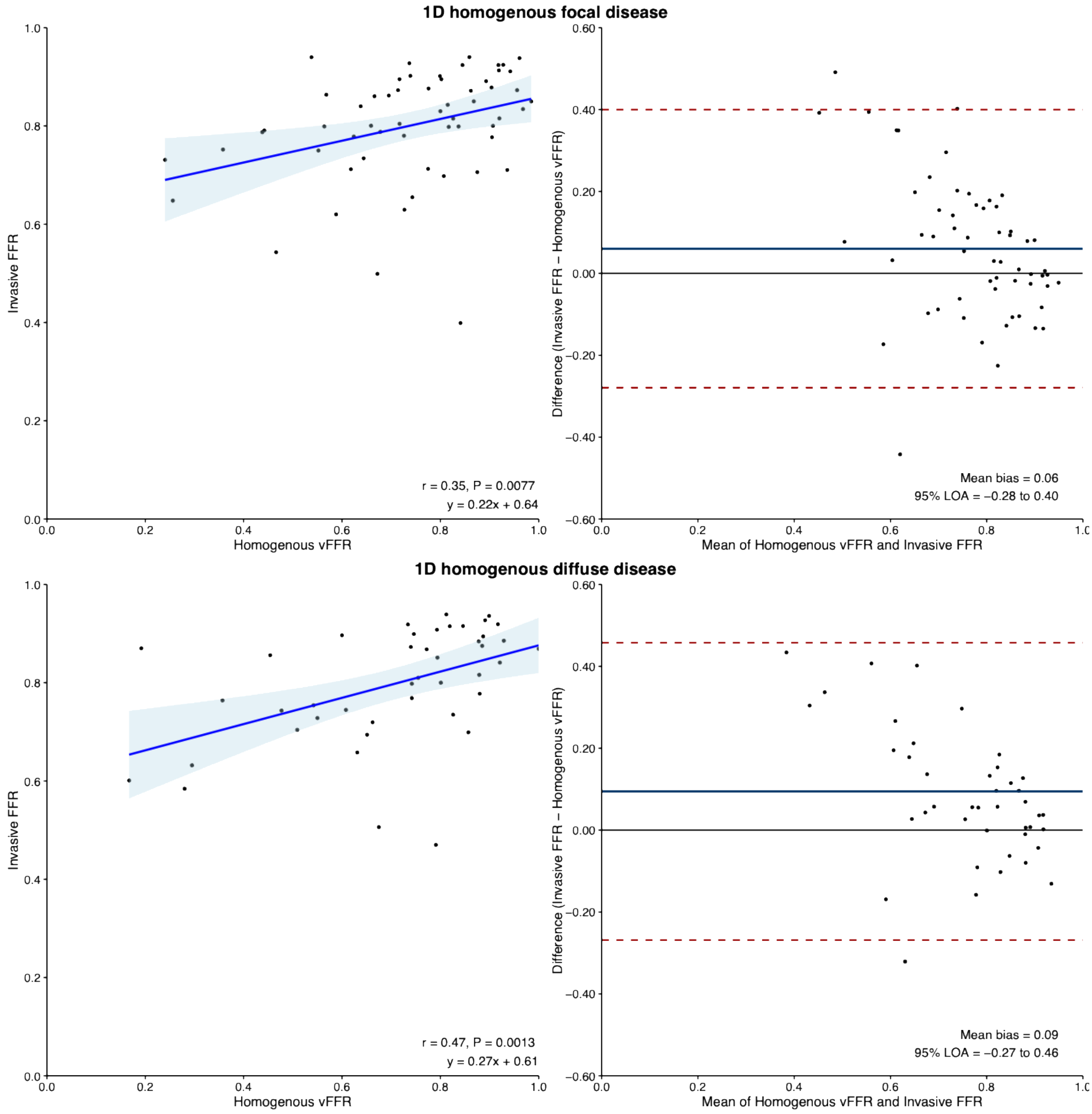

	Homogenous Focal Disease		Homogenous Diffuse Disease	
**Statistic**	**Value**	**95% CI**	**Value**	**95% CI**
Sensitivity	69.23%	48.21% to 85.67%	80.00%	56.34% to 94.27%
Specificity	56.25%	37.66% to 73.64%	56.52%	34.49% to 76.81%
Positive Likelihood Ratio	1.58	0.99 to 2.53	1.84	1.10 to 3.08
Negative Likelihood Ratio	0.55	0.28 to 1.05	0.35	0.14 to 0.91
Disease prevalence (*)	44.83%	31.74% to 58.46%	46.51%	31.18% to 62.35%
Positive Predictive Value (*)	56.25%	44.58% to 67.27%	61.54%	48.88% to 72.81%

Negative Predictive Value (*)	69.23%	53.95% to 81.21%	76.47%	55.77% to 89.34%
Accuracy (*)	62.07%	48.37% to 74.49%	67.44%	51.46% to 80.92%

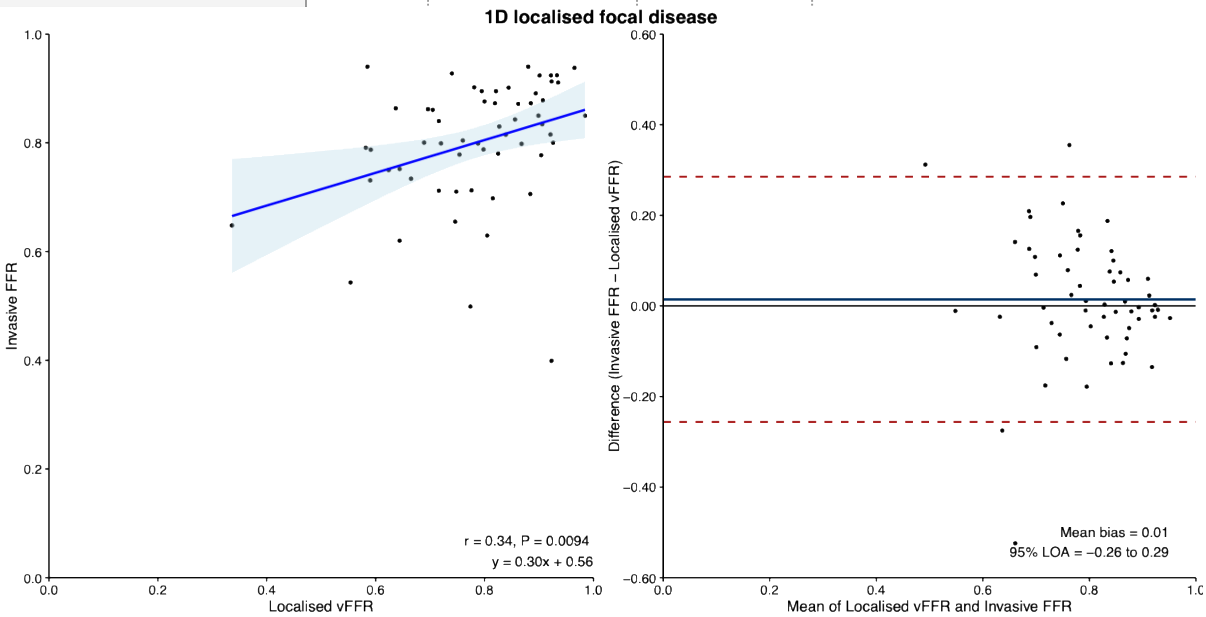

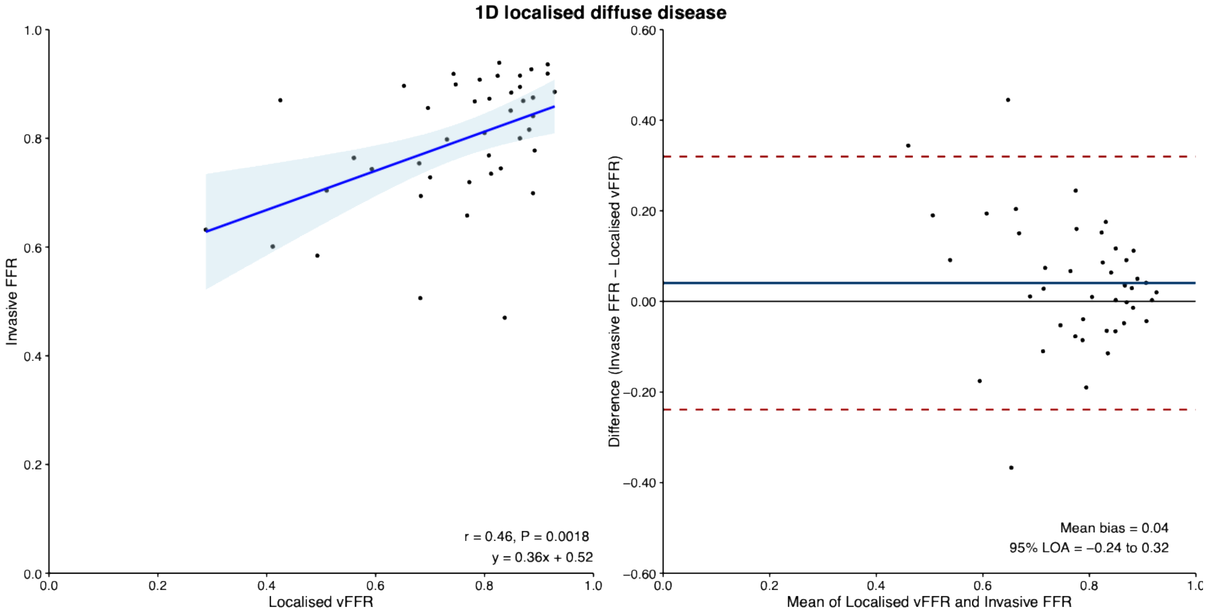

	**Localised Focal Disease**		**Localised Diffuse Disease**	
**Statistic**	**Value**	**95% CI**	**Value**	**95% CI**
Sensitivity	69.23%	48.21% to 85.67%	65.00%	40.78% to 84.61%
Specificity	65.62%	46.81% to 81.43%	65.22%	42.73% to 83.62%
Positive Likelihood Ratio	2.01	1.17 to 3.47	1.87	0.98 to 3.56
Negative Likelihood Ratio	0.47	0.25 to 0.88	0.54	0.28 to 1.05
Disease prevalence (*)	44.83%	31.74% to 58.46%	46.51%	31.18% to 62.35%

Positive Predictive Value (*)	62.07%	48.74% to 73.80%	61.90%	46.01% to 75.60%
Negative Predictive Value (*)	72.41%	58.33% to 83.12%	68.18%	52.36% to 80.69%
Accuracy (*)	67.24%	53.66% to 78.99%	65.12%	49.07% to 78.99%

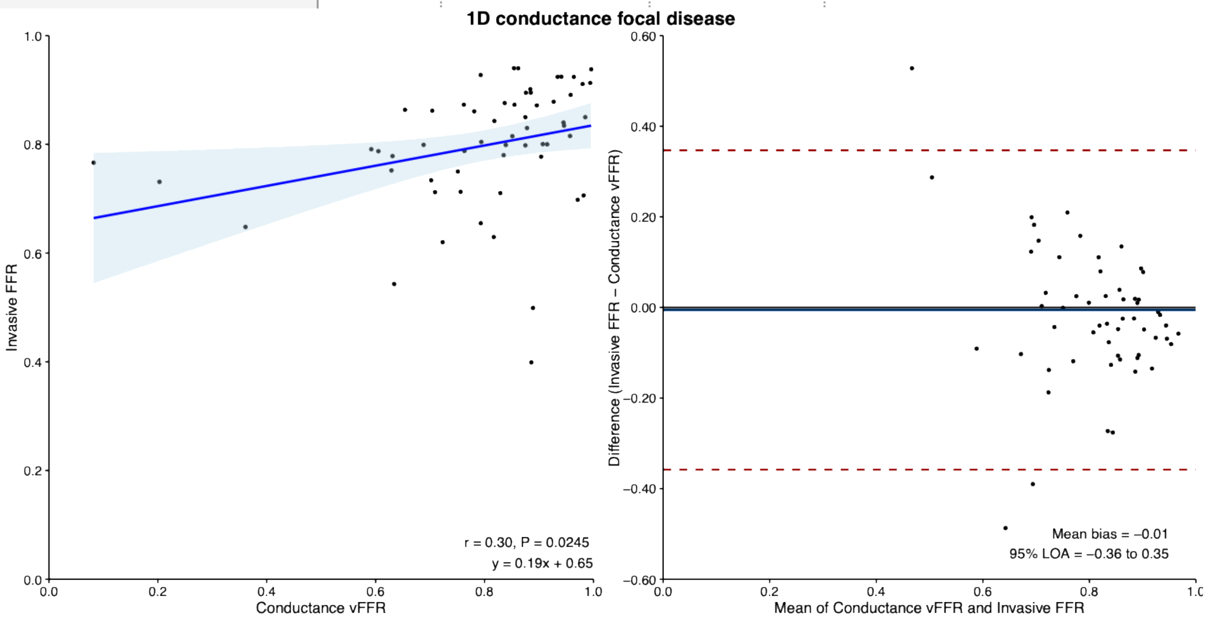

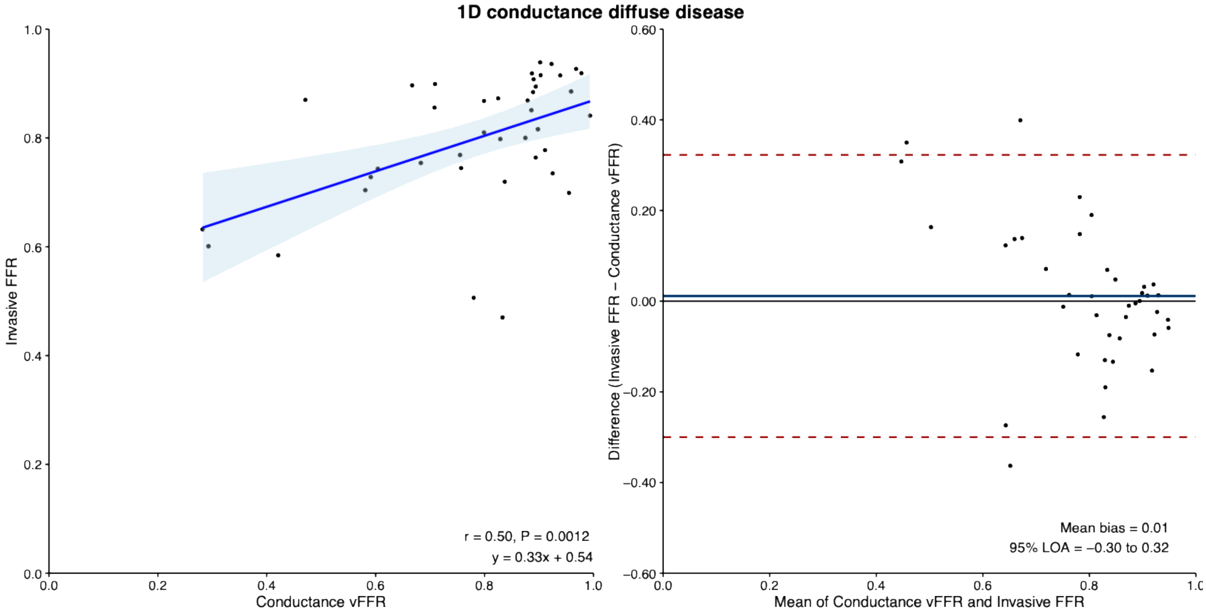

	Conductance Focal Disease		Conductance Diffuse Disease	
**Statistic**	**Value**	**95% CI**	**Value**	**95% CI**
Sensitivity	59.26%	38.80% to 77.61%	55.56%	30.76% to 78.47%
Specificity	80.65%	62.53% to 92.55%	72.73%	49.78% to 89.27%
Positive Likelihood Ratio	3.06	1.40 to 6.70	2.04	0.92 to 4.52
Negative Likelihood Ratio	0.51	0.31 to 0.82	0.61	0.34 to 1.09
Disease prevalence (*)	46.55%	33.34% to 60.13%	45.00%	29.26% to 61.51%

Positive Predictive Value (*)	72.73%	54.91% to 85.38%	62.50%	42.88% to 78.73%
Negative Predictive Value (*)	69.44%	58.28% to 78.71%	66.67%	52.91% to 78.07%
Accuracy (*)	70.69%	57.27% to 81.91%	65.00%	48.32% to 79.37%

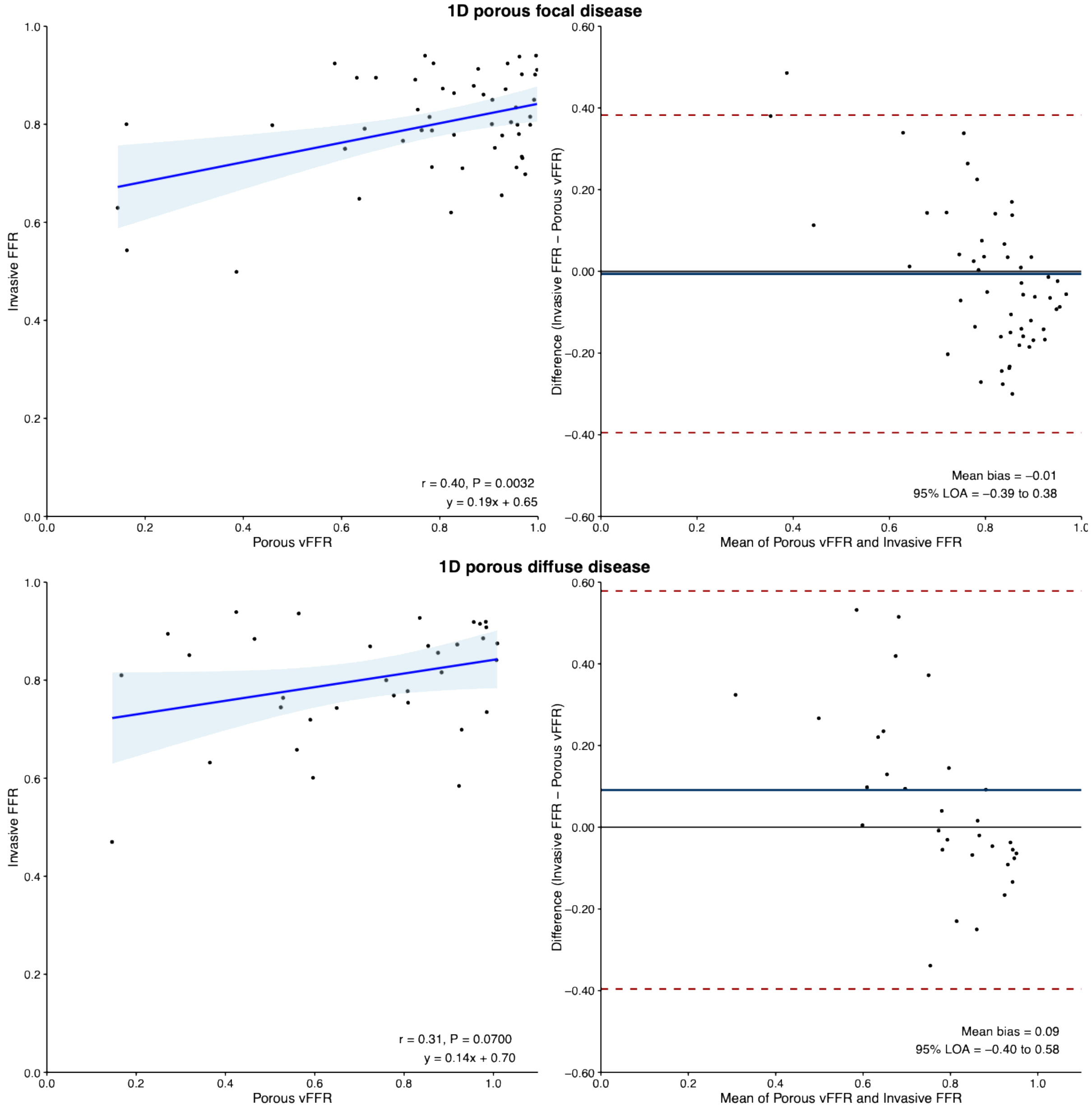

	Porous Focal Disease		Porous Diffuse Disease	
**Statistic**	**Value**	**95% CI**	**Value**	**95% CI**
Sensitivity	46.15%	26.59% to 66.63%	66.67%	38.38% to 88.18%
Specificity	70.37%	49.82% to 86.25%	63.16%	38.36% to 83.71%
Positive Likelihood Ratio	1.56	0.76 to 3.18	1.81	0.91 to 3.60
Negative Likelihood Ratio	0.77	0.50 to 1.18	0.53	0.24 to 1.17

Disease prevalence (*)	49.06%	35.06% to 63.16%	44.12%	27.19% to 62.11%
Positive Predictive Value (*)	60.00%	42.34% to 75.40%	58.82%	41.77% to 73.99%
Negative Predictive Value (*)	57.58%	46.84% to 67.64%	70.59%	52.04% to 84.15%
Accuracy (*)	58.49%	44.13% to 71.86%	64.71%	46.49% to 80.25%

Supplementary 12 Agreement results for optimally Fourier filtered subset of cases

	No leak	Homogenous	Localised	Conductance	Porosity
Successful simulation	36 (100%)	36 (100%)	36 (100%)	33 (92%)	30 (83%)
Correlation, r	0.4, p = 0.016	0.28, p = 0.098	0.3, p = 0.075	0.28, p = 0.115	0.45, p = 0.013
Mean Bias	-0.03	-0.08	-0.03	-0.01	0.06
95% LOA	[-0.28,0.21]	[-0.41,0.26]	[-0.28,0.22]	[-0.33,0.31]	[-0.54,0.43]
Diagnostic accuracy	55.60%	52.80%	52.80%	63.60%	60.00%

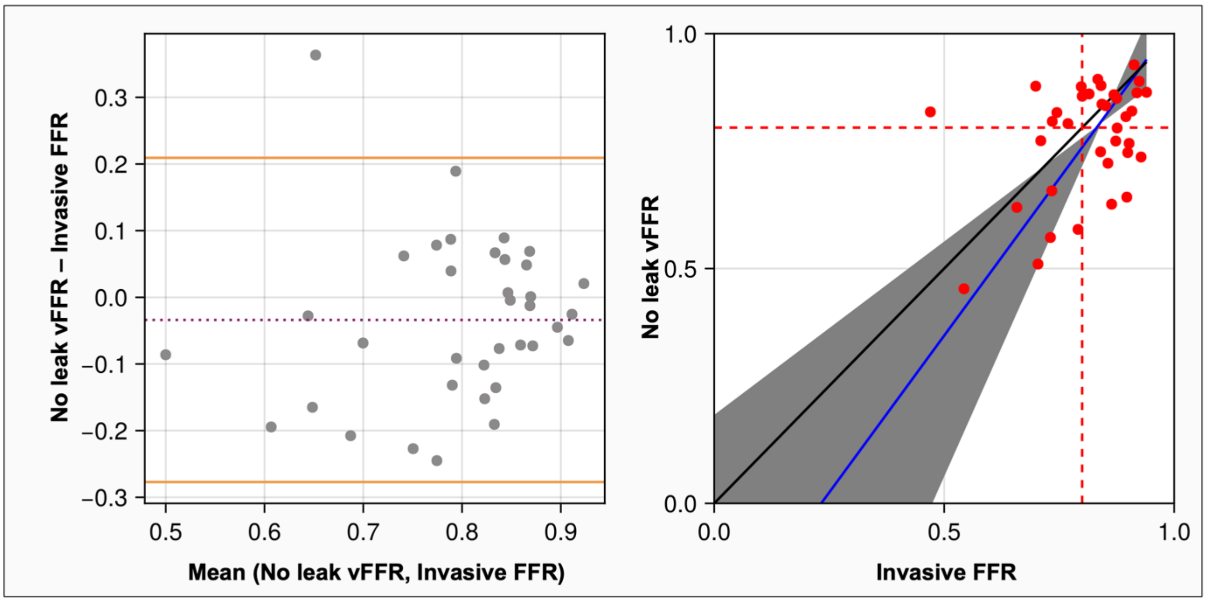

S12 figure 1. Bland Altman and Passing and Bablok regression comparing invasive FFR with no leak vFFR in the 36 case subset. No leak vFFR underestimated FFR by -0.03 (95% LOA -0.28 to 0.21). Passing and Bablok regression did not identify significant proportional or constant differences between techniques (m coefficient = 1.34 (95% CI 0.74 to 2.20), c coefficient = -0.31 (95% CI -1.04 to 0.19)).

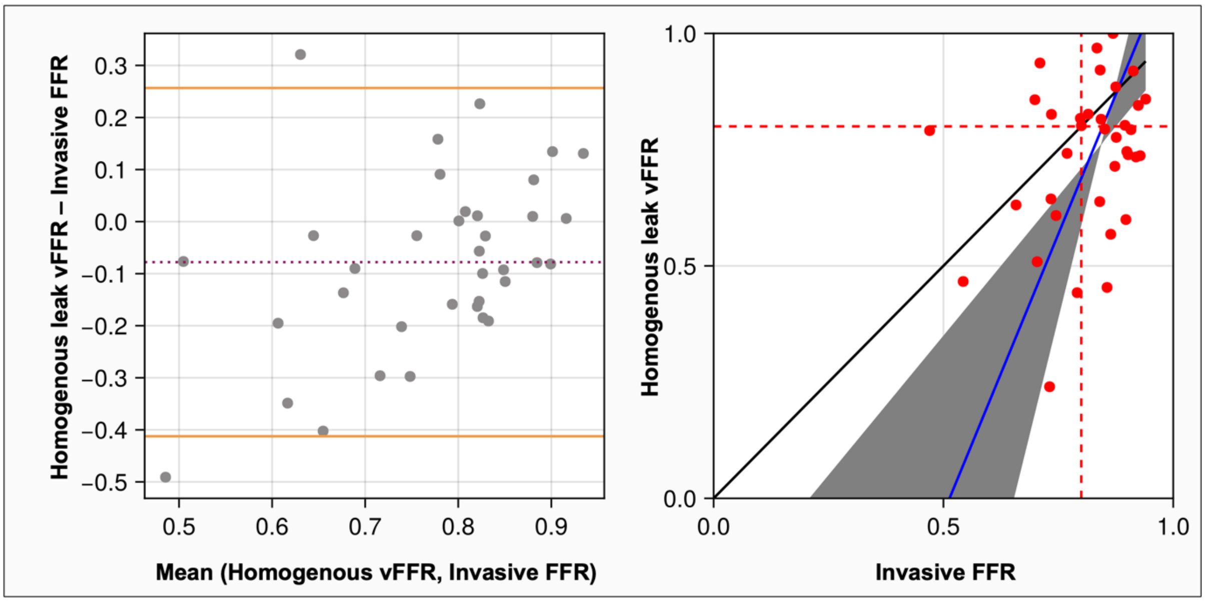

S12 figure 2. Bland Altman and Passing and Bablok regression comparing invasive FFR with homogenous leak vFFR in the 36 case subset. Homogenous leak vFFR underestimated FFR by -0.08 (95% LOA -0.41 to 0.26). Passing and Bablok regression identified significant proportional and constant differences between techniques (m coefficient = 2.40 (95% CI 1.20 to 4.01), c coefficient = -1.23 (95% CI -2.62 to -0.25)).

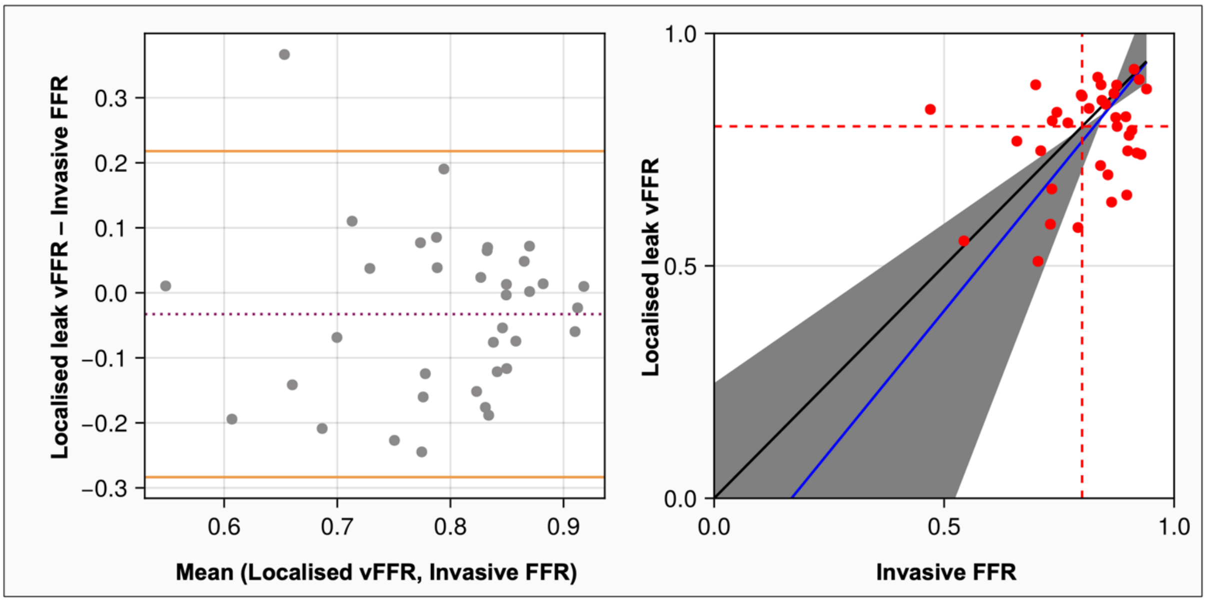

S12 figure 3. Bland Altman and Passing and Bablok regression comparing invasive FFR with localised leak vFFR in the 36 case subset. Localised leak vFFR underestimated FFR by -0.03 (95% LOA -0.28 to 0.22). Passing and Bablok regression did not identify significant proportional or constant differences between techniques (m coefficient = 1.22 (95% CI 0.69 to 2.56), c coefficient = -0.20 (95% CI -1.34 to 0.25)).

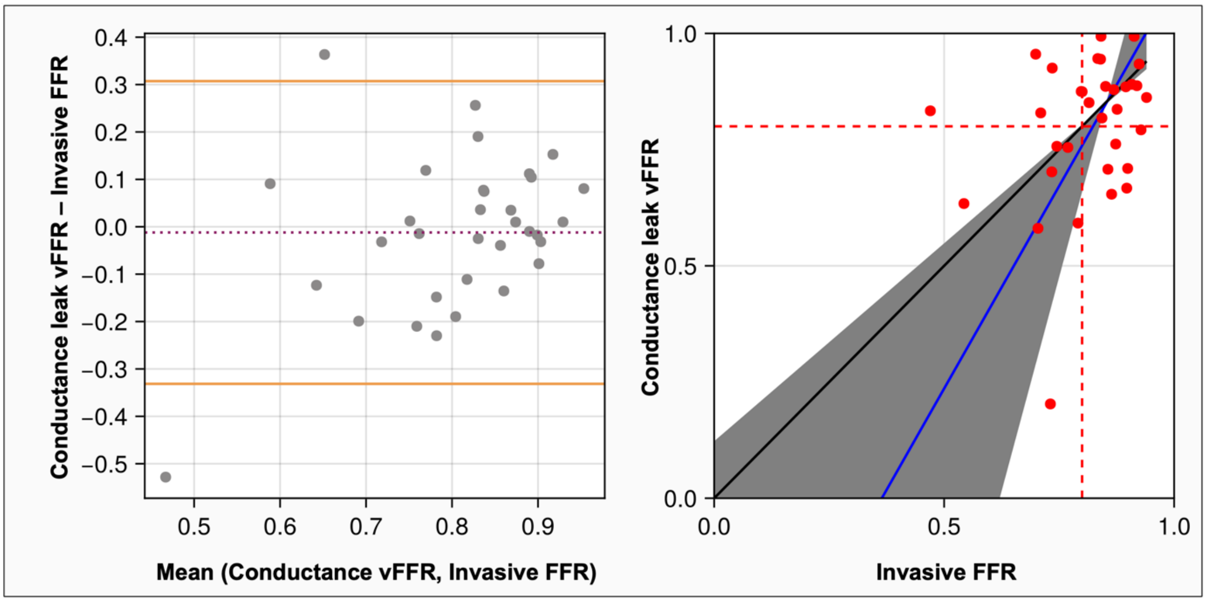

S12 figure 4. Bland Altman and Passing and Bablok regression comparing invasive FFR with conductance leak vFFR in the 36 case subset. Conductance leak vFFR underestimated FFR by -0.01 (95% LOA -0.33 to 0.31). Passing and Bablok regression did not identify significant proportional or constant differences between techniques (m coefficient = 1.74 (95% CI 0.85 to 3.68), c coefficient = -0.64 (95% CI -2.28 to 0.12)).

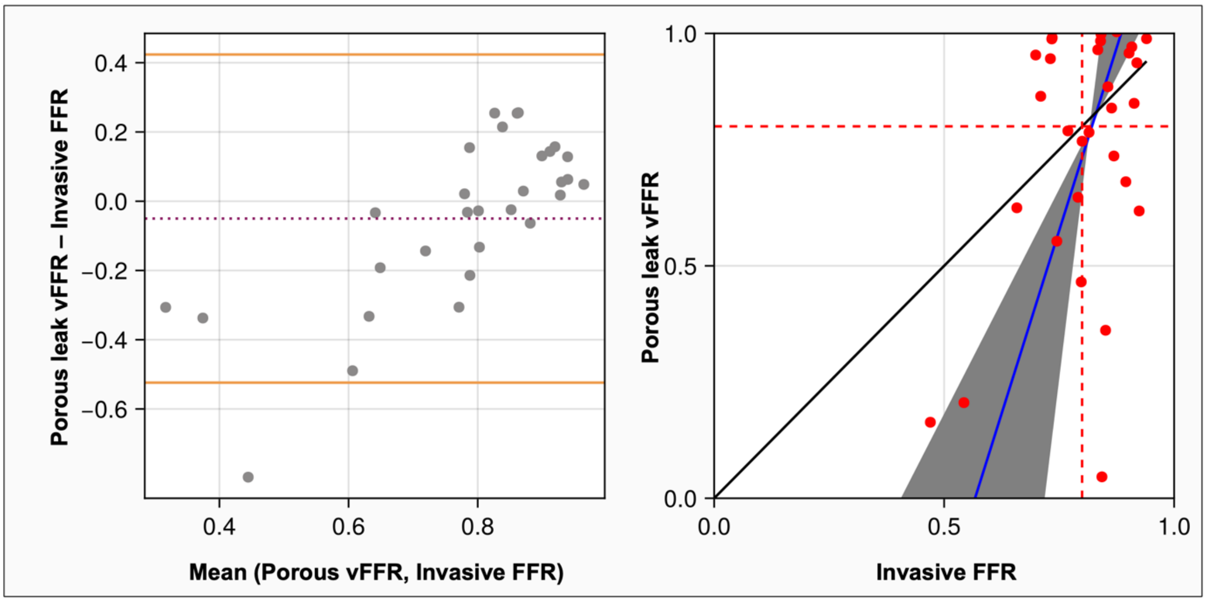

S12 figure 5. Bland Altman and Passing and Bablok regression comparing invasive FFR with porous leak vFFR in the 36 case subset. Porous leak vFFR underestimated FFR by -0.06 (95% LOA -0.54 to 0.43). Passing and Bablok regression identified significant proportional and constant differences between techniques (m coefficient = 2.83 (95% CI 1.89 to 6.03), c coefficient = -1.53 (95% CI -4.13 to -0.74)).

Supplementary 13 Agreement results for 3D vFFR

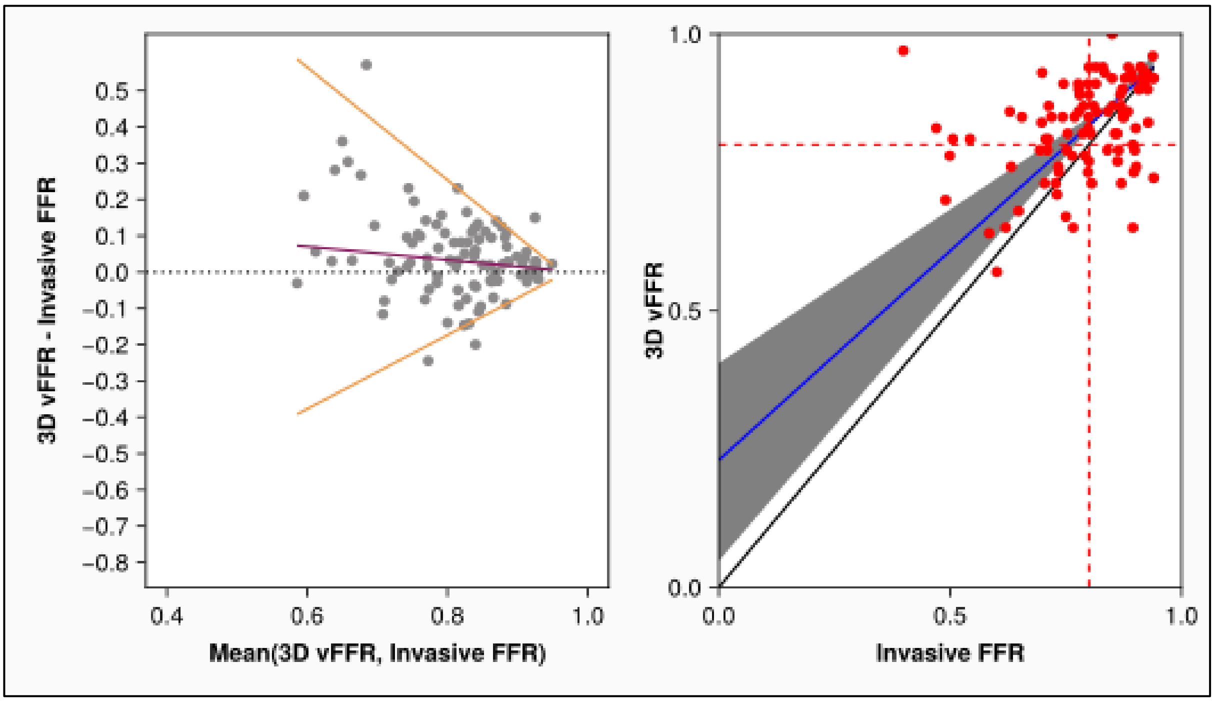

S13 figure 1. Bland Altman plots using quantile regression comparing invasive FFR with 3D vFFR. Quantile regression equation of a straight line: 2.5th centile y = 1.02x – 0.99. 50th centile y = 0.18x + 0.18. 97.5th centile y = -1.55 +1.50. Passing and Bablok regression identified significant proportional and constant differences between techniques (m coefficient = 0.76 (95% CI 0.56 to 0.98), c coefficient = 0.23 (95% CI 0.05 to 0.41)).

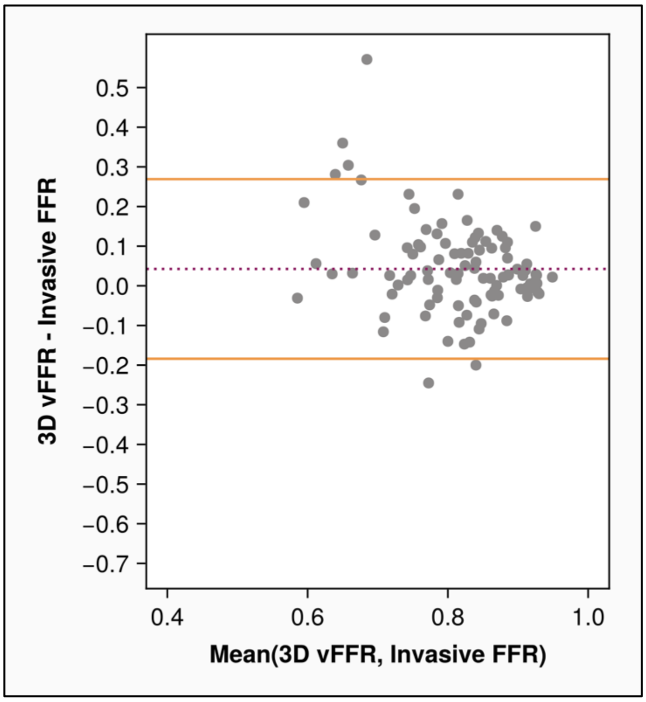

S13 figure 2. Bland Altman and Passing and Bablok regression comparing invasive FFR with 3D vFFR. 3D vFFR overerestimated FFR by 0.04 (95% LOA -0.18 to 0.27).

**Statistic**	**Value**	**95% CI**
Sensitivity	42.55%	28.26% to 57.82%
Specificity	77.36%	63.79% to 87.72%
Positive Likelihood Ratio	1.88	1.03 to 3.42
Negative Likelihood Ratio	0.74	0.56 to 0.99
Disease prevalence (*)	44.00%	
Positive Predictive Value (*)	59.62%	44.81% to 72.87%
Negative Predictive Value (*)	63.15%	56.29% to 69.52%
Accuracy (*)	62.04%	51.79% to 71.56%

Supplementary 14 Diagnostic accuracy of Pd/Pa

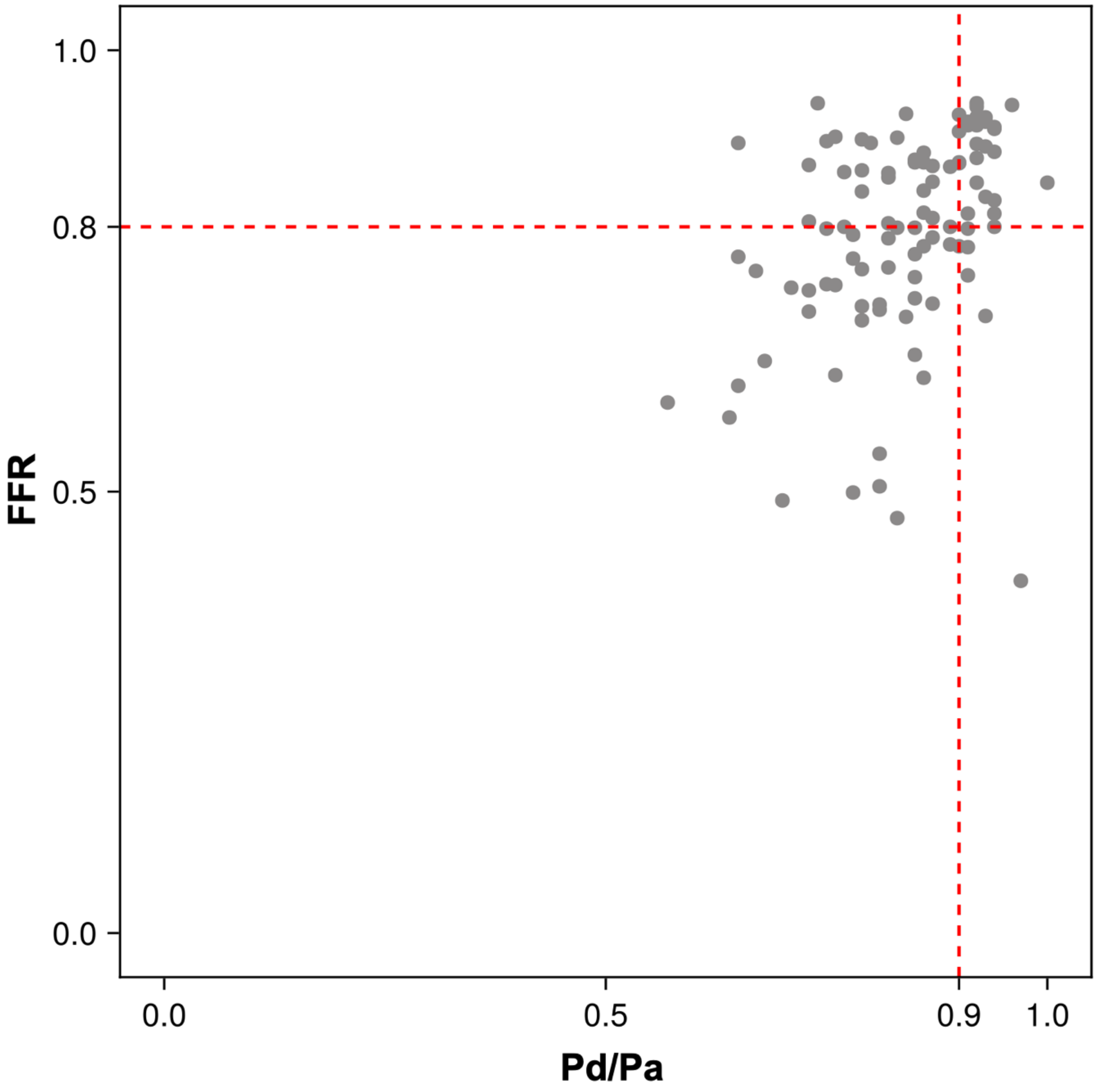

Statistic	Value	95% CI
Sensitivity	68.09%	52.88% to 80.91%
Specificity	94.44%	84.61% to 98.84%
Disease prevalence (*)	44.00%	
Positive Predictive Value (*)	90.59%	75.91% to 96.71%
Negative Predictive Value (*)	79.02%	71.17% to 85.18%
Accuracy (*)	82.85%	74.06% to 89.62%

Supplementary 15 Diagnostic accuracy results for visual assessment

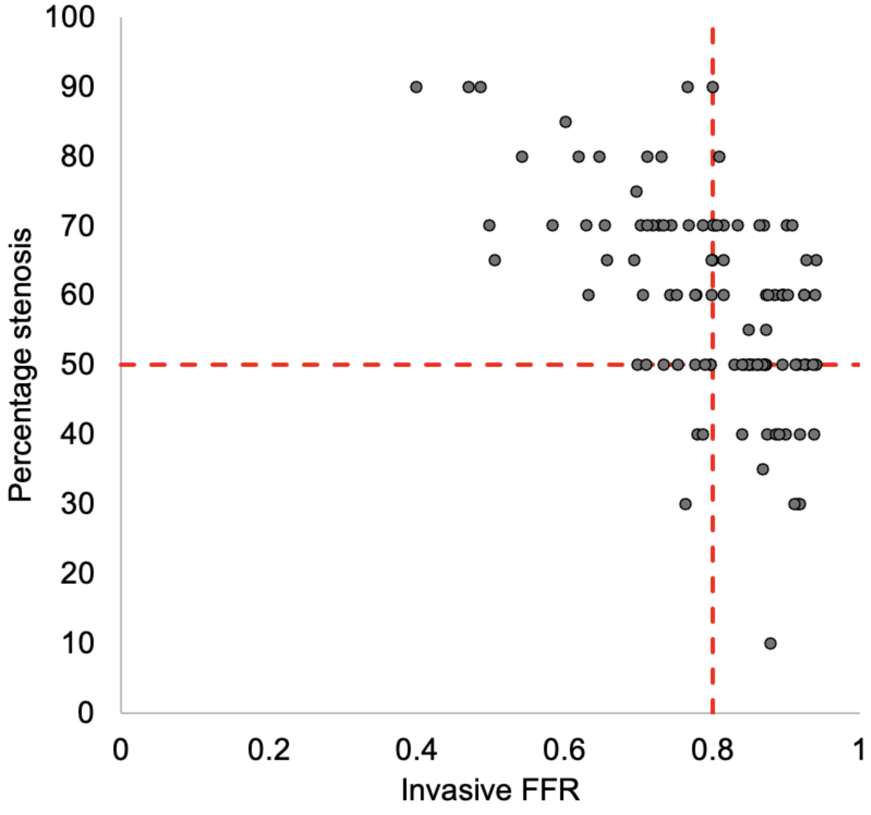

Statistic	Value	95% CI
Sensitivity	93.75%	82.80% to 98.69%
Specificity	21.82%	11.81% to 35.01%
Positive Likelihood Ratio	1.20	1.02 to 1.40
Negative Likelihood Ratio	0.29	0.09 to 0.96
Disease prevalence (*)	44.00%	
Positive Predictive Value (*)	48.51%	44.59% to 52.45%
Negative Predictive Value (*)	81.63%	57.12% to 93.68%
Accuracy (*)	53.47%	43.37% to 63.36%

* Indicates values dependent upon disease prevalence

Supplementary 16 included angiographically healthy cases
Each case contains both angiographic images used for vessel reconstruction, with the reconstruction centreline back projected as a green line. The adjacent graph shows radius as a function of centreline location in metres. The blue line represents reconstruction radius, and the green line Fourier filtered modelled healthy radius.

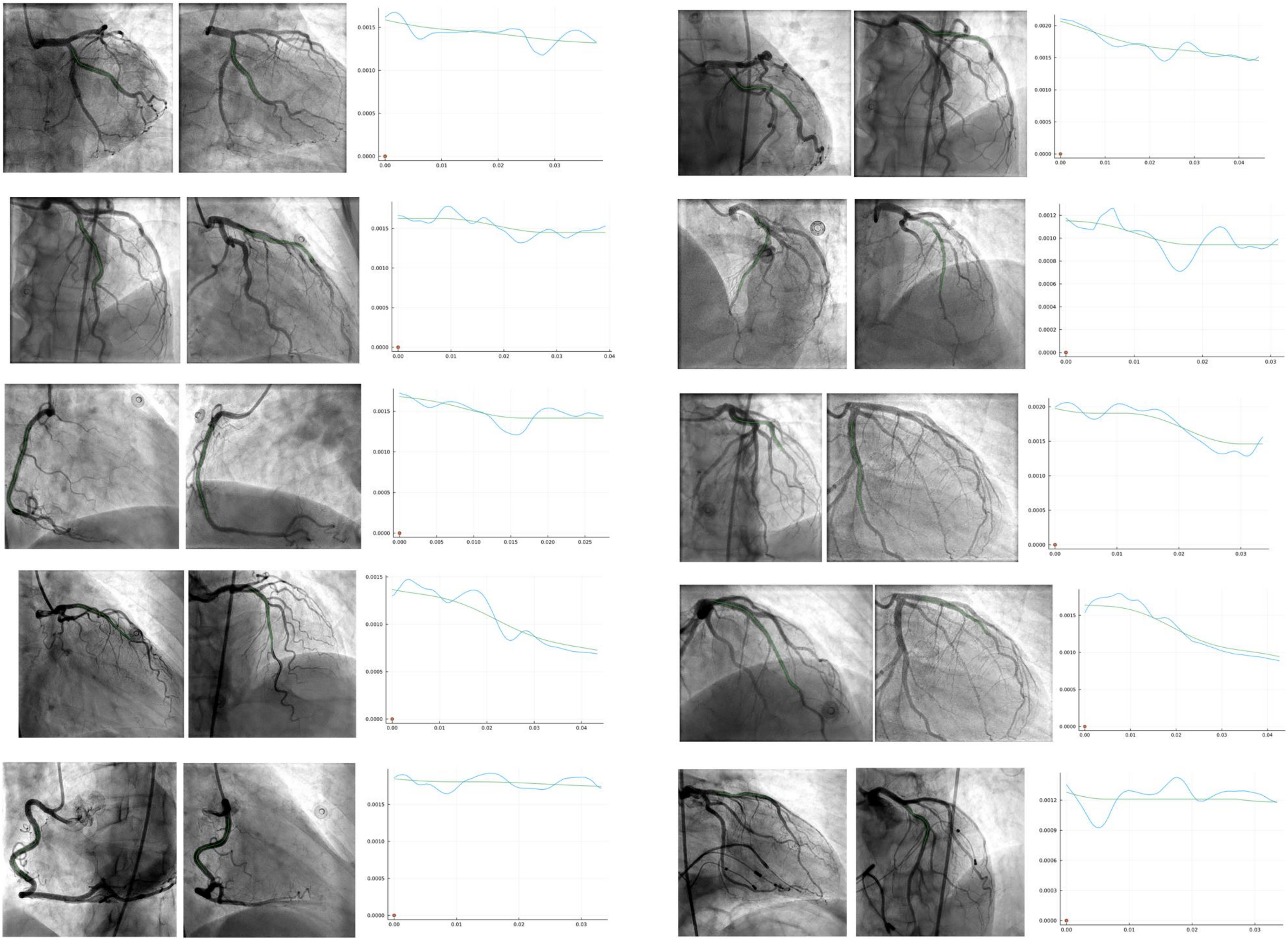

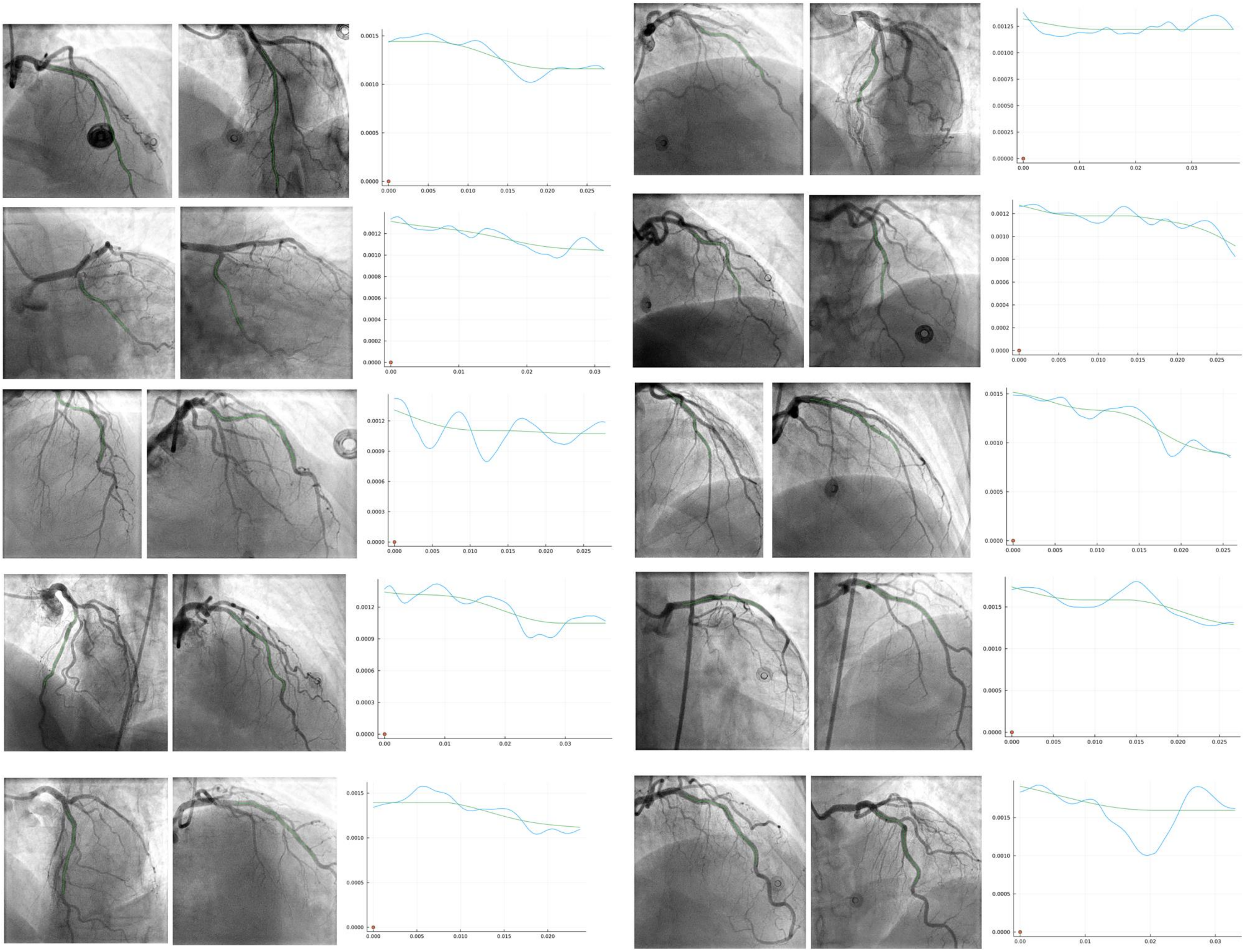
0.0015
0.0010
0.0005
0.0000
0.000 0.005 0.010 0.015 0.020 0.025
0.00125
0.00100
0.00075
0.00050
0.00025
0.00000
0.00 0.01 0.02 0.03
0.0012
0.0010
0.0008
0.0006
0.0004
0.0002
0.0000
0.00 0.01 0.02 0.03
0.0012
0.0010
0.0008
0.0006
0.0004
0.0002
0.0000
0.000 0.005 0.010 0.015 0.020 0.025
0.0012
0.0009
0.0006
0.0003
0.0000
0.000 0.005 0.010 0.015 0.020 0.025
0.0015
0.0010
0.0005
0.0000
0.000 0.005 0.010 0.015 0.020 0.025
0.0012
0.0009
0.0006
0.0003
0.0000
0.00 0.01 0.02 0.03
0.0015
0.0010
0.0005
0.0000
0.000 0.005 0.010 0.015 0.020 0.025
0.0015
0.0010
0.0005
0.0000
0.000 0.005 0.010 0.015 0.020
0.0015
0.0010
0.0005
0.0000
0.00 0.01 0.02 0.03

Supplementary 17 Demographics, agreement and diagnostic accuracy in healthy arteries

Patient Demographics (n = 14)	
Age, y	60.4 ±11.3
Male	5 (36%)
Obese	3 (23%)
Smoker	2 (20%)
Comorbidities	
Hypertension	3 (25%)
Chronic coronary syndrome	12 (86%)
Hypercholesterolaemia	6 (43%)
Medication	
Aspirin	6 (43%)
Second anti-platelet	4 (29%)
Statin	12 (86%)
RAAS inhibitor	3 (21%)
Calcium channel-blocker	6 (46%)
Vessel characteristics (n = 20)	
LAD	14 (67%)
LCx	4 (19%)
RCA	2 (10%)
Visual diameter stenosis	5% [0% - 15%]
FFR	0.89 [0.84 - 0.96]
Number of lesions with FFR ≤0.80	0

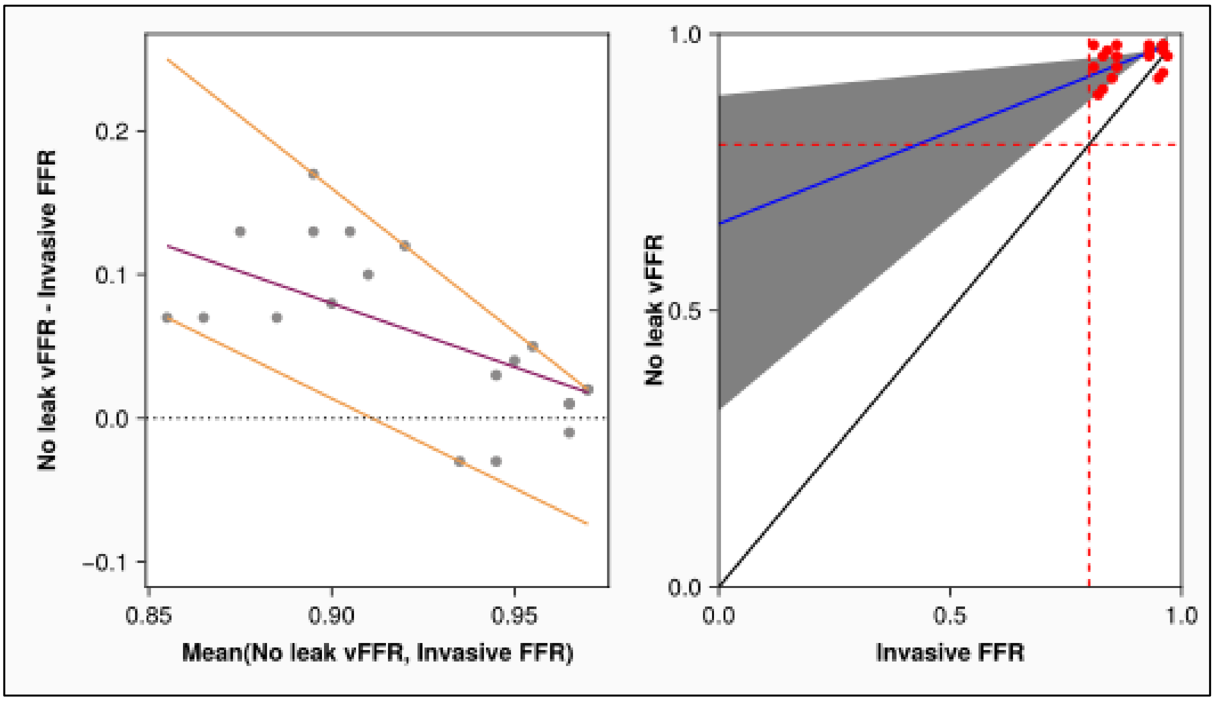

S17 figure 1. Bland Altman and Passing and Bablok regression comparing invasive FFR with no leak vFFR in the angiographically healthy vessel cohort. No leak vFFR showed a general trend of underestimating invasive FFR. Diagnostic accuracy was 100%. There was no evidence of a relationship between invasive MVR and overall agreement (Spearman's Rho = -0.047, p = 0.85).

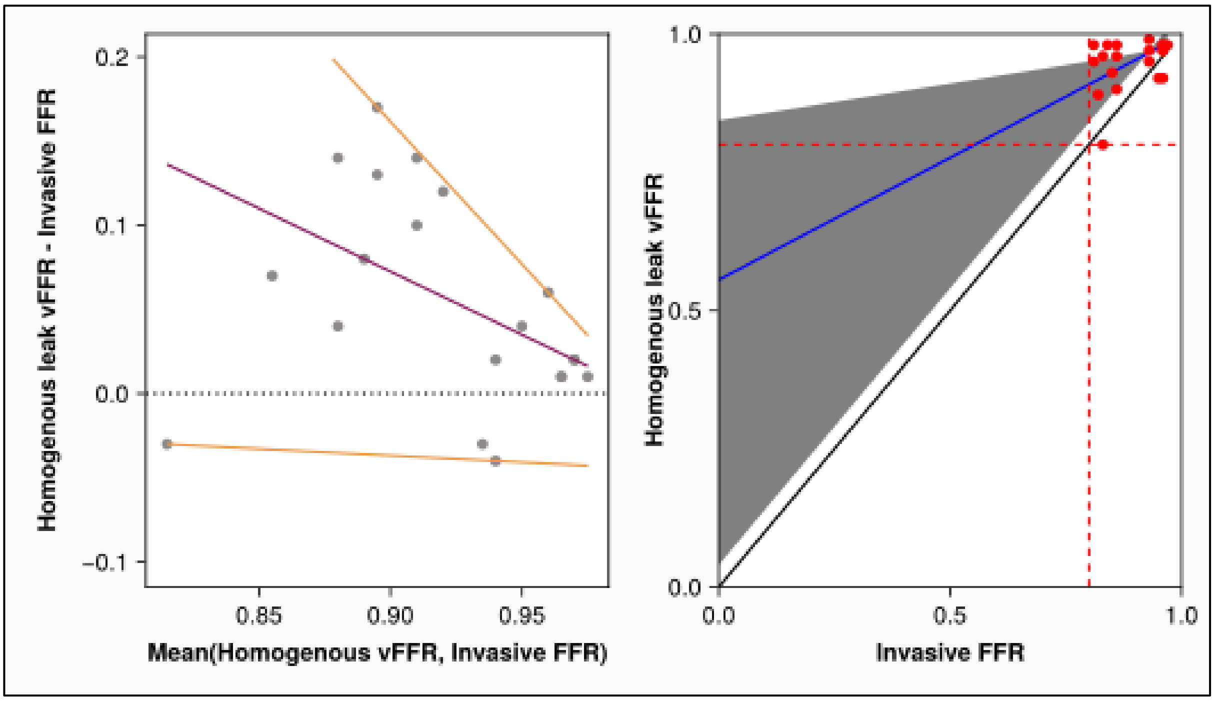

S17 figure 2. Bland Altman and Passing and Bablok regression comparing invasive FFR with homogenous vFFR in the angiographically healthy vessel cohort. Homogenous vFFR showed a general trend of underestimating invasive FFR. Diagnostic accuracy was 100%. There was no evidence of a relationship between invasive MVR and overall agreement (Rho = -0.075, p = 0.75).

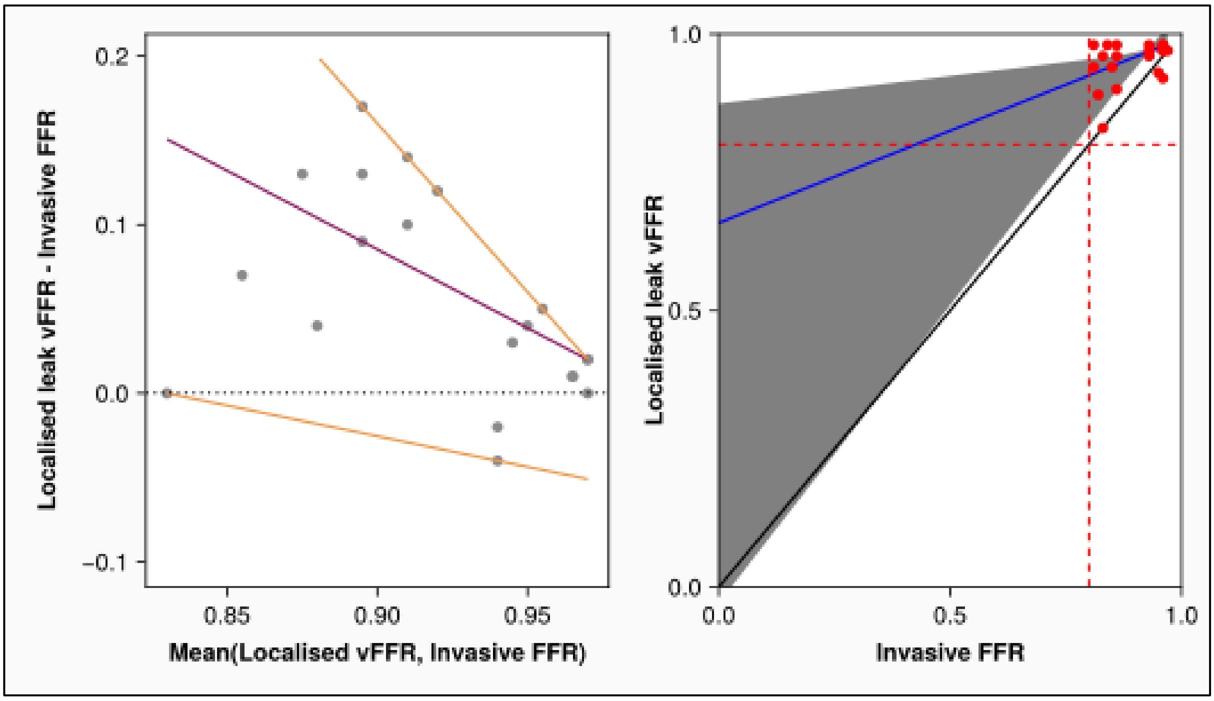

S17 figure 3. Bland Altman and Passing and Bablok regression comparing invasive FFR with localised vFFR in the angiographically healthy vessel cohort. Localised vFFR showed a general trend of underestimating invasive FFR. Diagnostic accuracy was 100%. There was no evidence of a relationship between invasive MVR and overall agreement (Rho = 0.044, p = 0.86).

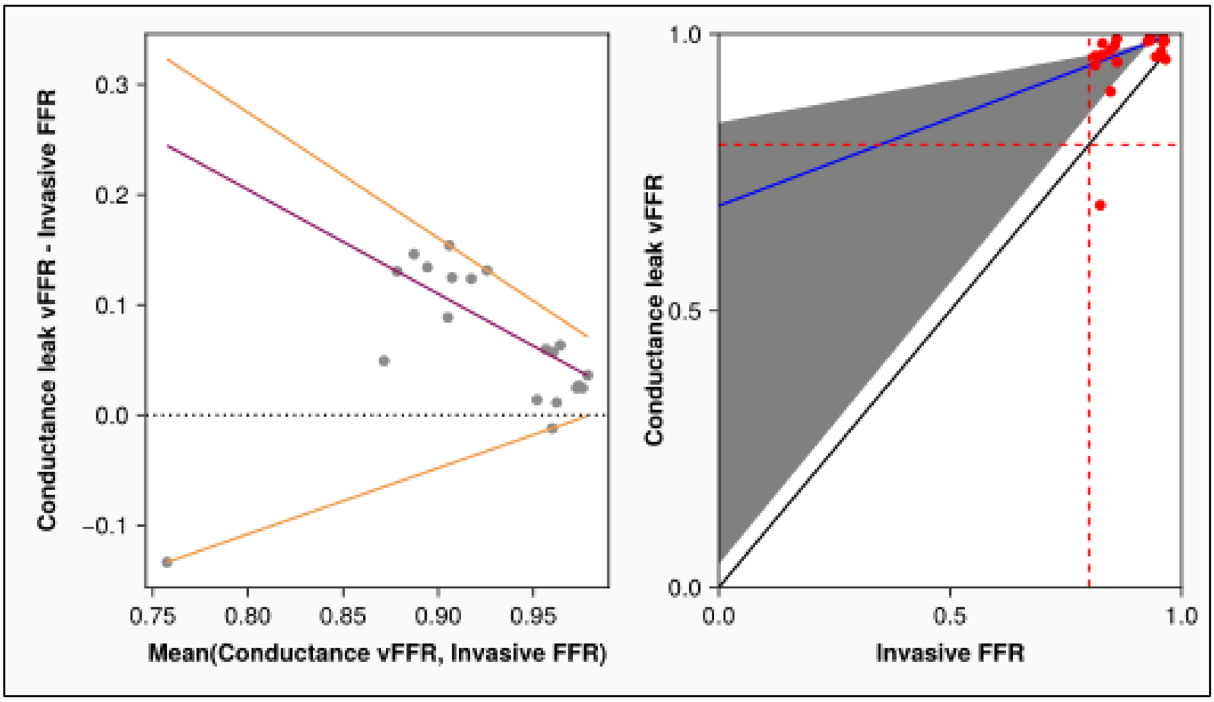

S17 figure 4. Bland Altman and Passing and Bablok regression comparing invasive FFR with conductance vFFR in the angiographically healthy vessel cohort. Conductance vFFR showed a general trend of underestimating invasive FFR. Diagnostic accuracy was 95%. There was no evidence of a relationship between invasive MVR and overall agreement (Rho = 0.036, p = 0.88).

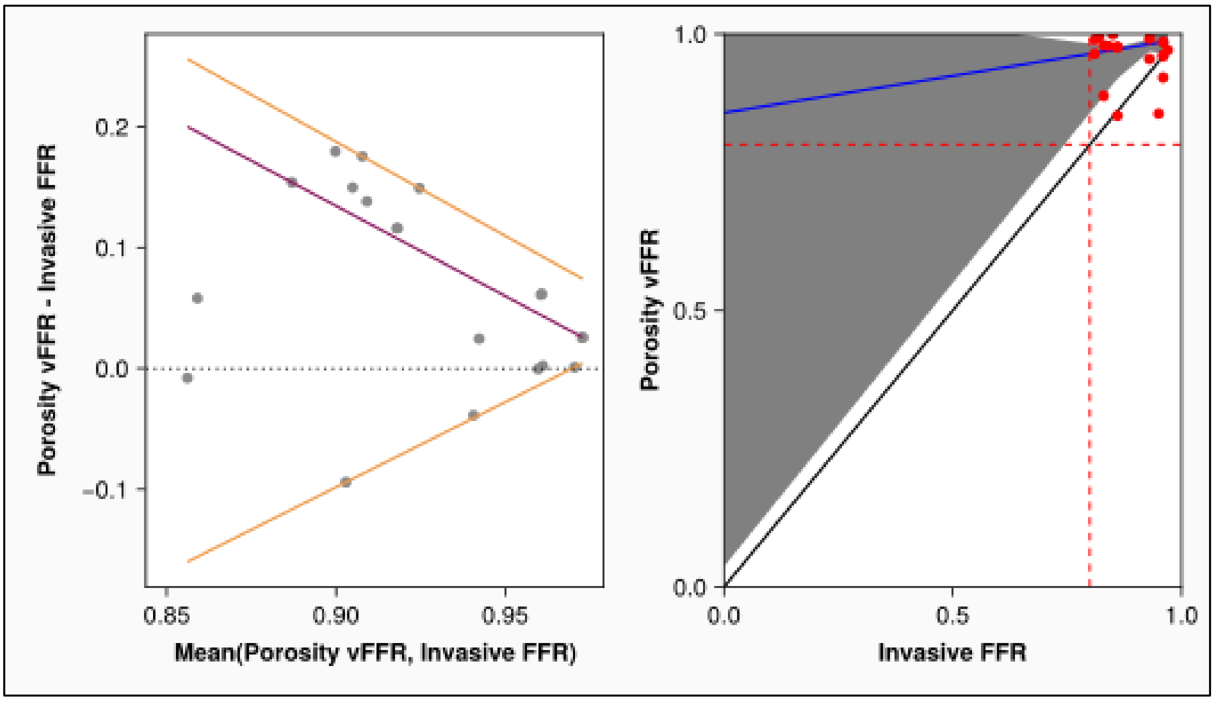

S17 figure 3. Bland Altman and Passing and Bablok regression comparing invasive FFR with porosity vFFR in the angiographically healthy vessel cohort. Porosity vFFR showed a general trend of underestimating invasive FFR. Diagnostic accuracy was 100%. There was no evidence of a relationship between invasive MVR and overall agreement (Rho = 0.18, p = 0.45).
